# The continued evolution of the L2 cephalosporinase in *Stenotrophomonas maltophilia*: a key driver of beta-lactam resistance

**DOI:** 10.1042/BCJ20240478

**Published:** 2025-01-30

**Authors:** Sylvia A. Sapula, Yu Wang, Bradley J. Hart, Jonathan J. Whittall, Henrietta Venter

**Affiliations:** UniSA Clinical and Health Sciences, Health and Biomedical Innovation, University of South Australia, Adelaide, SA 5000, Australia

**Keywords:** antimicrobial resistance, beta-lactamase, cephalosporinases, cephalosporins, extended-spectrum-beta-lactamases (ESBLs), L2 beta-lactamase

## Abstract

The *Stenotrophomonas maltophilia* L2 cephalosporinase is one of two beta-lactamases that afford *S. maltophilia* beta-lactam resistance. With the overuse of beta-lactams, selective pressures have contributed to the evolution of these proteins, generating proteins with an extended spectrum of activity. Variant L2 cephalosporinases have been detected, as has their distribution into two main clades (clades 1 and 2). Comprehensive analysis of six L2 variants, cloned into pET41a(+) and expressed in *Escherichia coli* BL21(DE3) cells, revealed that clade 1 variants exhibited higher ceftazidime resistance compared to clade 2. Notably, the Sm5341 L2 variant, carrying a Phe72Ile variation, displayed a significantly reduced resistance profile across all substrates tested, suggesting a key role of Phe72 in enzymatic activity. An Ile72Phe substitution in the pET41a(+) based Sm5341_L2 variant resulted in a gain-of-function for this protein, confirming the role of Phe72 in the activity of L2. Furthermore, residue interaction network analysis elucidated a pi–cation interaction between Tyr272 and Arg244, which may potentially be stabilizing the enzyme and its binding site. The presence of Tyr272 in clade 1 variants correlates with higher ceftazidime affinity, contrasting Asp272 in clade 2 variants. Displaying lower *K*_m_ values and higher *k*_cat_*/K*_m_ ratios, clade 1 L2 enzymes demonstrated a higher binding efficiency and greater catalytic efficiency for most of the substrates assessed. These results indicate that L2 enzymes are continuing to evolve and adapt to a selective environment fuelled by the overuse of beta-lactams. This adaptation may signal the beginning of an evolutionary process yielding variant L2 cephalosporinases with extended substrate profiles.

## Introduction

*Stenotrophomonas maltophilia* is a Gram-negative bacterium first discovered in 1943 [1]. Since then, it has emerged globally to become an important nosocomial opportunistic pathogen [2,3]. Commonly multidrug-resistant, *S. maltophilia* infections in immunocompromised patients are associated with a high mortality rate ranging from 14% to 69% [4], with pneumoniae and bacteremia presenting as the most common clinical manifestations associated with this bacterium [5]. At particular risk of *S. maltophilia,* infections are those admitted to intensive care units, recently exposed to antibiotics, undertaking immunosuppressant treatment, and those suffering from cystic fibrosis, amongst others [6]. Its success as an opportunistic pathogen largely stems from its intrinsic resistance, which makes it resistant against many commonly used antibiotics, including most beta-lactams [4].

Resistance to beta-lactams is mediated by two chromosomally encoded, potent, and inducible beta-lactamases, L1 and L2 [7,8]. L1 is a Zn^2+^ dependent metallo-beta-lactamase (MBL), capable of hydrolyzing most beta-lactams including the carbapenems imipenem and meropenem. L2 is a serine-based class A clavulanic acid-sensitive cephalosporinase which can inactivate penicillins, cephalosporins, and monobactams such as aztreonam [9–11]. Overuse of antibiotics, especially extended-spectrum beta-lactams, has resulted in the continual evolution of beta-lactamases such as L2, a member of the extended-spectrum-beta-lactamases (ESBL), with an increasing number of L2 variants being identified [9,12]. As illustrated by the CTX-M family of ESBLs, allelic variations have led to the development of the CTX-M-15 variant, which due to its greater substrate specificity, is now one of the most prevalent CTX-M variants globally and poses a significant challenge in combating antimicrobial resistance [13]. The continual evolution of L2, an already powerful ESBL, may see the development of a variant that is active against all beta-lactams. Assessment of L2 enzymes has not only revealed allelic variation between these but also that these ESBLs cluster into two distinct clades, indicative of evolutionary divergence [9]. Such divergence may also represent a difference in both the structure and the function of L2 proteins within these two different clades.

The work undertaken in this study aimed to further assess allelic variations within the L2 beta-lactamase and to determine if L2 proteins allocated to different clades vary in their enzymatic activity. To this end, beta-lactam resistance within 11 *S*. *maltophilia* isolated from aged care facilities was assessed. Amino acid variations of the L2 beta-lactamases were determined, and three variants from each clade were selected for the in-depth analysis. The kinetics of the purified enzymes were compared, and a residue interaction network (RIN) was generated to explore residue variations and their potential role in enzymatic activity. Finally, to further assess the L2 active site, site-directed mutagenesis was carried out, and the role of the highly conserved Phe72 was assessed. The findings in this study provide an important insight into the function of these beta-lactamases which may aid in the ongoing development of antibiotics and inhibitors targeting these and other important ESBLs.

## Results

### Uncharacterized L2 variants were identified from healthcare-related *S. maltophilia* isolates

**Figure 1:**
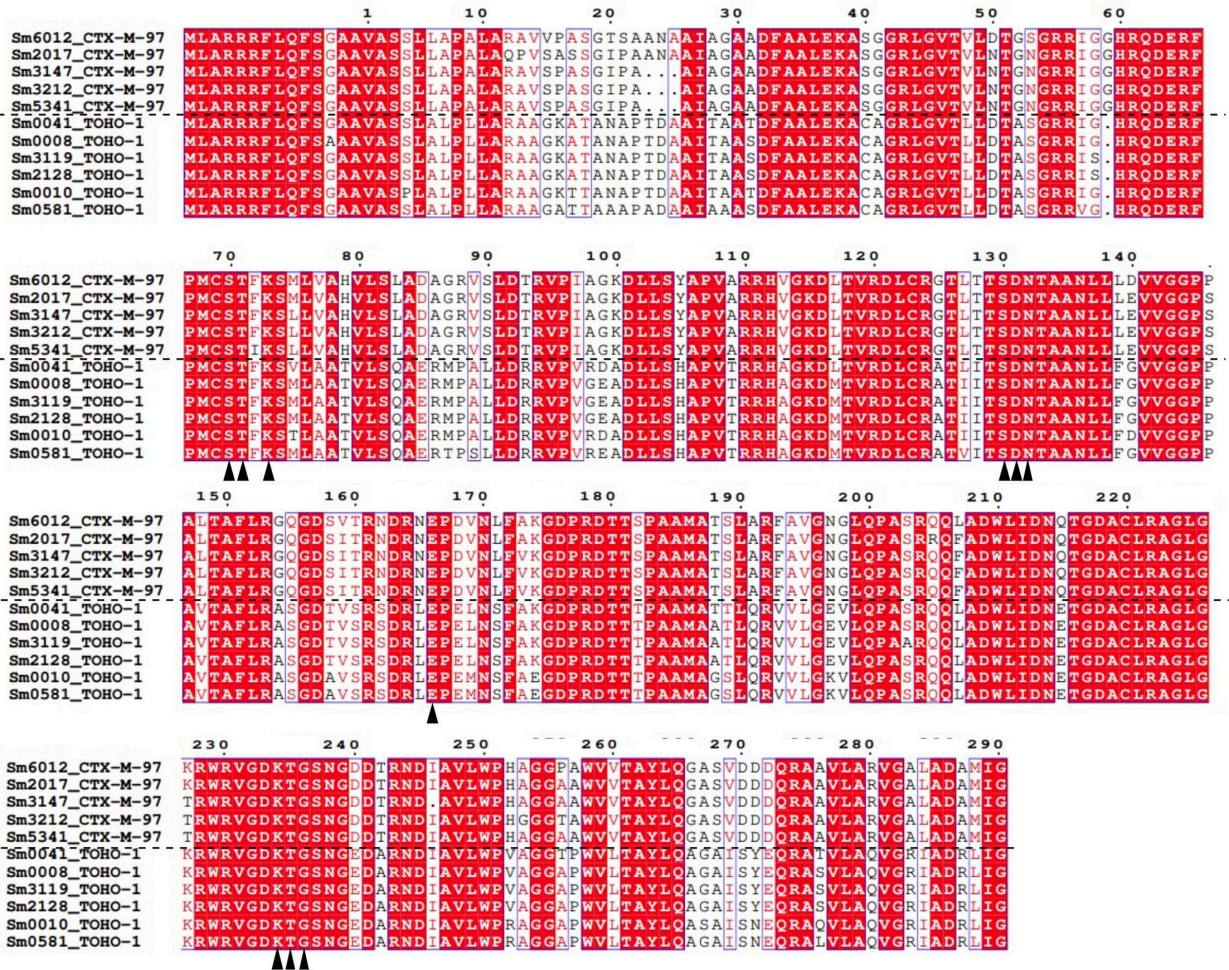
Multiple sequence alignment of 11 L2 protein sequences from *S. maltophilia* isolated from aged care facilities. Protein sequences were aligned using ClustalW [14] and visualized using ESPript v3.0 [15]. Red highlight and red residues indicate residues of identity or similarity, non-highlighted residues show non-matching amino acids, and dots represent gaps in the alignment. Highly conserved residues are marked with a black triangle.

Initial protein sequence analysis of 11 L2 beta-lactamases carried by *S. maltophilia* isolates recovered from aged care facilities identified these as either CTX-M-97 or TOHO-1 like beta-lactamases. An alignment of these revealed that CTX-M-97 like L2 proteins aligned more closely with other CTX-M-97 like L2 proteins and differed from those annotated as TOHO-1 like beta-lactamases (Figure 1).

To assess and determine possible amino acid variations within the 11 L2 beta-lactamases, each L2 protein sequence was used as a query in a BLASTp search. Three L2 sequences, belonging to Sm5341, Sm0010, and Sm3119, contained unique SNPs (Table 1).

**Table 1:** Three new L2 variants determined in *S. maltophilia* assessed in this study.

L2 beta-lactamase	Accession number	Strain	Amino acid variation
Sm2017_CTX-M-97	WP_046432165.1	*	
Sm3212_CTX-M-97	WP_057494722.1	*	
Sm6012_CTX-M-97	WP_006438473	*	
Sm5341_CTX-M-97	WP_308306450.1	Sm5341	F72I
Sm3147_CTX-M-97	WP_053461165.1	*	
Sm0010_TOHO-1	WP_308305346.1	Sm0010	M/V/L/I/P75T
Sm0041_TOHO-1	WP_099552815.1	*	
Sm2128_TOHO-1	WP_005414283	*	
Sm3119_TOHO-1	WP_308310342.1	Sm3119	S217A
Sm0581_TOHO-1	WP_049429565.1	*	
Sm0008_TOHO-1	WP_012481073.1	*	

*indicates sequence found in multiple species.

**Figure 2:**
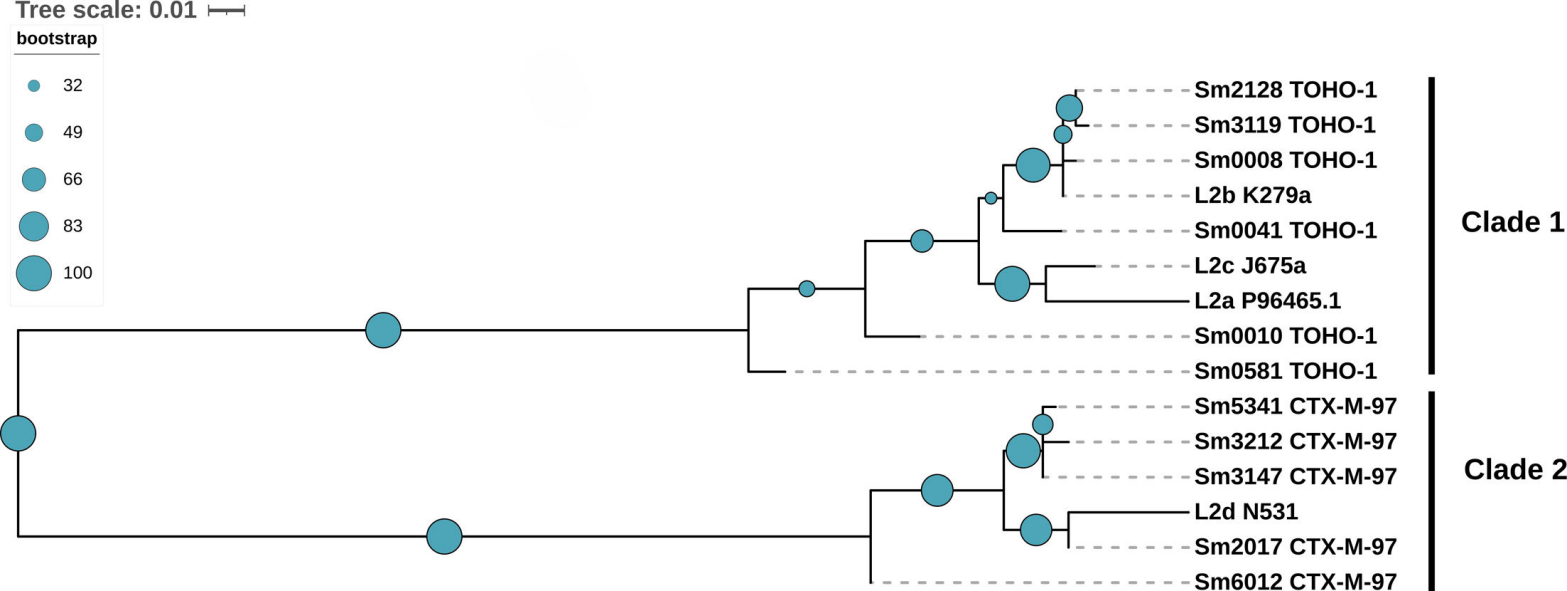
TOHO-1 like and CTX-M-97 like L2 clustering into two distinct clades characteristic of this enzyme. A maximum likelihood phylogenetic tree construction based on the ClustalW [14] alignment of protein L2 beta-lactamase sequences assessed in this study and L2 reference sequences. Bootstrapping was performed with 100 replicates and the value indicated with filled blue circles. The phylogenetic tree was viewed and annotated in iTOL v6.0 [17].

The presence of L2 beta-lactamase variants has been observed [16] as has their distribution within two distinct clades [9]. To investigate clustering, a phylogenetic tree was constructed using L2 proteins assessed in this study along with established reference L2 proteins (L2a, L2b, L2c, and L2d) [10,16]. Two distinct clades were observed, with L2a, L2b, and L2c clustering with the L2 variants initially identified as TOHO-1 like beta-lactamases (clade 1) (Figure 2). L2d was the only reference protein clustering with CTX-M-97 like proteins, which are made up of clade 2. To further analyze the prevalence of L2 beta-lactamases within each clade, 600 L2 protein sequences downloaded from National Center for Biotechnology Information (NCBI), in addition to the 11 L2 beta-lactamases assessed here, were used to generate a maximum likelihood phylogenetic tree (online supplementary Figure S1). According to these results, clade 2 appears to form a much smaller cluster in comparison to clade 1.

To investigate the homology between the 11 L2 beta-lactamases assessed in this study and L2 reference sequences, the L2b sequence (accession no. AJ251816), obtained from the *S. maltophilia* K279a strain, which is typically used as a reference strain, was used as the query in a multiple sequence alignment and the percent identity of all determined (Table 2). Based on the lower max scores and percent identities, TOHO-1 like (clade 1) L2 protein sequences share a higher percent identity (>90%) with the L2b protein sequence. Given the clustering of L2b in clade 1, these results are not unexpected. However, displaying much lower percent identities of 70%, L2 sequences identified as CTX-M-97 like (clade 2) appear to have a more distant relationship to the query sequence L2b, suggesting different evolutionary origins and potential variations in substrate specificity.

**Table 2:** Comparison of homology between L2b K279a protein sequence (a clade 1 L2 beta-lactamase) and L2 protein sequence assessed in this study.

Description	Max score	Query cover	E value	Per. Ident	Acc. Len
Sm2128_TOHO-1	601	100%	0	99.67%	303
Sm0008_TOHO-1	601	100%	0	99.67%	303
Sm3119__TOHO-1	600	100%	0	99.34%	303
Sm0041_TOHO-1	588	100%	0	97.03%	303
L2 c_ AJ251817.1	554	100%	0	95.05%	303
Sm0010_TOHO-1	549	100%	0	94.39%	303
L2 a_ CAA69869	518	100%	0	93.07%	303
Sm0581_TOHO-1	511	100%	0	93.73%	303
Sm6012_CTX-M-97	419	100%	2.00E–152	72.70%	304
L2 d_ AJ272110.1	407	100%	2.00E–147	70.39%	303
Sm2017_CTX-M-97	407	100%	2.00E–147	70.07%	304
Sm5341_CTX-M-97	402	100%	1.00E–145	70.07%	301
Sm3212_CTX-M-97	402	100%	1.00E–145	69.74%	301
Sm3147_CTX-M-97	398	100%	5.00E–144	70.07%	300

L2b amino acid sequence Accession no. AJ251816.

*L2 proteins cloned into pET41a(+) and assessed in this study.

### Higher levels of resistance conferred by TOHO-1 like (clade 1) L2 beta-lactamases against ceftazidime

Assessment of resistance conferred by the L2 beta-lactamase in *S. maltophilia* can be complicated due to the co-presence of the L1 MBL. This MBL is highly efficient at hydrolyzing many beta-lactams including certain cephalosporins and carbapenems such as meropenem and imipenem [18,19]. However, previous studies suggest that of the compounds assessed here, both cefepime and the monobactam aztreonam are not substrates of the L1 MBL [20,21]. The study by Hu et al. [20] also confirmed that the aforementioned compounds are L2 substrates and that the L2 beta-lactamase is not very active against cefoxitin. As such, to assess the activity of L2 beta-lactamases, three TOHO-1 like (clade 1) and three CTX-M-97 like (clade 2) L2s were cloned into pET41a(+) and their activity analyzed in *E. coli* BL21(DE3) cells. As L2 beta-lactamases are classified as clavulanic acid-sensitive cephalosporinases [10], resistance against a range of cephalosporins representing generations 1–4 was assessed (Table 3).

Results of this study confirm that cefoxitin is not a substrate of L2 as the MICs of pET41a(+)_L2 constructs in *E. coli* BL21(DE3) cells were comparable to *E. coli* cells carrying an empty vector. Interestingly, despite high cefepime MICs of 128 mg/l observed in three *S. maltophilia* isolates (Table 3), MICs derived from corresponding pET41a(+)_L2 constructs in *E. coli* BL21(DE3) cells were comparatively low, with *E. coli* cells expressing the Sm3212 derived pET41a(+)_L2 construct showing the highest MIC of 16 mg/l. These results suggest that although the L2 enzyme may be able to hydrolyse cefepime, other cefepime resistance mechanisms may be utilized by these isolates. Results pertaining to aztreonam support previous studies [20] as high MICs were observed for both *S. maltophilia* isolates and *E. coli* cells expressing the corresponding pET41a(+)_L2 constructs. Analysis of ceftazidime resistance indicates that TOHO-1 like (clade 1) L2 beta-lactamases appear to confer higher rates of resistance against this cephalosporin in comparison to CTX-M-97 like (clade 2) L2 beta-lactamases. As with many other cephalosporins, the L1 MBL has also been found to hydrolyse ceftazidime [21] and may be working synergistically with L2 to confer high levels of resistance against this compound as observed in the *S. maltophilia* isolates assessed here. Finally, of the carbapenems assessed here, previous studies have shown that L2 has weak hydrolytic activity against meropenem [21], with the results presented in the study indicating that this weak hydrolytic activity may also extend to imipenem.

Comparative analysis of individual *S. maltophilia* isolates revealed Sm6012 to exhibit lower MICs against cefoxitin (16 mg/l), ceftazidime (0.5 mg/l), cefepime (16 mg/l), aztreonam (8 mg/l), and both carbapenems. Additionally, reduced beta-lactam resistance was also observed in Sm5341, with low MICs observed for ceftazidime (32 mg/l), cefepime (64 mg/l), and aztreonam (64 mg/l) for this isolate. Lower MICs were also observed for the Sm5341 L2 construct expressed in *E. coli* BL21(DE3) cells, in particular for cephalexin (16 mg/l), ceftazidime (0.5 mg/l), cefepime ( <0.5 mg/l), and aztreonam ( <0.25 mg/l).

As the Sm5341_L2 variant was observed to carry Ile in position 72 instead of Phe, which is highly conserved amongst other L2 beta-lactamases and sites amongst the known active site (Ser70-Thr71-X-Lys73), a mutant pET41a(+) based Sm5341_L2 Ile72Phe was generated, and its activity assessed. Results revealed a gain-of-function for this mutant, with increased resistance against cephalexin (MIC 128 mg/l) and aztreonam (64 mg/l). However, despite the presence of Phe in position 72, cefepime resistance remained low. To further assess the role of Phe72 in other CTX-M-like L2s, a Phe72Ile substitution was generated in pET41a(+)_Sm3212_L2. As expected, this mutation resulted in a loss of function, with *E. coli* BL21(DE3) cells carrying this construct found to be sensitive to cephalexin (MIC 8 mg/l). However, the activity of this L2 mutant was not completely abolished, as although low MICs were observed for ceftazidime (MIC 0.125 mg/l), cefepime (MIC 0.125 mg/l), and aztreonam (4 mg/l), these were higher than those observed for *E. coli* BL21(DE3) cells carrying the empty pET41a(+) vector.

**Table 3:** MICs (mg/l) of *S. maltophilia* isolates and *E. coli* BL21(DE3) cells carrying pET41a(+)-L2 beta-lactamase variants.

				Cephalosporins				Monobactam			Carbapenems	
				Cephalexin (1st gen)	Cefazolin (1st gen)	Cefoxitin (2nd gen)	Ceftazidime (3rd gen)	Ceftriaxone (3rd gen)	Cefepime (4th gen)	Aztreonam	Meropenem	Imipenem
*S. maltophilia*		CTX	Sm3212	>512	>512	256	16	>512	64	512	128	512
			Sm5341	>512	>512	256	32	512	64	64	256	512
			Sm6012	512	>512	16	0.5	256	16	8	8	64
		TOHO	Sm3119	>512	>512	512	256	512	128	>512	512	512
			Sm0041	>512	>512	512	256	512	128	512	512	512
			Sm2128	>512	512	256	128	512	128	512	256	256
*E. coli* BL21 (DE3)			pET41a	8	2	1	0.0625	<0.125	0.0156	<0.25	0.0625	0.5
		CTX	3212_L2	>512	>512	1	1	512	16	128	0.5	2
			5341_L2	16	256	1	0.5	128	<0.5	<0.25	<0.0625	0.5
			6012_L2	512	>512	1	2	512	8	64	0.125	1
		TOHO	3119_L2	256	>512	2	16	32	4	256	0.25	2
			0041_L2	256	>512	2	16	256	1	256	0.25	1
			2128_L2	256	>512	2	16	16	8	128	0.5	2
			3212_L2_F72I	8	256	ND	0.125	ND	0.125	4	ND	ND
			5341_L2_I72F	128	512	ND	0.5	ND	0.25	64	ND	ND

ND: Not determined. All MIC assays were carried out in biological triplicates.

### Hydrolytic activities of L2 beta-lactamases

Kinetic constants, *K*_m_, *k*_cat_, and *k*_cat_/*K*_m_ ratios, were determined for pET41a(+)_L2 constructs purified from *E. coli* BL21(DE3) cells (Table 4**,** online supplementary Figure S2). A comparison of all revealed generally higher *K*_m_ values for all CTX-M-97 like (clade 2) L2 beta-lactamases across all substrates tested indicating a lower affinity for these. TOHO-1 like (clade 1) L2 enzymes, with the exception of the Sm3119 derived L2, displayed lower *K*_m_ values for all three substrates, suggesting a higher affinity for these. The Sm3119 derived L2 displayed a low *K*_m_ value and subsequently high binding affinity for cephalexin; however, this L2 was noted to have similar *K*_m_ values to CTX-M-97 like (clade 2) L2 beta-lactamases for both ceftazidime and cefepime, indicating a lower affinity for these compounds.

Assessment of the *k*_cat_ values indicates that CTX-M-97 like (clade 2) L2 beta-lactamases (Sm3212 and Sm6012 derived) exhibit higher values for cefepime when compared to cephalexin and ceftazidime, indicating higher catalytic efficiency of this fourth generation cephalosporin. In comparison, TOHO-1 like (clade 1) L2 enzymes (Sm0041 and Sm3119 derived) show high *k*_cat_ values for ceftazidime with the Sm3119 L2 beta-lactamase also displaying high *k*_cat_ values for cefepime, in comparison to the other TOHO-1 like (clade 1) L2 beta-lactamases. Assessment of *k*_cat_/*K*_m_ ratios indicates that of the TOHO-1 like (clade 1) L2 enzymes, Sm0041 L2 shows the highest ratios for cephalexin, a trend also visible for Sm3119 L2.

A general comparison of all would indicate that the TOHO-1 like (clade 1) enzymes assessed in this study display lower *K*_m_ values, as such have a higher binding efficiency for the substrates assessed here. They also have higher *k*_cat_/*K*_m_ ratios, possibly indicating not only a higher affinity for these substrates but also greater catalytic efficiency for some of the substrates when compared to the CTX-M-97 like (clade 2) L2 enzymes. These results would indicate that TOHO-1 like (clade 1) and CTX-M-97 like (clade 2) L2 beta-lactamases may have distinct substrate preferences.

**Table 4:** Kinetic constants, *K*_m_ and *k*_cat_, and *k*_cat_/*K*_m_ ratios for pET41a(+)_L2 constructs purified from *E. coli* BL21(DE3) cells. Values are means ± standard errors of the means (*n* = 3).

			Cephalexin	Ceftazidime	Cefepime
*K*_m_ (μM)	CTX-M	Sm3212_L2	350±72.3	>1000	>1000
		Sm5341_L2	>1000	>1000	>1000
		Sm6012_L2	143.5±27.7	>1000	>1000
	TOHO	Sm0041_L2	17.07±5.5	740±151.6	546±54.2
		Sm2128_L2	44.17±8.9	392±131.4	769±138.9
		Sm3119_L2	11.45±4.1	>1000	>1000
*k*_cat_ (s−1)	CTX-M	Sm3212_L2	8.07±0.82	>7.8	>14.5
		Sm5341_L2	>9.48	>0.85	>2.9
		Sm6012_L2	8.74±0.5	>6.9	>22.5
	TOHO	Sm0041_L2	1.23±0.06	10.45±1.23	7.12±0.38
		Sm2128_L2	1.12±0.06	3.6±0.67	7.29±0.78
		Sm3119_L2	0.58±0.04	>18.5	>16.1
*k*_cat/_*K*_m_ (s−1 M-1)	CTX-M	Sm3212_L2	0.23x10^5^	ND	ND
		Sm5341_L2	ND	ND	ND
		Sm6012_L2	0.61x10^5^	ND	ND
	TOHO	Sm0041_L2	0.72x10^5^	0.14x10^5^	0.13x10^5^
		Sm2128_L2	0.25x10^5^	0.92x10^4^	0.95x10^4^
		Sm3119_L2	0.51x10^5^	ND	ND

### Evaluating L2 beta-lactamase variants with a ring interaction network

To assess L2 beta-lactamase allelic variations, a ring interaction network (RIN) was generated using a solved L2 structure (PDB: 1N4O), which clusters with clade 1 beta-lactamases. This RIN allowed for the visualization and assessment of possible interactions between key residues within the L2 beta-lactamase (Table 5, online supplementary Table S1). Known highly conserved residues include Ser70-Thr71-X-Lys73 which form the active site motif, the Ser130-Asp131-Asn132 “SDN” loop motif, and the Lys234-Thr(Ser)235-Gly236 “KT/SG” motif, all of which are common to all serine beta-lactamases (online supplementary Figure S3) [10,22]. Also, highly conserved are Arg164, Glu166, and Asp179 which are postulated to form the “floor” of the active site [9,10,16].

**Table 5:** Pi–cation and Pi–Pi stack interactions as determined through the RIN analysis of the L2 beta-lactamase (PDB: 1N4O – identified as a TOHO-1 like (clade 1)), with amino acid variations in CTX-M-97 (clade 2) like L2 enzymes shown.

Interaction	Source node^a^	AA variation^b^	Target^a^	AA variation^b,c^	Probability
PICATION	A/43/ARG		A/66/PHE		1
PICATION	A/244/ARG		A/272/TYR	CTX like ASP	1
PICATION	B/43/ARG		B/66/PHE		1
PICATION	B/244/ARG		B/272/TYR	CTX like ASP	1
PIPISTACK	A/33/PHE		A/60/HIS		1
PIPISTACK	A/66/PHE		A/264/TYR		1
PIPISTACK	A/72/PHE	Sm5341 ILE	A/139/PHE	CTX like LEU	1
PIPISTACK	A/229/TRP		A/259/TRP		1
PIPISTACK	B/66/PHE		B/264/TYR		1
PIPISTACK	B/72/PHE	Sm5341 ILE	B/139/PHE	CTX like LEU	1
PIPISTACK	B/229/TRP		B/259/TRP		1

^a^Chain number/residue number/residue

^b^Amino Acid variation.

^c^Variations in CTX-M-97 like L2 beta-lactamases.

**Figure 3:**
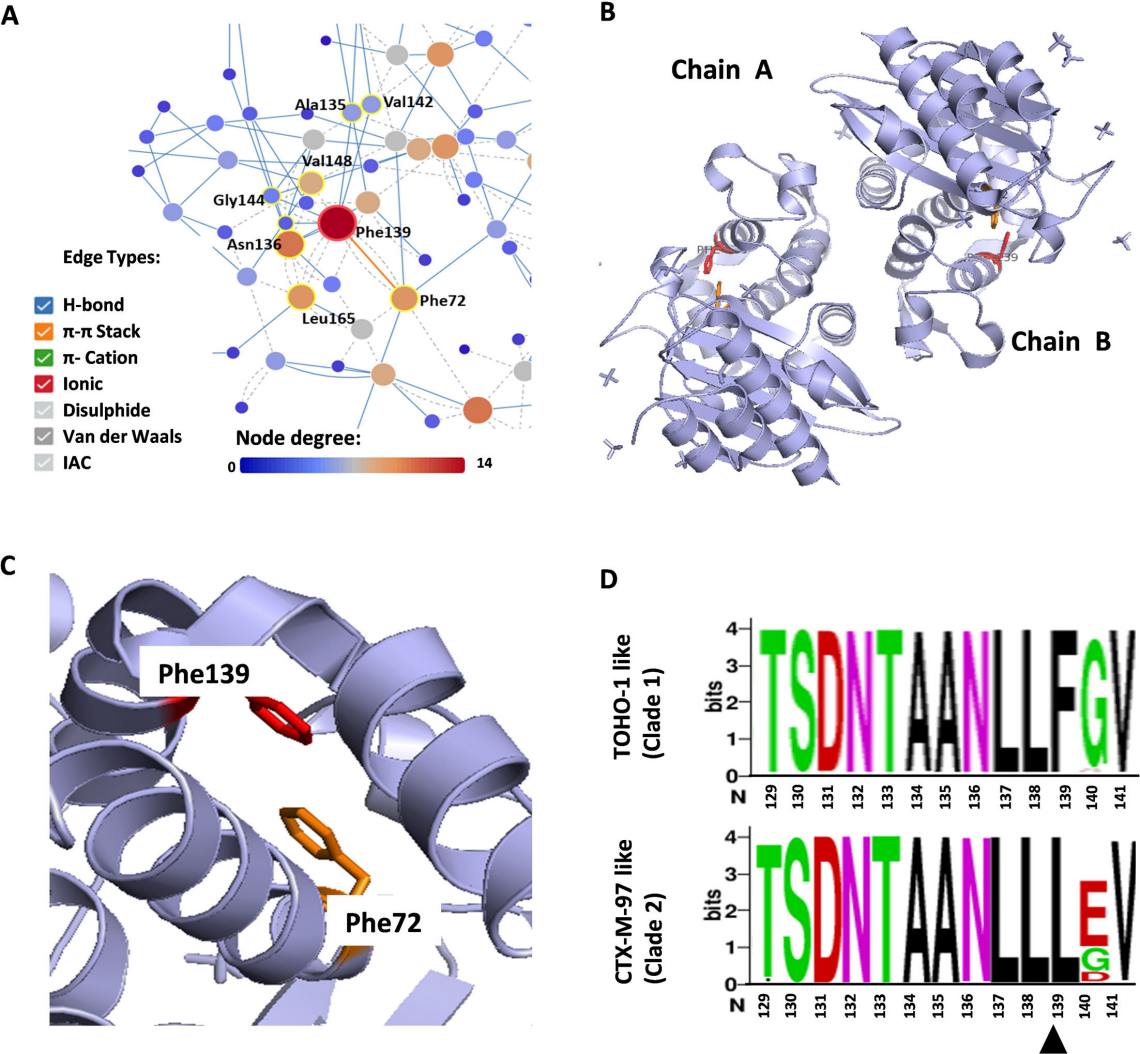
RIN showing the conserved Phe139 residue (red) and contacts within the TOHO-1 like L2 beta-lactamase (PDB: 1N4O). (**A**) Structure of the *S. maltophilia* L2 beta-lactamase (PDB: 1N4O) showing residues Phe72 (orange) and Phe139 (red) identified in a pi–pi stacking interaction in chains A and B (**B**), with a closer view in chain A (**C**). A WebLogo [23] generated using 250 TOHO-1 like (clade 1) and 200 CTX-M-97 like (clade 2) L2 protein sequences illustrating the highly conserved Phe139 in TOHO-1 like (clade 1) L2 beta-lactamases and CTX-M-97 like (clade 2) a more conserved Leu139. The height of each stack indicates sequence conservation (expressed in bits). Residues are colored according to their chemical properties (acidic – red, polar – green, neutral – purple, and hydrophobic – black) (**D**).

All of the L2 beta-lactamases assessed here carried the conserved motifs; however, as previously mentioned, the Sm5341-derived L2 contained a Phe72Ile variation. Phe72 is highly conserved amongst beta-lactamases (online supplementary Figure S3) and is found within the active site motif. It is also highly conserved in L2 beta-lactamases and, according to RIN analysis, forms a pi–pi stacking interaction with Phe139 (Figure 3). Phe139 appears to be highly conserved in TOHO-1 like (clade 1) L2 beta-lactamases but is not found in CTX-M-97 like L2s, which carry Leu in this position. In addition to forming a pi–pi stacking interaction, Phe72 and Phe139 also form Van der Waals interactions with other surrounding residues (online supplementary Table S1). These interactions may play a role in protein stabilization, with Phe72 potentially also contributing to substrate recognition and binding given its location within the active site. A protein sequence comparison of Sm5341 L2 and other CTX-M-97 like L2 beta-lactamases assessed here revealed little variation between this enzyme and the Sm3212 L2 beta-lactamase. In addition to Phe72Ile, Sm5341 L2 carries an Ala instead of a Gly in position 253 and another Ala instead of a Thr in position 256. With the exception of Phe72, residues in positions 253 and 256 have thus far not been identified as playing a role in L2 activity or stability nor are they conserved in other class A beta-lactamases; however, without experimental analysis, it is difficult to fully ascertain their importance within this beta-lactamase. Nonetheless, of the cloned L2 beta-lactamases, the resistance profile obtained for Sm5341 L2 expressed in *E. coli* BL21(DE3) cells differs with lower MIC values obtained for all of the compounds assessed in this study for this variant. Assessment of the pET41a(+) based Sm5341_L2(Ile72Phe) and Sm3212_L2(Phe72Ile) mutants confirmed the importance of Phe in position 72, with a gain-of-function observed for Sm5341_L2(Ile72Phe) construct and a loss-of-function for the Sm3212_L2(Phe72Ile) construct in *E. coli* BL21(DE3) cells. As such, these results would indicate that Phe72 plays a role in the activity of L2.

**Figure 4:**
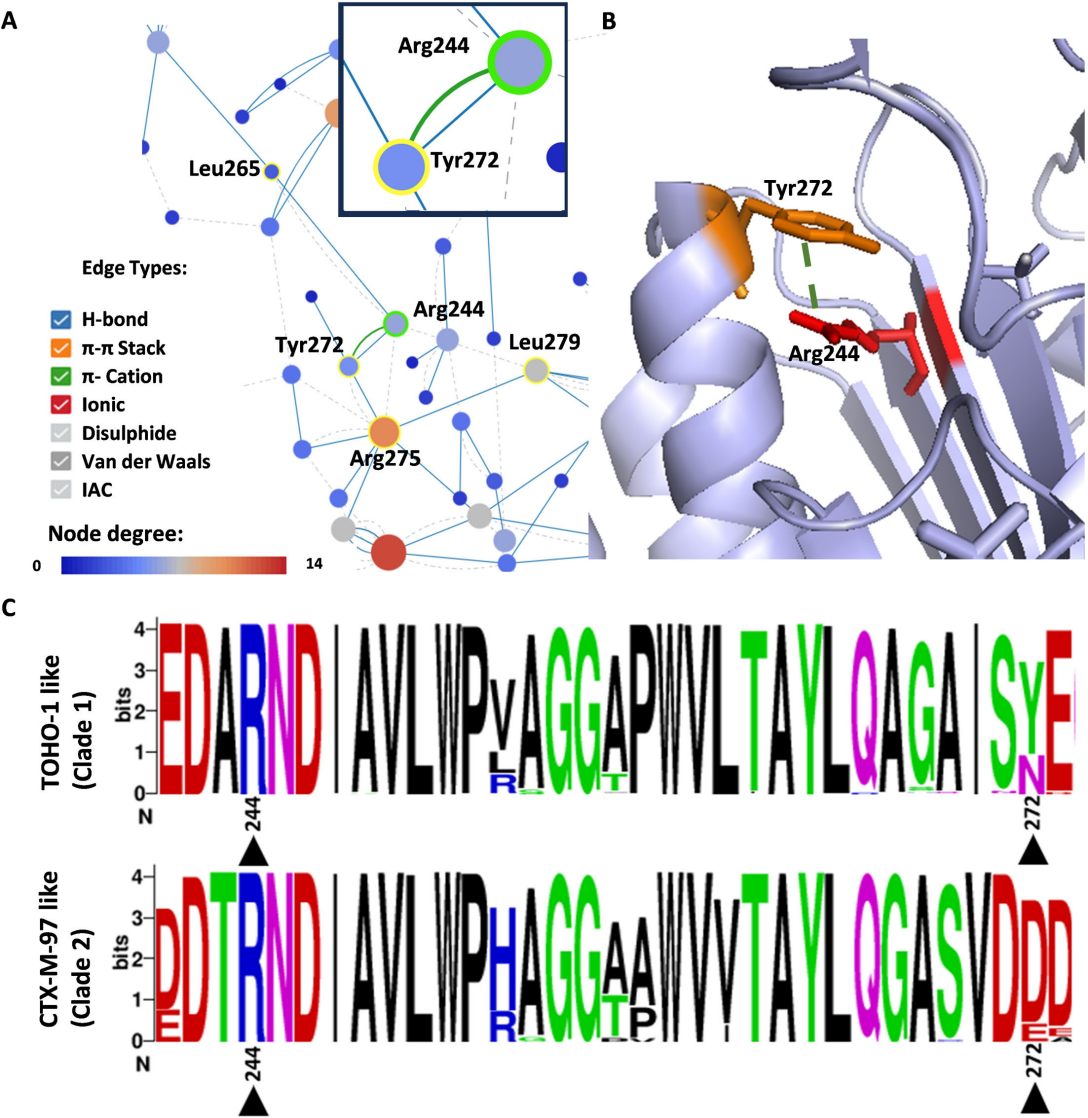
RIN showing interactions between Arg244 (light purple with green highlight) and Tyr272 (light blue with yellow highlight) in a TOHO-1 like L2 beta-lactamase (PDB: 1N4O). Green outline indicates the selected residue (Arg244) and yellow outline residues with which an interaction is formed (**A**). The pi–cation interaction between Arg244 (red) and Tyr272 (orange) is shown within the solved structure of the *S. maltophilia* TOHO-1 like L2 beta-lactamase (PDB: 1N4O) (**B**). A WebLogo [23] generated using 250 TOHO-1 like (clade 1) and 200 CTX-M-97 like (clade 2) L2 protein sequences illustrating the highly conserved Arg244 in both clade 1 and clade 2 variants, and residues found in position 272 in both clades (residues indicated with a black arrow). Sequence conservation is indicated by the height of the stack (expressed in bits). Residues are colored according to their chemical properties (acidic – red, polar – green, neutral – purple, basic – blue, and hydrophobic – black) (**C**).

In addition to the Phe139Leu variation observed for CTX-M-97 (clade 2) L2 beta-lactamases, these proteins also carry an Asp/Glu at position 272, while TOHO-1 like (clade 1) L2s carry Tyr and less frequently Asn. RIN analysis carried out here and a study assessing possible L2 inhibitors [24] revealed a pi–cation interaction between Tyr272 and Arg244 (Figure 4). This non-covalent interaction between a positively charged cation and the face of an aromatic ring may play a role in stabilizing the L2 beta-lactamase and its binding site. According to the Sharma et al. (2021) study, molecular docking of ceftazidime revealed a hydrophobic interaction with Tyr272 and this compound, in addition to the formation of a hydrogen bond between this residue and the compound. These results may indicate that Tyr272 plays a role in ceftazidime binding, potentially explaining the higher affinity and MIC values obtained for this compound and TOHO-1 like (clade 1) L2 beta-lactamases assessed here which carry this residue, in contrast to CTX-M-97 like (clade 2) proteins carrying Asp272, which showed lower MICs and binding affinity for this compound.

## Discussion

The rapid evolution and emergence of beta-lactamases represent a therapeutic challenge worldwide. Compounding this challenge is the mobilization of resistance genes from environmental to clinically relevant bacteria as exemplified by beta-lactamases such as CTX-M, TEM, and AIM-1, amongst others [25,26], a process which is largely driven by the use of beta-lactams in treatment [27,28]. The continual overuse of beta-lactam antibiotics creates a selective pressure that drives bacteria to not only acquire beta-lactamases from environmental bacteria but also to modify chromosomally encoded beta-lactamases by mutation, resulting in the formation of variants with an extended substrate profile and/or increased hydrolytic activity [29].

L2-like beta-lactamases are a family of highly efficient enzymes able to hydrolyse extended-spectrum cephalosporins such as the third-generation ceftazidime and ceftriaxone and the fourth-generation cefepime, as observed in this study and others [10,20]. L2 proteins cluster into two main clades with clade 1 representing a larger number of L2 beta-lactamases. Both clades contain L2 beta-lactamases carried by clinical *S. maltophilia* isolates; however, a recent study assessing 116 L2 sequences obtained from clinical isolates found that the majority of these clustered in clade 1 [9]. As such, it may be possible that the continual emergence of S. *maltophilia* in clinical settings is selecting for clade 1 L2 beta-lactamases. The continual expansion of clade 1 L2 enzymes, which now represent a greater number of L2 beta-lactamases, may be due to the overuse of beta-lactams and now extended-spectrum beta-lactams selecting for L2 variants which are able to hydrolyse these more efficiently.

In this study, clade 1 L2 beta-lactamases (TOHO-1 like) were found to confer higher resistance against ceftazidime and were generally found to display higher binding efficiencies for the substrates assessed, possibly indicating that these may have a distinct substrate profile. A limitation is the small number of L2 variants assessed, making it difficult to draw meaningful conclusions regarding the activity and/or substrate preference of these beta-lactamases. Nonetheless, the results indicate that these enzymes are also continuing to evolve, with specific variations possibly conferring an advantage in enzymatic activity. One of the main variations between these proteins is the presence of Phe139 in TOHO-1 like (clade 1) compared to Leu139 in CTX-M-97 like (clade 2) L2 beta-lactamases. According to the RIN, Phe is thought to be involved in a pi–pi stacking interaction with Phe72. Analysis of other class A beta-lactamases revealed that only TOHO-1 like (clade 1) L2 beta-lactamases, PER and TLA beta-lactamases carry Phe139. A hydrophobic environment is hypothesized to form from the interaction between Phe72 and Phe139 in PER, which is thought to have an impact on the interaction of this enzyme and certain antibiotics [30]. Nonetheless, in other class A beta-lactamases such as SHV, TEM, and CTX-M, position 139 is occupied with Leu or Ile, with Leu139 found within the CTX-M like (clade 2) L2 beta-lactamases. In a study assessing the L2 crystal structure (PDB:1N4O – a clade 1 L2), Phe139 was postulated to form a hydrophobic cluster together with Leu165, Pro145, and Leu169 [9]; however, the presence of Ile at position 139, and possibly Leu, may also lead to the formation of such a cluster, given the hydrophobic nature of these residues. Without experimental analysis, it is difficult to ascertain the role, Phe/Ile/Leu139 plays in enzymatic activity, although given that TOHO-1 like (clade 1) proteins display higher binding and catalytic efficiencies further exploration of this position may be warranted.

Of the above-mentioned aromatics, results from this study suggest that Phe72 is functionally important in L2 beta-lactamases. Phe72 is also found in other beta-lactamases, with Tyr72 found in the TLA beta-lactamase. Of the L2 constructs assessed in this study, the *S. maltophilia* Sm5341 L2 variant, carrying a Phe72Ile variation, was found to confer comparatively low levels of resistance to *E. coli* BL21(DE3) cells against cephalexin, ceftazidime, cefepime, and aztreonam. A lower binding affinity for cephalexin was also determined for this variant in comparison to the other L2 beta-lactamases assessed here. To further assess the importance of Phe72, two L2 mutants were generated, with results revealing a gain-of-function for the pET41a(+) based Sm5341_L2(Ile72Phe) mutant and a loss-of-function for the pET41a(+) based Sm3212_L2(Phe72Ile) mutant. These results are not unexpected, as Phe72 is found within the Ser70-Thr71-X-Lys73 active site motif. It is also present in many beta-lactamases but is not found in CTX-M enzymes which have a Ser in this position and within the VEB ESBLs which instead carry Met72. Its position within the active site would imply that it plays a role in the activity of these beta-lactamases, but its absence in CTX-M and VEB beta-lactamases could suggest that its role may be more structural especially if it forms a pi–pi stacking interaction with Phe139 in the L2 beta-lactamases. This interaction may, however, play a role in substrate recognition, binding, and catalysis, especially in L2 proteins.

Another variation observed between TOHO-1 like (clade 1) and CTX-M-97 like (clade 2) L2s is the residue Tyr272, which was found in TOHO-1 like (clade 1) L2 enzymes assessed in this study. Further analysis revealed that in addition to Tyr, position 272 was also occupied by Asn in other clade 1 L2 beta-lactamases. However, clade 2 L2 beta-lactamases were observed to carry a negatively charged residue, with Asp and less frequently Glu found in position 272. According to the RIN determined in this study, and a previous study [24], a pi–cation interaction was observed between Tyr272 and Arg244. In the study focusing on the *in-silico* identification of potential L2 inhibitors [24], Tyr272 was postulated to form a hydrophobic interaction and a hydrogen bond with ceftazidime, in addition to forming hydrogen bonds with avibactam and aztreonam. The importance of this residue within L2 was also observed in a study assessing class A beta-lactamase inhibition by relebactam, a diazabicyclooctane beta-lactamase inhibitor [31]. Here, a crystal structure of L2 complexed with relebactam identified two active-site water molecules which appeared to serve as bridges between the inhibitor and four residues, including Tyr272 and Arg244. These interactions were reportedly contributing to smaller Ki values observed for L2 as compared to CTX-M-15, which carries an Ala at this position. As such, the results observed in this study and those mentioned would suggest that Tyr272 plays a role in the activity of L2 (TOHO-1 like – clade 1) enzymes.

Also warranting further analysis is the activity of the Sm6012 L2 beta-lactamase within this isolate. The resistance profile of Sm6012 *S. maltophilia* revealed low MIC values for all second and third-generation cephalosporins and the monobactam aztreonam. In addition to these, low MICs were also observed for the carbapenems, which are known substrates of the L1 MBL [32]. However, the activity of the Sm6012 L2 beta-lactamase, when assessed in *E. coli* BL21(DE3) cells, was comparable to the Sm3212 derived L2. These results suggest that expression levels of L2 and possibly L1 may play a role in the low MICs observed for the Sm6012 isolate. As such, investigation into the regulation of these beta-lactamases is necessary to further elucidate the resistance patterns observed for this isolate.

*S. maltophilia* is recognized as an important emerging opportunistic pathogen that, due to its multidrug nature, can be difficult to treat [4]. It is also frequently recovered from polymicrobial infections, most frequently from the respiratory tract of patients suffering from cystic fibrosis [33,34]. As part of a polymicrobial infection, *S. maltophilia* can detoxify an environment by producing hydrolyzing beta-lactamases such as the L1 and L2 beta-lactamases, thereby protecting bacteria such as *P. aeruginosa*, which is also frequently isolated from patients with cystic fibrosis, complicating treatment [35]. Thus far, limited information pertains regarding the mobilization of the L1 or L2 beta-lactamases. A 2001 study reported the presence of both beta-lactamases on a 200 kb plasmid which was purified from all 10 clinical isolates assessed in this study [16]. As no whole genome sequencing was carried out as part of this study, and as *S. maltophilia* is not commonly associated with plasmids [36], it is difficult to ascertain the potential of these L1- and L2-carrying plasmids being transferred to other bacteria. Nonetheless, with the continual emergence of *S. maltophilia* in healthcare settings which are known for selecting resistant bacteria, a mobilization event may occur by allowing these beta-lactamases to be passed on to other organisms found in these environments.

This is the first study to assess variant *S. maltophilia* L2 beta-lactamases and explore their activity. Clustering into distinct clades, clade 1 currently represents a greater number of L2 beta-lactamases. This suggests the presence of a selective force that may be selecting for L2 variants able to confer *S. maltophilia* greater protection against beta-lactams such as extended-spectrum cephalosporins. With beta-lactam antibiotics being one of the most commonly prescribed drug classes [37], their overuse may continue to drive the evolution of L2 beta-lactamases leading to the development of variants with increased hydrolytic capabilities, further increasing resistance within *S. maltophilia*. This is also the first study to highlight the importance of Phe72 in the activity of L2 beta-lactamases and proposes that the active site motif include this residue and be redefined as Ser70-Thr71-Phe72-Lys73.

## Material and methods

### Bacterial strains

*S. maltophilia* was isolated as part of a project assessing the development of antimicrobial resistance in residential aged care facilities [38]. *Escherichia coli* XL10 Gold (Agilent) was used as a host for recombinant plasmid propagation. *E. coli* BL21(DE3) (Stratagene) was used as a host for T7 promoter-based L2 overexpression and antibiotic susceptibility assays. Unless otherwise specified, all *E. coli* strains were grown aerobically at 37°C in DifcoTM Luria-Bertani broth, Miller (BD) supplemented with kanamycin 25 mg/l.

### Recombinant DNA methodologies

The *bla*_L2_ genes were amplified from *S. maltophilia* chromosomal DNA using sequence-specific primers (Table 6). Chromosomal DNA was prepared using the MN NucleoSpin® Microbial DNA kit (Macherey-Nagel GmbH and Co.KG, Duren, Germany) following the manufacturer’s instructions. Amplified *bla*_L2_ genes were cloned in the pET41a(+) expression vector (Novagen, USA), yielding *bla*_L2_ -C terminally His(x8) tagged constructs.

*E. coli* strain XL10 Gold (Agilent) cells transformed with pET41a(+)/L2 recombinant plasmids and selected for using 25 mg/l kanamycin. All cloned *bla*_L2_ genes were sequenced by AGRF (Adelaide, Australia) to ensure that no mutations were introduced before being transformed into *E. coli* BL21(DE3) cells for protein expression of the C-terminally His-tagged L2 proteins and resistance analysis.

Site-directed mutagenesis was used to introduce Phe72Ile and Ile72Phe amino acids substitutions in the pET41a(+) based Sm3212_L2 and L2 Sm5341_L2 constructs, respectively. Wild-type pET41a(+) based L2 constructs were used as templates, and site-directed mutagenesis was performed using PCR with the appropriate primer sets (Table 6). The resulting PCR products were digested with *Dpn*I to remove template (methylated) plasmids and transformed into *E. coli* XL10 Gold (Agilent) cells. Mutations were confirmed by sequencing (AGRF, Adelaide, Australia), with verified pET41a(+) based constructs transformed into *E. coli* BL21(DE3) cells for further analysis.

**Table 6:** Primers used in this study.

	Name	Sequence (5' --> 3')
	Sm3212_L2_For	ATTATTCATATGCTCGCCCGTCGCCGATTC
CTX-M-97 like	Sm3212_L2_Rev	AATAATCTCGAGTCCGATCATTGCATCGGCCAGCG
	Sm3147_L2_Rev	AATAATCTCGAGTCCAATCATTGCATCGGCCAGCG
TOHO-1 like	SmA041_L2_Rev	AATAATCTCGAGTCCGATCAGTCGATCGGCGATGC
	Sm3119_L2_Rev	AATAATCTCGAGTCCGATCAACCGGTCGGCAATCC
Mutagenesis primers	5341_L2_Ile72Phe_For	CCGATGTGCAGCACC**TTC**AAGTCGCTGCTGGTTGCC
	3212_L2_Phe72Ile_For	CCGATGTGCAGCACG**ATC**AAGTCGCTGCTGGTTGCC

^a^Restriction enzyme sites are underlined. Codon substitution produced by each primer is indicated in bold.

### Expression, purification, and quantification of recombinant L2 proteins

*E. coli* BL21(DE3) carrying pET41a(+)/L2 constructs were cultured at 37°C in Brain Heart Infusion (BHI) (Oxoid) broth containing 25 mg/l kanamycin until the A 600 reached 0.5–0.6, at which point 1 mM isopropyl β-D-thiogalactopyranoside (IPTG) was added to the media. Culturing continued for an additional 20 h at 25°C. Cells were then harvested by centrifugation at 5,000× g for 30 min at 4°C, resuspended in desalt buffer (50 mM Tris, 200 mM NaCl, 1 0% w/v Glycerol, pH 7.2), and supplemented with 1 μg/ml DNase I (Sigma-Aldrich) and 1× cOmplete™, EDTA-free Protease Inhibitor Cocktail tablet (Roche). Following this, cells were lysed at 35 kpsi using the Constant System cell disrupter (Constant System Ltd) with insoluble material removed by ultracentrifugation at 200,000× g for 50 min at 4°C.

The recombinant His(×8)-tagged proteins were purified by immobilized metal affinity chromatography (IMAC) using the ÄKTA (Cytiva) protein purification system. The supernatant was loaded onto the 1 ml HisTrap™ (Cytiva) column. Loosely bound and unbound proteins were removed by washing with five column volumes of buffer (50 mM Tris, 200 mM NaCl, 10 mM imidazole, 10% w/v Glycerol, pH 7.6), following which the proteins were eluted by a stepwise imidazole gradient of 200, 300, and 500 mM in the same buffer. Fractions containing purified protein were then pooled and buffer was exchanged into desalt buffer using HiPrepTM 26/10 desalting column (Cytiva) to remove the imidazole. Protein purity was confirmed by SDS-PAGE on a 15% gel and purified protein concentration was determined using the DC protein assay (Bio Rad).

Protein quantification was determined by measuring the absorbance of purified protein at 280 nm using a Cytation5® (Bio-Tek®, Winooski, USA) plate reader. Light pathlength was determined using the equation recommended by BioTek (A977 – A900 sample OD / 0.18 OD = sample path length (in cm)). Protein concentration was calculated based on the Beer–Lambert law using the equation below:


c=A/εL

where A is the absorbance of the sample; ε the molar extinction coefficient or molar absorptivity of the protein (M^–1^ cm^–1^); c the concentration of the protein (molar units, M); and L the light pathlength (cm).

### Antimicrobial susceptibility assays

Minimum inhibitory concentrations (MICs) of the cephalosporins (cephalexin, cefazolin, cefoxitin, ceftazidime, ceftriaxone, and cefepime), the monobactam aztreonam, and carbapenems (meropenem and imipenem) were determined in *S. maltophilia* isolates and *E. coli* BL21(DE3) cells carrying different pET41a(+) based L2 constructs using the microdilution broth assay, as recommended by the European Committee on Antimicrobial Susceptibility Testing [39]. L2 pET41a(+) based constructs expressed in *E. coli* BL21(DE3) cells were grown at 37°C to an OD_600_ 0.4–0.6 in the presence of 25 mg/l kanamycin before 1 mM IPTG was added. This was followed by further growth of 2 h at 37°C before each bacterial culture was diluted and added to a 96-well microtiter plate containing serially diluted compound assessed in this study. All MIC assays were carried out in biological triplicates.

Aztreonam, imipenem, and meropenem were purchased from Glentham Life Sciences (Australia), whilst cephalexin, cefazolin, cefoxitin, ceftazidime, ceftriaxone, and cefepime were purchased from Sigma-Aldrich (Australia).

### Determination of kinetic parameters

The enzymatic activity of the L2 beta-lactase enzymes assessed in this study was assayed by spectrophotometrically monitoring the hydrolysis of cephalexin, ceftazidime at 260 nm, and cefepime at 250 nm using UV transparent 96-well plates (Greiner UV-Star®). Steady-state kinetic assays were carried out in triplicate at room temperature (25 ± 2°C) in 50 mM HEPES (pH 7.0) using a Cytation5® (Bio-Tek®, Winooski, USA) plate reader. A standard curve (concentration vs. absorbance) was plotted for each antibiotic and was used to calculate the substrate concentration. *K*_m_ and *k*_cat_ values were determined under initial rate conditions using the non-linear regression curve analysis performed using GraphPad Prism v.10.1.

### Bioinformatic analysis

Multiple sequence alignments of L2 protein sequences were carried out using ClustalW [14] and viewed using ESPript v3.0 [15]. Maximum likelihood phylogenetic trees were constructed with ClustalW [14] using PhyML v3.0 [40], with 100 bootstrap confidence. iTOL (interactive Tree of Life) v6.0 [17] was used to view and annotate all trees. Phylogenetic trees included established L2 protein reference sequences [10,16] and to further investigate L2 clades, 600 *S*. *maltophilia* L2 amino acid sequences were downloaded from the NCBI database and their clustering within clades was assessed.

L2 protein variants were determined by performing a BLASTp [41] search. Resulting sequences were compared and unique substitutions were identified. To assess homology and compare and determine percent identify of L2 protein sequences assessed in this study and L2 reference variants (accession no.: L2a P96465, L2b AJ251816, L2c AJ251817, and L2d AJ272110) [10,16], L2 protein sequences were aligned to the L2b reference variant sequence using BLASTp [41].

A RIN was generated to determine and assess residue contacts within the L2 beta-lactamase and was constructed using a solved L2 structure (PDB: 1N4O). RINs were generated using the RING v3.0 server [42]. PyMol [43] was used to view the structure of L2 (1N4O) and further assess residue interactions. Protein sequence logos were generated at the host server of the University of California at Berkeley (http://weblogo.berkeley.edu/logo.cgi).

## Supplementary material

Supplementary Figures and Tables

Supplementary materials

## Data Availability

All supporting data are included within the main article and its supplementary files. Kinetic data can be found in data sheet 1

## Abbreviations

BHI: Brain Heart Infusion
ESBLs: Extended-Spectrum-Beta-Lactamases
IMAC: immobilized metal affinity chromatography
IPTG: isopropyl β-D-thiogalactopyranoside
MBL: Metallo-beta-lactamase
MICs: Minimum inhibitory concentrations
NCBI: National Center for Biotechnology Information
RIN: Residue interaction network
SNPs: Single Nucleotide Polymorphisms
