# Supplementary material for: The continued evolution of the L2 cephalosporinase in Stenotrophomonas maltophilia: a key driver of beta-lactam resistance
Source: Biochem J. 2025 Jan 30;482(3):119–33. doi: 10.1042/BCJ20240478 (PMC12203928; doi:10.1042/BCJ20240478)
Supplement: Supplementary Figures and Tables [file BCJ-482-03-BCJ20240478-s001.docx]

**
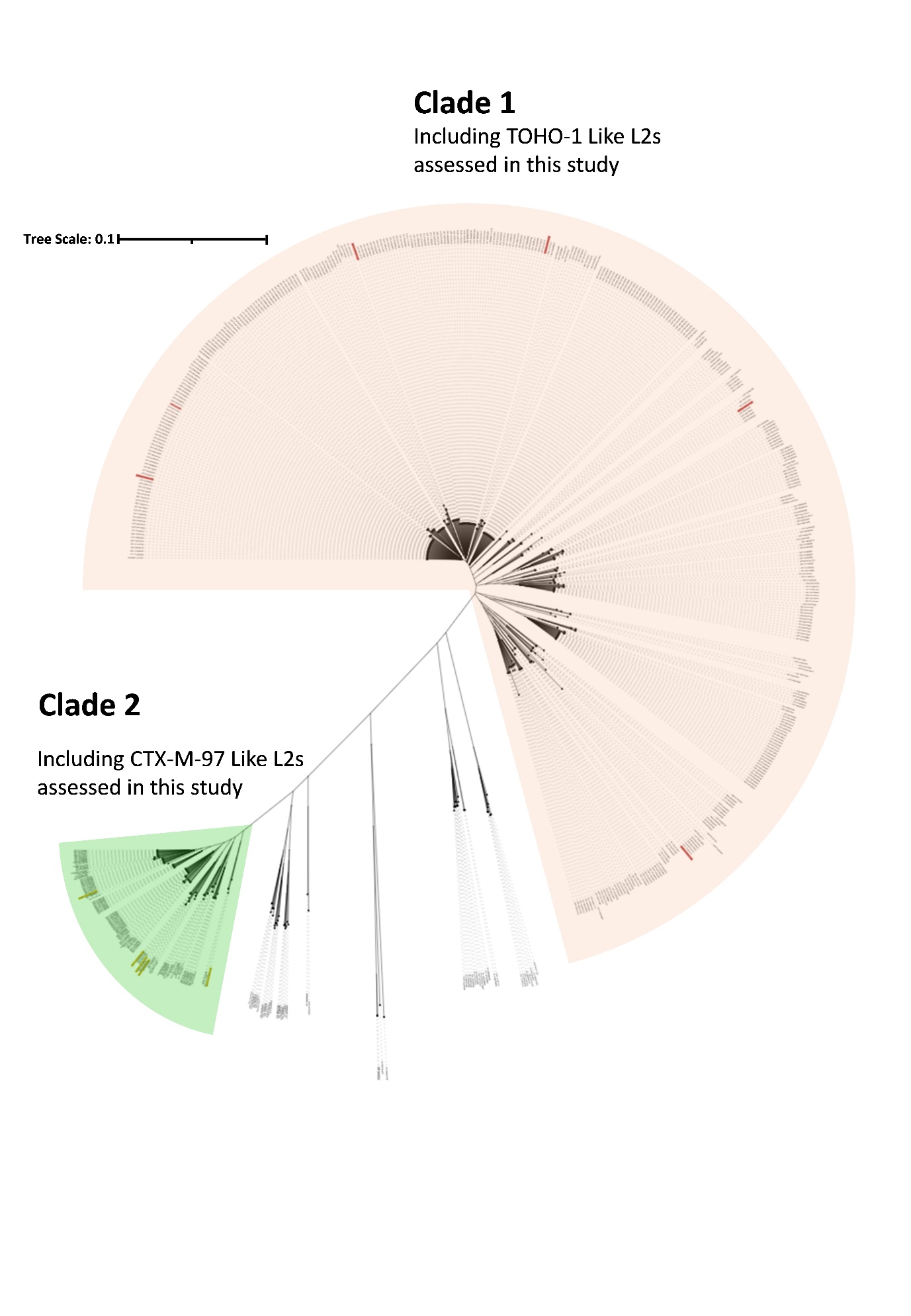
**

**Figure S1:** A maximum likelihood phylogenetic tree displaying the majority of the L2 beta-lactamases clustering in Clade 1 (orange). A multiple sequence alignment of 611 L2 beta-lactamase protein sequences was carried out using ClustalW (36), with PhyML(38) used to construct the tree, which was viewed and annotated in iTOL v6.0 (39).


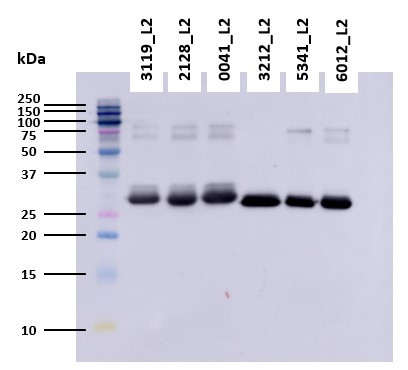


**Figure S2:** Western blot analysis of purified L2 proteins. Equal amounts of purified protein samples (5 µg) were loaded onto a 15% SDS-PAGE gel and transferred onto a PVDF membrane. The membrane was incubated with a HIS tag monoclonal mouse antibody followed by incubation with the Invitrogen™ Goat anti-Mouse Immunoglobulin G secondary antibody for protein detection. Protein bands were visualized using the enhanced chemiluminescence (ECL) detection system. Molecular weight markers are shown on the left for reference.

**Table S1:** RIN results displaying nodes and interactions between these within the L2 beta-lactamase (PDB:1N4O).

| **NodeId1** | **Interaction** | **NodeId2** | **Distance** | **Angle** | **Energy** | **Atom1** | **Atom2** | **Donor** | **Positive** | **Cation** | **Orientation** | **Model** |
| --- | --- | --- | --- | --- | --- | --- | --- | --- | --- | --- | --- | --- |
| A:23:_:THR | HBOND:MC_MC | A:26:_:ALA | 3.215 | 39.408 | 17 | O | N | A:26:_:ALA |  |  |  | 1 |
| A:23:_:THR | HBOND:MC_MC | A:27:_:ILE | 2.92 | 24.672 | 17 | O | N | A:27:_:ILE |  |  |  | 1 |
| A:23:_:THR | VDW:SC_SC | A:270:_:ILE | 3.809 | NaN | 6 | CG2 | CG2 |  |  |  |  | 1 |
| A:24:_:ASP | HBOND:MC_MC | A:27:_:ILE | 3.323 | 39.783 | 17 | O | N | A:27:_:ILE |  |  |  | 1 |
| A:24:_:ASP | HBOND:MC_MC | A:28:_:THR | 3.049 | 19.836 | 17 | O | N | A:28:_:THR |  |  |  | 1 |
| A:25:_:ALA | HBOND:MC_MC | A:28:_:THR | 3.291 | 47.588 | 17 | O | N | A:28:_:THR |  |  |  | 1 |
| A:25:_:ALA | HBOND:MC_MC | A:29:_:ALA | 2.911 | 41.522 | 17 | O | N | A:29:_:ALA |  |  |  | 1 |
| A:26:_:ALA | HBOND:MC_MC | A:29:_:ALA | 3.117 | 28.41 | 17 | O | N | A:29:_:ALA |  |  |  | 1 |
| A:26:_:ALA | HBOND:MC_MC | A:30:_:ALA | 3.06 | 58.296 | 17 | O | N | A:30:_:ALA |  |  |  | 1 |
| A:26:_:ALA | VDW:SC_SC | A:39:_:ALA | 3.652 | NaN | 6 | CB | CB |  |  |  |  | 1 |
| A:27:_:ILE | HBOND:MC_MC | A:30:_:ALA | 3.229 | 10.519 | 17 | O | N | A:30:_:ALA |  |  |  | 1 |
| A:27:_:ILE | VDW:SC_SC | A:36:_:LEU | 3.833 | NaN | 6 | CG2 | CD1 |  |  |  |  | 1 |
| A:27:_:ILE | VDW:SC_SC | A:278:_:VAL | 3.703 | NaN | 6 | CD1 | CG2 |  |  |  |  | 1 |
| A:30:_:ALA | VDW:SC_MC | A:35:_:ALA | 3.721 | NaN | 6 | CB | C |  |  |  |  | 1 |
| A:30:_:ALA | VDW:SC_SC | A:36:_:LEU | 3.772 | NaN | 6 | CB | CB |  |  |  |  | 1 |
| A:32:_:ASP | HBOND:SC_MC | A:35:_:ALA | 2.868 | 29.621 | 17 | OD1 | N | A:35:_:ALA |  |  |  | 1 |
| A:32:_:ASP | HBOND:MC_MC | A:35:_:ALA | 3.141 | 36.551 | 17 | O | N | A:35:_:ALA |  |  |  | 1 |
| A:32:_:ASP | HBOND:MC_MC | A:36:_:LEU | 3.005 | 32.42 | 17 | O | N | A:36:_:LEU |  |  |  | 1 |
| A:32:_:ASP | VDW:MC_SC | A:285:_:ILE | 3.798 | NaN | 6 | C | CD1 |  |  |  |  | 1 |
| A:33:_:PHE | HBOND:MC_MC | A:36:_:LEU | 3.262 | 37.7 | 17 | O | N | A:36:_:LEU |  |  |  | 1 |
| A:33:_:PHE | HBOND:MC_MC | A:37:_:GLU | 2.931 | 28.214 | 17 | O | N | A:37:_:GLU |  |  |  | 1 |
| A:33:_:PHE | VDW:SC_SC | A:46:_:VAL | 3.735 | NaN | 6 | CZ | CG1 |  |  |  |  | 1 |
| A:33:_:PHE | PIPISTACK:SC_SC | A:60:_:HIS | 6.298 | 107.968 | 9.4 | 90.068,90.816,5.411 | 85.207,88.090,2.477 |  |  |  | N n2.26,p4.84 | 1 |
| A:33:_:PHE | VDW:SC_SC | A:60:_:HIS | 3.528 | NaN | 6 | CB | CD2 |  |  |  |  | 1 |
| A:33:_:PHE | VDW:SC_SC | A:285:_:ILE | 3.609 | NaN | 6 | CE1 | CG2 |  |  |  |  | 1 |
| A:34:_:ALA | HBOND:MC_MC | A:37:_:GLU | 3.276 | 39.969 | 17 | O | N | A:37:_:GLU |  |  |  | 1 |
| A:34:_:ALA | HBOND:MC_MC | A:38:_:LYS | 2.942 | 25.031 | 17 | O | N | A:38:_:LYS |  |  |  | 1 |
| A:35:_:ALA | HBOND:MC_MC | A:38:_:LYS | 3.355 | 42.432 | 17 | O | N | A:38:_:LYS |  |  |  | 1 |
| A:35:_:ALA | HBOND:MC_MC | A:39:_:ALA | 2.935 | 31.256 | 17 | O | N | A:39:_:ALA |  |  |  | 1 |
| A:36:_:LEU | HBOND:MC_MC | A:39:_:ALA | 3.189 | 40.142 | 17 | O | N | A:39:_:ALA |  |  |  | 1 |
| A:36:_:LEU | HBOND:MC_MC | A:40:_:CYS | 3.371 | 38.541 | 17 | O | N | A:40:_:CYS |  |  |  | 1 |
| A:36:_:LEU | VDW:SC_SC | A:278:_:VAL | 3.799 | NaN | 6 | CG | CG1 |  |  |  |  | 1 |
| A:36:_:LEU | VDW:SC_SC | A:281:_:GLN | 3.833 | NaN | 6 | CD2 | CB |  |  |  |  | 1 |
| A:36:_:LEU | VDW:SC_SC | A:282:_:VAL | 3.689 | NaN | 6 | CD2 | CG2 |  |  |  |  | 1 |
| A:37:_:GLU | HBOND:MC_MC | A:40:_:CYS | 3.335 | 23.196 | 17 | O | N | A:40:_:CYS |  |  |  | 1 |
| A:37:_:GLU | HBOND:MC_MC | A:41:_:ALA | 2.982 | 50.278 | 17 | O | N | A:41:_:ALA |  |  |  | 1 |
| A:37:_:GLU | HBOND:MC_MC | A:42:_:GLY | 3.145 | 7.313 | 17 | O | N | A:42:_:GLY |  |  |  | 1 |
| A:37:_:GLU | VDW:SC_MC | A:42:_:GLY | 3.616 | NaN | 6 | CG | C |  |  |  |  | 1 |
| A:37:_:GLU | HBOND:SC_MC | A:44:_:LEU | 2.889 | 2.212 | 17 | OE1 | N | A:44:_:LEU |  |  |  | 1 |
| A:37:_:GLU | VDW:SC_SC | A:60:_:HIS | 3.779 | NaN | 6 | CD | CE1 |  |  |  |  | 1 |
| A:37:_:GLU | IONIC:SC_SC | A:61:_:ARG | 3.733 | 97.757 | 20 | 82.123,84.649,3.547 | CZ |  | A:61:_:ARG |  |  | 1 |
| A:38:_:LYS | HBOND:MC_MC | A:41:_:ALA | 3.143 | 20.399 | 17 | O | N | A:41:_:ALA |  |  |  | 1 |
| A:40:_:CYS | VDW:SC_SC | A:270:_:ILE | 3.656 | NaN | 6 | CB | CD1 |  |  |  |  | 1 |
| A:40:_:CYS | VDW:SC_SC | A:278:_:VAL | 3.895 | NaN | 6 | CB | CG2 |  |  |  |  | 1 |
| A:43:_:ARG | VDW:SC_SC | A:61:_:ARG | 3.871 | NaN | 6 | CG | CD |  |  |  |  | 1 |
| A:43:_:ARG | PICATION:SC_SC | A:66:_:PHE | 3.55 | 9.869 | 9.6 | CZ | CE2 |  |  | A:43:_:ARG | P | 1 |
| A:43:_:ARG | VDW:SC_SC | A:66:_:PHE | 3.55 | NaN | 6 | CZ | CE2 |  |  |  |  | 1 |
| A:43:_:ARG | HBOND:MC_MC | A:266:_:GLN | 2.818 | 9.237 | 17 | N | O | A:43:_:ARG |  |  |  | 1 |
| A:43:_:ARG | HBOND:MC_MC | A:266:_:GLN | 2.896 | 5.136 | 17 | O | N | A:266:_:GLN |  |  |  | 1 |
| A:43:_:ARG | VDW:SC_SC | A:266:_:GLN | 3.532 | NaN | 6 | CZ | OE1 |  |  |  |  | 1 |
| A:44:_:LEU | VDW:MC_SC | A:66:_:PHE | 3.645 | NaN | 6 | C | CZ |  |  |  |  | 1 |
| A:45:_:GLY | VDW:MC_SC | A:183:_:PRO | 3.434 | NaN | 6 | C | CG |  |  |  |  | 1 |
| A:45:_:GLY | HBOND:MC_MC | A:264:_:TYR | 2.732 | 12.556 | 17 | N | O | A:45:_:GLY |  |  |  | 1 |
| A:45:_:GLY | HBOND:MC_MC | A:264:_:TYR | 2.754 | 6.932 | 17 | O | N | A:264:_:TYR |  |  |  | 1 |
| A:46:_:VAL | HBOND:MC_MC | A:60:_:HIS | 2.901 | 12.136 | 17 | N | O | A:46:_:VAL |  |  |  | 1 |
| A:46:_:VAL | HBOND:MC_MC | A:60:_:HIS | 2.904 | 24.947 | 17 | O | N | A:60:_:HIS |  |  |  | 1 |
| A:46:_:VAL | VDW:MC_SC | A:183:_:PRO | 3.767 | NaN | 6 | C | CB |  |  |  |  | 1 |
| A:47:_:THR | VDW:SC_SC | A:56:_:ARG | 3.869 | NaN | 6 | CG2 | CB |  |  |  |  | 1 |
| A:47:_:THR | VDW:SC_SC | A:62:_:GLN | 3.533 | NaN | 6 | CG2 | OE1 |  |  |  |  | 1 |
| A:47:_:THR | VDW:SC_MC | A:183:_:PRO | 3.857 | NaN | 6 | CB | C |  |  |  |  | 1 |
| A:47:_:THR | VDW:SC_SC | A:187:_:ALA | 3.639 | NaN | 6 | CG2 | CB |  |  |  |  | 1 |
| A:47:_:THR | HBOND:MC_MC | A:262:_:THR | 2.907 | 9.168 | 17 | O | N | A:262:_:THR |  |  |  | 1 |
| A:47:_:THR | HBOND:MC_MC | A:262:_:THR | 3.107 | 17.19 | 17 | N | O | A:47:_:THR |  |  |  | 1 |
| A:48:_:LEU | HBOND:MC_MC | A:57:_:ILE | 2.846 | 13.433 | 17 | O | N | A:57:_:ILE |  |  |  | 1 |
| A:48:_:LEU | HBOND:MC_MC | A:57:_:ILE | 3.112 | 13.422 | 17 | N | O | A:48:_:LEU |  |  |  | 1 |
| A:48:_:LEU | VDW:SC_SC | A:259:_:TRP | 3.591 | NaN | 6 | CD1 | CE3 |  |  |  |  | 1 |
| A:49:_:LEU | VDW:SC_MC | A:54:_:GLY | 3.763 | NaN | 6 | CD1 | C |  |  |  |  | 1 |
| A:49:_:LEU | VDW:SC_SC | A:191:_:GLN | 3.46 | NaN | 6 | CD2 | OE1 |  |  |  |  | 1 |
| A:49:_:LEU | VDW:MC_SC | A:259:_:TRP | 3.624 | NaN | 6 | C | CE3 |  |  |  |  | 1 |
| A:49:_:LEU | HBOND:MC_MC | A:260:_:VAL | 2.798 | 4.551 | 17 | N | O | A:49:_:LEU |  |  |  | 1 |
| A:49:_:LEU | HBOND:MC_MC | A:260:_:VAL | 2.94 | 11.282 | 17 | O | N | A:260:_:VAL |  |  |  | 1 |
| A:50:_:ASP | HBOND:MC_MC | A:53:_:SER | 3.417 | 24.824 | 17 | O | N | A:53:_:SER |  |  |  | 1 |
| A:50:_:ASP | HBOND:MC_MC | A:54:_:GLY | 2.815 | 29.167 | 17 | O | N | A:54:_:GLY |  |  |  | 1 |
| A:50:_:ASP | HBOND:MC_MC | A:55:_:ARG | 2.895 | 6.124 | 17 | N | O | A:50:_:ASP |  |  |  | 1 |
| A:50:_:ASP | VDW:SC_SC | A:259:_:TRP | 3.633 | NaN | 6 | CB | CZ3 |  |  |  |  | 1 |
| A:50:_:ASP | VDW:SC_SC | A:259:_:TRP | 3.885 | NaN | 6 | CG | CZ2 |  |  |  |  | 1 |
| A:51:_:THR | VDW:SC_SC | A:191:_:GLN | 3.477 | NaN | 6 | CB | NE2 |  |  |  |  | 1 |
| A:51:_:THR | VDW:SC_SC | A:195:_:LEU | 3.736 | NaN | 6 | CG2 | CD1 |  |  |  |  | 1 |
| A:51:_:THR | HBOND:MC_MC | A:258:_:PRO | 3.237 | 20.527 | 17 | N | O | A:51:_:THR |  |  |  | 1 |
| A:52:_:ALA | VDW:SC_SC | A:257:_:ALA | 3.587 | NaN | 6 | CB | CB |  |  |  |  | 1 |
| A:61:_:ARG | HBOND:MC_MC | A:64:_:GLU | 3.323 | 11.987 | 17 | O | N | A:64:_:GLU |  |  |  | 1 |
| A:61:_:ARG | VDW:SC_SC | A:64:_:GLU | 3.809 | NaN | 6 | CD | CD |  |  |  |  | 1 |
| A:62:_:GLN | VDW:MC_SC | A:183:_:PRO | 3.753 | NaN | 6 | C | CD |  |  |  |  | 1 |
| A:62:_:GLN | HBOND:MC_MC | A:184:_:ALA | 2.974 | 24.737 | 17 | O | N | A:184:_:ALA |  |  |  | 1 |
| A:63:_:ASP | VDW:MC_SC | A:182:_:THR | 3.861 | NaN | 6 | C | CG2 |  |  |  |  | 1 |
| A:64:_:GLU | VDW:MC_SC | A:182:_:THR | 3.747 | NaN | 6 | C | CG2 |  |  |  |  | 1 |
| A:66:_:PHE | HBOND:MC_MC | A:181:_:THR | 2.932 | 12.197 | 17 | N | O | A:66:_:PHE |  |  |  | 1 |
| A:66:_:PHE | HBOND:MC_MC | A:181:_:THR | 3.021 | 13.702 | 17 | O | N | A:181:_:THR |  |  |  | 1 |
| A:66:_:PHE | VDW:SC_SC | A:183:_:PRO | 3.833 | NaN | 6 | CD1 | CD |  |  |  |  | 1 |
| A:66:_:PHE | PIPISTACK:SC_SC | A:264:_:TYR | 6.048 | 145.613 | 9.4 | 77.824,91.673,6.224 | 79.173,96.178,10.026 |  |  |  | L n4.53,p3.66 | 1 |
| A:66:_:PHE | VDW:SC_SC | A:264:_:TYR | 3.473 | NaN | 6 | CG | CB |  |  |  |  | 1 |
| A:67:_:PRO | VDW:SC_SC | A:172:_:PHE | 3.492 | NaN | 6 | CD | CD1 |  |  |  |  | 1 |
| A:67:_:PRO | VDW:SC_SC | A:172:_:PHE | 3.78 | NaN | 6 | CB | CB |  |  |  |  | 1 |
| A:68:_:MET | HBOND:MC_MC | A:71:_:THR | 3.066 | 13.117 | 17 | O | N | A:71:_:THR |  |  |  | 1 |
| A:68:_:MET | VDW:SC_SC | A:72:_:PHE | 3.729 | NaN | 6 | SD | CB |  |  |  |  | 1 |
| A:68:_:MET | VDW:SC_MC | A:161:_:ARG | 3.864 | NaN | 6 | CE | C |  |  |  |  | 1 |
| A:68:_:MET | HBOND:MC_MC | A:179:_:ASP | 2.919 | 14.598 | 17 | N | O | A:68:_:MET |  |  |  | 1 |
| A:68:_:MET | VDW:SC_MC | A:179:_:ASP | 3.775 | NaN | 6 | CE | C |  |  |  |  | 1 |
| A:68:_:MET | VDW:SC_MC | A:180:_:THR | 3.881 | NaN | 6 | CG | C |  |  |  |  | 1 |
| A:68:_:MET | VDW:SC_SC | A:181:_:THR | 3.574 | NaN | 6 | CG | CG2 |  |  |  |  | 1 |
| A:68:_:MET | VDW:SC_SC | A:186:_:MET | 3.751 | NaN | 6 | SD | CE |  |  |  |  | 1 |
| A:68:_:MET | VDW:MC_SC | A:245:_:ASN | 3.667 | NaN | 6 | C | ND2 |  |  |  |  | 1 |
| A:69:_:CYS | HBOND:MC_MC | A:72:_:PHE | 3.261 | 13.32 | 17 | O | N | A:72:_:PHE |  |  |  | 1 |
| A:69:_:CYS | VDW:SC_SC | A:170:_:ASN | 3.431 | NaN | 6 | CB | ND2 |  |  |  |  | 1 |
| A:69:_:CYS | VDW:SC_MC | A:237:_:SER | 3.686 | NaN | 6 | SG | C |  |  |  |  | 1 |
| A:69:_:CYS | VDW:SC_SC | A:238:_:ASN | 3.959 | NaN | 6 | SG | CB |  |  |  |  | 1 |
| A:70:_:SER | HBOND:MC_MC | A:73:_:LYS | 2.934 | 12.661 | 17 | O | N | A:73:_:LYS |  |  |  | 1 |
| A:70:_:SER | VDW:SC_SC | A:73:_:LYS | 3.838 | NaN | 6 | CB | CD |  |  |  |  | 1 |
| A:71:_:THR | HBOND:MC_MC | A:74:_:SER | 3.323 | 12.937 | 17 | O | N | A:74:_:SER |  |  |  | 1 |
| A:71:_:THR | VDW:SC_MC | A:235:_:THR | 3.847 | NaN | 6 | CG2 | C |  |  |  |  | 1 |
| A:71:_:THR | VDW:SC_SC | A:245:_:ASN | 3.721 | NaN | 6 | CG2 | CB |  |  |  |  | 1 |
| A:71:_:THR | VDW:SC_SC | A:247:_:ILE | 3.799 | NaN | 6 | CB | CD1 |  |  |  |  | 1 |
| A:72:_:PHE | HBOND:MC_MC | A:75:_:MET | 2.862 | 28.852 | 17 | O | N | A:75:_:MET |  |  |  | 1 |
| A:72:_:PHE | HBOND:MC_MC | A:76:_:LEU | 2.994 | 32.027 | 17 | O | N | A:76:_:LEU |  |  |  | 1 |
| A:72:_:PHE | PIPISTACK:SC_SC | A:139:_:PHE | 5.37 | 12.488 | 9.4 | 71.943,102.659,16.969 | 67.714,105.839,17.883 |  |  |  | P n2.47,p4.12 | 1 |
| A:72:_:PHE | VDW:SC_SC | A:139:_:PHE | 3.308 | NaN | 6 | CE2 | CZ |  |  |  |  | 1 |
| A:72:_:PHE | VDW:SC_SC | A:139:_:PHE | 3.652 | NaN | 6 | CZ | CE2 |  |  |  |  | 1 |
| A:72:_:PHE | VDW:SC_SC | A:169:_:LEU | 3.392 | NaN | 6 | CE1 | CD2 |  |  |  |  | 1 |
| A:73:_:LYS | HBOND:MC_MC | A:76:_:LEU | 3.261 | 38.123 | 17 | O | N | A:76:_:LEU |  |  |  | 1 |
| A:73:_:LYS | HBOND:MC_MC | A:77:_:ALA | 2.894 | 25.939 | 17 | O | N | A:77:_:ALA |  |  |  | 1 |
| A:73:_:LYS | VDW:MC_SC | A:127:_:ILE | 3.63 | NaN | 6 | C | CD1 |  |  |  |  | 1 |
| A:73:_:LYS | VDW:SC_SC | A:132:_:ASN | 3.577 | NaN | 6 | CE | OD1 |  |  |  |  | 1 |
| A:73:_:LYS | VDW:SC_SC | A:135:_:ALA | 3.733 | NaN | 6 | CE | CB |  |  |  |  | 1 |
| A:73:_:LYS | VDW:SC_SC | A:234:_:LYS | 3.8 | NaN | 6 | CB | CE |  |  |  |  | 1 |
| A:74:_:SER | HBOND:MC_MC | A:78:_:ALA | 3.04 | 35.745 | 17 | O | N | A:78:_:ALA |  |  |  | 1 |
| A:75:_:MET | HBOND:MC_MC | A:78:_:ALA | 3.349 | 43.612 | 17 | O | N | A:78:_:ALA |  |  |  | 1 |
| A:75:_:MET | HBOND:MC_MC | A:79:_:THR | 2.888 | 28.749 | 17 | O | N | A:79:_:THR |  |  |  | 1 |
| A:75:_:MET | VDW:SC_SC | A:189:_:THR | 3.598 | NaN | 6 | SD | CG2 |  |  |  |  | 1 |
| A:75:_:MET | VDW:SC_SC | A:190:_:LEU | 3.843 | NaN | 6 | CE | CD2 |  |  |  |  | 1 |
| A:76:_:LEU | HBOND:MC_MC | A:79:_:THR | 3.149 | 40.106 | 17 | O | N | A:79:_:THR |  |  |  | 1 |
| A:76:_:LEU | HBOND:MC_MC | A:80:_:VAL | 3.073 | 23.108 | 17 | O | N | A:80:_:VAL |  |  |  | 1 |
| A:76:_:LEU | VDW:SC_SC | A:138:_:LEU | 3.896 | NaN | 6 | CD2 | CB |  |  |  |  | 1 |
| A:76:_:LEU | VDW:SC_SC | A:148:_:VAL | 3.728 | NaN | 6 | CD1 | CG2 |  |  |  |  | 1 |
| A:77:_:ALA | HBOND:MC_MC | A:80:_:VAL | 3.216 | 42.509 | 17 | O | N | A:80:_:VAL |  |  |  | 1 |
| A:77:_:ALA | HBOND:MC_MC | A:81:_:LEU | 2.965 | 20.352 | 17 | O | N | A:81:_:LEU |  |  |  | 1 |
| A:78:_:ALA | HBOND:MC_MC | A:81:_:LEU | 3.296 | 47.546 | 17 | O | N | A:81:_:LEU |  |  |  | 1 |
| A:78:_:ALA | HBOND:MC_MC | A:82:_:SER | 2.833 | 30.122 | 17 | O | N | A:82:_:SER |  |  |  | 1 |
| A:78:_:ALA | VDW:SC_SC | A:151:_:PHE | 3.614 | NaN | 6 | CB | CZ |  |  |  |  | 1 |
| A:78:_:ALA | VDW:SC_SC | A:193:_:VAL | 3.632 | NaN | 6 | CB | CG1 |  |  |  |  | 1 |
| A:78:_:ALA | VDW:SC_SC | A:207:_:LEU | 3.877 | NaN | 6 | CB | CD2 |  |  |  |  | 1 |
| A:79:_:THR | HBOND:MC_MC | A:82:_:SER | 3.306 | 38.589 | 17 | O | N | A:82:_:SER |  |  |  | 1 |
| A:79:_:THR | HBOND:MC_MC | A:83:_:GLN | 2.954 | 19.617 | 17 | O | N | A:83:_:GLN |  |  |  | 1 |
| A:80:_:VAL | HBOND:MC_MC | A:83:_:GLN | 3.241 | 44.461 | 17 | O | N | A:83:_:GLN |  |  |  | 1 |
| A:80:_:VAL | HBOND:MC_MC | A:84:_:ALA | 2.888 | 34.65 | 17 | O | N | A:84:_:ALA |  |  |  | 1 |
| A:80:_:VAL | VDW:SC_SC | A:91:_:LEU | 3.818 | NaN | 6 | CG1 | CD2 |  |  |  |  | 1 |
| A:80:_:VAL | VDW:SC_SC | A:119:_:VAL | 3.588 | NaN | 6 | CG1 | CG1 |  |  |  |  | 1 |
| A:80:_:VAL | VDW:SC_SC | A:123:_:CYS | 3.657 | NaN | 6 | CG1 | SG |  |  |  |  | 1 |
| A:80:_:VAL | VDW:SC_SC | A:142:_:VAL | 3.365 | NaN | 6 | CG2 | CG1 |  |  |  |  | 1 |
| A:81:_:LEU | HBOND:MC_MC | A:84:_:ALA | 3.19 | 34.385 | 17 | O | N | A:84:_:ALA |  |  |  | 1 |
| A:81:_:LEU | HBOND:MC_MC | A:85:_:GLU | 3.253 | 51.43 | 17 | O | N | A:85:_:GLU |  |  |  | 1 |
| A:81:_:LEU | VDW:SC_SC | A:207:_:LEU | 3.719 | NaN | 6 | CD1 | CB |  |  |  |  | 1 |
| A:81:_:LEU | VDW:SC_SC | A:210:_:TRP | 3.778 | NaN | 6 | CD1 | CZ3 |  |  |  |  | 1 |
| A:82:_:SER | HBOND:MC_MC | A:85:_:GLU | 3.104 | 15.248 | 17 | O | N | A:85:_:GLU |  |  |  | 1 |
| A:82:_:SER | VDW:SC_SC | A:151:_:PHE | 3.759 | NaN | 6 | CB | CE1 |  |  |  |  | 1 |
| A:82:_:SER | VDW:SC_SC | A:199:_:LEU | 3.694 | NaN | 6 | CB | CD2 |  |  |  |  | 1 |
| A:83:_:GLN | HBOND:MC_MC | A:86:_:ARG | 3.081 | 12.35 | 17 | O | N | A:86:_:ARG |  |  |  | 1 |
| A:83:_:GLN | HBOND:MC_MC | A:87:_:MET | 3.266 | 54.002 | 17 | O | N | A:87:_:MET |  |  |  | 1 |
| A:83:_:GLN | VDW:SC_SC | A:90:_:LEU | 3.814 | NaN | 6 | CB | CD2 |  |  |  |  | 1 |
| A:83:_:GLN | VDW:SC_SC | A:142:_:VAL | 3.684 | NaN | 6 | OE1 | CB |  |  |  |  | 1 |
| A:84:_:ALA | HBOND:MC_MC | A:87:_:MET | 3.081 | 19.586 | 17 | O | N | A:87:_:MET |  |  |  | 1 |
| A:84:_:ALA | VDW:SC_SC | A:91:_:LEU | 3.705 | NaN | 6 | CB | CG |  |  |  |  | 1 |
| A:85:_:GLU | HBOND:SC_MC | A:200:_:GLN | 2.81 | 4.193 | 17 | OE2 | N | A:200:_:GLN |  |  |  | 1 |
| A:85:_:GLU | VDW:SC_SC | A:200:_:GLN | 3.481 | NaN | 6 | CG | NE2 |  |  |  |  | 1 |
| A:85:_:GLU | VDW:SC_SC | A:200:_:GLN | 3.557 | NaN | 6 | CD | CG |  |  |  |  | 1 |
| A:87:_:MET | HBOND:MC_MC | A:90:_:LEU | 2.995 | 18.978 | 17 | O | N | A:90:_:LEU |  |  |  | 1 |
| A:89:_:ALA | HBOND:MC_MC | A:92:_:ASP | 3.285 | 27.209 | 17 | O | N | A:92:_:ASP |  |  |  | 1 |
| A:89:_:ALA | HBOND:MC_MC | A:93:_:ARG | 3.17 | 59.156 | 17 | O | N | A:93:_:ARG |  |  |  | 1 |
| A:90:_:LEU | HBOND:MC_MC | A:93:_:ARG | 3.121 | 11.582 | 17 | O | N | A:93:_:ARG |  |  |  | 1 |
| A:90:_:LEU | VDW:SC_SC | A:141:_:VAL | 3.668 | NaN | 6 | CD1 | CG1 |  |  |  |  | 1 |
| A:91:_:LEU | HBOND:MC_MC | A:120:_:ARG | 2.953 | 29.323 | 17 | O | N | A:120:_:ARG |  |  |  | 1 |
| A:91:_:LEU | VDW:SC_SC | A:120:_:ARG | 3.726 | NaN | 6 | CB | CZ |  |  |  |  | 1 |
| A:93:_:ARG | HBOND:MC_MC | A:119:_:VAL | 2.846 | 18.952 | 17 | O | N | A:119:_:VAL |  |  |  | 1 |
| A:94:_:ARG | VDW:SC_SC | A:116:_:ASP | 3.641 | NaN | 6 | CB | CG |  |  |  |  | 1 |
| A:94:_:ARG | VDW:SC_SC | A:118:_:THR | 3.835 | NaN | 6 | CZ | CG2 |  |  |  |  | 1 |
| A:94:_:ARG | VDW:SC_SC | B:205:_:GLN | 3.578 | NaN | 6 | CZ | CB |  |  |  |  | 1 |
| A:94:_:ARG | VDW:SC_SC | B:205:_:GLN | 3.609 | NaN | 6 | CD | NE2 |  |  |  |  | 1 |
| A:94:_:ARG | IONIC:SC_SC | B:209:_:ASP | 3.957 | 155.645 | 20 | CZ | 74.804,112.986,37.218 |  | A:94:_:ARG |  |  | 1 |
| A:95:_:VAL | VDW:MC_SC | A:116:_:ASP | 3.537 | NaN | 6 | C | CB |  |  |  |  | 1 |
| A:95:_:VAL | HBOND:MC_MC | A:117:_:MET | 2.914 | 10.835 | 17 | N | O | A:95:_:VAL |  |  |  | 1 |
| A:95:_:VAL | HBOND:MC_MC | A:117:_:MET | 3.08 | 16.556 | 17 | O | N | A:117:_:MET |  |  |  | 1 |
| A:95:_:VAL | VDW:SC_SC | A:141:_:VAL | 3.709 | NaN | 6 | CG2 | CG2 |  |  |  |  | 1 |
| A:97:_:VAL | HBOND:MC_MC | A:114:_:GLY | 2.788 | 24.311 | 17 | O | N | A:114:_:GLY |  |  |  | 1 |
| A:97:_:VAL | HBOND:MC_MC | A:115:_:LYS | 2.913 | 3.92 | 17 | N | O | A:97:_:VAL |  |  |  | 1 |
| A:97:_:VAL | VDW:SC_SC | A:137:_:LEU | 3.882 | NaN | 6 | CG2 | CD1 |  |  |  |  | 1 |
| A:98:_:GLY | HBOND:MC_MC | A:101:_:ASP | 2.939 | 11.153 | 17 | O | N | A:101:_:ASP |  |  |  | 1 |
| A:99:_:GLU | HBOND:MC_MC | A:102:_:LEU | 3.359 | 17.152 | 17 | O | N | A:102:_:LEU |  |  |  | 1 |
| A:99:_:GLU | VDW:SC_SC | A:102:_:LEU | 3.746 | NaN | 6 | CD | CD1 |  |  |  |  | 1 |
| A:103:_:LEU | VDW:SC_SC | A:132:_:ASN | 3.888 | NaN | 6 | CD1 | CB |  |  |  |  | 1 |
| A:103:_:LEU | HBOND:MC_SC | A:133:_:THR | 2.989 | 26.068 | 17 | N | OG1 | A:103:_:LEU |  |  |  | 1 |
| A:103:_:LEU | VDW:SC_SC | A:136:_:ASN | 3.713 | NaN | 6 | CD1 | ND2 |  |  |  |  | 1 |
| A:105:_:HIS | HBOND:MC_MC | A:132:_:ASN | 2.878 | 17.713 | 17 | O | N | A:132:_:ASN |  |  |  | 1 |
| A:105:_:HIS | VDW:SC_SC | A:132:_:ASN | 3.702 | NaN | 6 | CB | ND2 |  |  |  |  | 1 |
| A:106:_:ALA | HBOND:MC_MC | A:110:_:ARG | 3.063 | 34.068 | 17 | O | N | A:110:_:ARG |  |  |  | 1 |
| A:106:_:ALA | VDW:MC_SC | A:131:_:ASP | 3.807 | NaN | 6 | C | CG |  |  |  |  | 1 |
| A:107:_:PRO | VDW:SC_SC | A:129:_:THR | 3.655 | NaN | 6 | CG | CB |  |  |  |  | 1 |
| A:108:_:VAL | HBOND:MC_MC | A:111:_:ARG | 3.133 | 10.499 | 17 | O | N | A:111:_:ARG |  |  |  | 1 |
| A:108:_:VAL | VDW:SC_SC | A:125:_:ALA | 3.659 | NaN | 6 | CB | CB |  |  |  |  | 1 |
| A:108:_:VAL | VDW:SC_SC | A:129:_:THR | 3.774 | NaN | 6 | CG2 | CG2 |  |  |  |  | 1 |
| A:108:_:VAL | HBOND:MC_SC | A:131:_:ASP | 2.895 | 17.882 | 17 | N | OD2 | A:108:_:VAL |  |  |  | 1 |
| A:109:_:THR | HBOND:MC_MC | A:112:_:HIS | 2.82 | 16.18 | 17 | O | N | A:112:_:HIS |  |  |  | 1 |
| A:109:_:THR | VDW:SC_SC | A:117:_:MET | 3.834 | NaN | 6 | CG2 | SD |  |  |  |  | 1 |
| A:109:_:THR | VDW:SC_SC | A:122:_:LEU | 3.799 | NaN | 6 | CG2 | CD2 |  |  |  |  | 1 |
| A:109:_:THR | HBOND:MC_SC | A:131:_:ASP | 2.847 | 14.086 | 17 | N | OD2 | A:109:_:THR |  |  |  | 1 |
| A:109:_:THR | VDW:SC_SC | A:131:_:ASP | 3.844 | NaN | 6 | CG2 | CG |  |  |  |  | 1 |
| A:109:_:THR | VDW:SC_SC | A:134:_:ALA | 3.852 | NaN | 6 | CG2 | CB |  |  |  |  | 1 |
| A:111:_:ARG | VDW:SC_SC | B:212:_:ILE | 3.878 | NaN | 6 | CB | CG2 |  |  |  |  | 1 |
| A:112:_:HIS | HBOND:MC_MC | A:115:_:LYS | 2.941 | 26.838 | 17 | O | N | A:115:_:LYS |  |  |  | 1 |
| A:112:_:HIS | VDW:MC_SC | A:115:_:LYS | 3.617 | NaN | 6 | C | CG |  |  |  |  | 1 |
| A:112:_:HIS | VDW:SC_SC | A:117:_:MET | 3.703 | NaN | 6 | CG | CG |  |  |  |  | 1 |
| A:112:_:HIS | VDW:SC_SC | A:117:_:MET | 3.705 | NaN | 6 | CD2 | SD |  |  |  |  | 1 |
| A:112:_:HIS | VDW:SC_SC | B:209:_:ASP | 3.857 | NaN | 6 | CE1 | CG |  |  |  |  | 1 |
| A:112:_:HIS | VDW:SC_SC | B:212:_:ILE | 3.585 | NaN | 6 | CE1 | CG2 |  |  |  |  | 1 |
| A:115:_:LYS | IONIC:SC_SC | B:209:_:ASP | 3.26 | 137.418 | 20 | NZ | 74.804,112.986,37.218 |  | A:115:_:LYS |  |  | 1 |
| A:115:_:LYS | VDW:SC_SC | B:212:_:ILE | 3.662 | NaN | 6 | CE | CD1 |  |  |  |  | 1 |
| A:115:_:LYS | VDW:SC_SC | B:230:_:ARG | 3.669 | NaN | 6 | CD | CZ |  |  |  |  | 1 |
| A:117:_:MET | VDW:SC_SC | A:122:_:LEU | 3.824 | NaN | 6 | CE | CD2 |  |  |  |  | 1 |
| A:118:_:THR | HBOND:SC_MC | A:121:_:ASP | 2.94 | 31.928 | 17 | OG1 | N | A:121:_:ASP |  |  |  | 1 |
| A:118:_:THR | HBOND:MC_MC | A:121:_:ASP | 3.078 | 33.286 | 17 | O | N | A:121:_:ASP |  |  |  | 1 |
| A:118:_:THR | HBOND:MC_MC | A:122:_:LEU | 2.863 | 29.272 | 17 | O | N | A:122:_:LEU |  |  |  | 1 |
| A:119:_:VAL | HBOND:MC_MC | A:122:_:LEU | 3.164 | 38.74 | 17 | O | N | A:122:_:LEU |  |  |  | 1 |
| A:119:_:VAL | HBOND:MC_MC | A:123:_:CYS | 2.928 | 24.633 | 17 | O | N | A:123:_:CYS |  |  |  | 1 |
| A:120:_:ARG | HBOND:MC_MC | A:123:_:CYS | 3.237 | 42.171 | 17 | O | N | A:123:_:CYS |  |  |  | 1 |
| A:120:_:ARG | HBOND:MC_MC | A:124:_:ARG | 3.045 | 27.289 | 17 | O | N | A:124:_:ARG |  |  |  | 1 |
| A:120:_:ARG | VDW:SC_SC | B:120:_:ARG | 3.333 | NaN | 6 | CZ | CZ |  |  |  |  | 1 |
| A:120:_:ARG | VDW:SC_SC | B:120:_:ARG | 3.523 | NaN | 6 | CD | CD |  |  |  |  | 1 |
| A:121:_:ASP | HBOND:MC_MC | A:124:_:ARG | 3.421 | 42 | 17 | O | N | A:124:_:ARG |  |  |  | 1 |
| A:121:_:ASP | HBOND:MC_MC | A:125:_:ALA | 2.963 | 25.567 | 17 | O | N | A:125:_:ALA |  |  |  | 1 |
| A:122:_:LEU | HBOND:MC_MC | A:125:_:ALA | 3.335 | 43.231 | 17 | O | N | A:125:_:ALA |  |  |  | 1 |
| A:122:_:LEU | HBOND:MC_MC | A:126:_:THR | 2.976 | 37.323 | 17 | O | N | A:126:_:THR |  |  |  | 1 |
| A:122:_:LEU | VDW:SC_SC | A:134:_:ALA | 3.812 | NaN | 6 | CD2 | CB |  |  |  |  | 1 |
| A:122:_:LEU | VDW:MC_SC | A:138:_:LEU | 3.715 | NaN | 6 | C | CD1 |  |  |  |  | 1 |
| A:122:_:LEU | VDW:SC_SC | A:138:_:LEU | 3.836 | NaN | 6 | CB | CD2 |  |  |  |  | 1 |
| A:123:_:CYS | HBOND:MC_MC | A:126:_:THR | 3.368 | 27.942 | 17 | O | N | A:126:_:THR |  |  |  | 1 |
| A:123:_:CYS | HBOND:MC_MC | A:127:_:ILE | 3.196 | 31.326 | 17 | O | N | A:127:_:ILE |  |  |  | 1 |
| A:123:_:CYS | VDW:SC_SC | A:138:_:LEU | 3.999 | NaN | 6 | SG | CD2 |  |  |  |  | 1 |
| A:123:_:CYS | VDW:SC_SC | A:210:_:TRP | 3.493 | NaN | 6 | CB | CZ3 |  |  |  |  | 1 |
| A:124:_:ARG | HBOND:MC_MC | A:127:_:ILE | 3.174 | 31.256 | 17 | O | N | A:127:_:ILE |  |  |  | 1 |
| A:124:_:ARG | HBOND:MC_MC | A:128:_:ILE | 2.89 | 15.805 | 17 | O | N | A:128:_:ILE |  |  |  | 1 |
| A:124:_:ARG | VDW:SC_SC | B:124:_:ARG | 3.596 | NaN | 6 | CD | CD |  |  |  |  | 1 |
| A:125:_:ALA | HBOND:MC_MC | A:129:_:THR | 3.077 | 26.172 | 17 | O | N | A:129:_:THR |  |  |  | 1 |
| A:125:_:ALA | HBOND:MC_MC | A:130:_:SER | 3.409 | 54.099 | 17 | O | N | A:130:_:SER |  |  |  | 1 |
| A:125:_:ALA | HBOND:MC_MC | A:131:_:ASP | 3.441 | 15.37 | 17 | O | N | A:131:_:ASP |  |  |  | 1 |
| A:126:_:THR | HBOND:MC_MC | A:130:_:SER | 3.114 | 50.634 | 17 | O | N | A:130:_:SER |  |  |  | 1 |
| A:127:_:ILE | VDW:SC_MC | A:210:_:TRP | 3.827 | NaN | 6 | CG2 | C |  |  |  |  | 1 |
| A:128:_:ILE | VDW:SC_MC | A:213:_:ASP | 3.8 | NaN | 6 | CG2 | C |  |  |  |  | 1 |
| A:128:_:ILE | VDW:MC_SC | A:214:_:ASN | 3.518 | NaN | 6 | C | OD1 |  |  |  |  | 1 |
| A:128:_:ILE | HBOND:MC_MC | A:215:_:GLU | 2.945 | 23.902 | 17 | O | N | A:215:_:GLU |  |  |  | 1 |
| A:129:_:THR | VDW:MC_SC | A:216:_:THR | 3.797 | NaN | 6 | C | CG2 |  |  |  |  | 1 |
| A:131:_:ASP | HBOND:SC_MC | A:134:_:ALA | 2.913 | 19.617 | 17 | OD1 | N | A:134:_:ALA |  |  |  | 1 |
| A:131:_:ASP | VDW:SC_SC | A:134:_:ALA | 3.762 | NaN | 6 | CG | CB |  |  |  |  | 1 |
| A:131:_:ASP | HBOND:MC_MC | A:135:_:ALA | 3.26 | 20.59 | 17 | O | N | A:135:_:ALA |  |  |  | 1 |
| A:132:_:ASN | HBOND:MC_MC | A:135:_:ALA | 3.308 | 48.389 | 17 | O | N | A:135:_:ALA |  |  |  | 1 |
| A:132:_:ASN | HBOND:MC_MC | A:136:_:ASN | 3.118 | 27.767 | 17 | O | N | A:136:_:ASN |  |  |  | 1 |
| A:132:_:ASN | VDW:SC_SC | A:166:_:GLU | 3.001 | NaN | 6 | OD1 | CD |  |  |  |  | 1 |
| A:133:_:THR | HBOND:MC_MC | A:136:_:ASN | 3.255 | 36.579 | 17 | O | N | A:136:_:ASN |  |  |  | 1 |
| A:133:_:THR | HBOND:MC_MC | A:137:_:LEU | 3.04 | 26.206 | 17 | O | N | A:137:_:LEU |  |  |  | 1 |
| A:134:_:ALA | HBOND:MC_MC | A:137:_:LEU | 3.338 | 39.193 | 17 | O | N | A:137:_:LEU |  |  |  | 1 |
| A:134:_:ALA | HBOND:MC_MC | A:138:_:LEU | 2.949 | 35.262 | 17 | O | N | A:138:_:LEU |  |  |  | 1 |
| A:135:_:ALA | HBOND:MC_MC | A:138:_:LEU | 3.166 | 34.357 | 17 | O | N | A:138:_:LEU |  |  |  | 1 |
| A:135:_:ALA | HBOND:MC_MC | A:139:_:PHE | 2.887 | 26.216 | 17 | O | N | A:139:_:PHE |  |  |  | 1 |
| A:136:_:ASN | HBOND:MC_MC | A:139:_:PHE | 3.229 | 40.923 | 17 | O | N | A:139:_:PHE |  |  |  | 1 |
| A:136:_:ASN | HBOND:MC_MC | A:140:_:GLY | 3.097 | 33.024 | 17 | O | N | A:140:_:GLY |  |  |  | 1 |
| A:136:_:ASN | VDW:SC_SC | A:165:_:LEU | 3.386 | NaN | 6 | OD1 | CG |  |  |  |  | 1 |
| A:136:_:ASN | VDW:SC_SC | A:165:_:LEU | 3.734 | NaN | 6 | CG | CD2 |  |  |  |  | 1 |
| A:136:_:ASN | HBOND:SC_MC | A:166:_:GLU | 2.875 | 11.348 | 17 | OD1 | N | A:166:_:GLU |  |  |  | 1 |
| A:136:_:ASN | VDW:SC_SC | A:166:_:GLU | 3.35 | NaN | 6 | OD1 | CB |  |  |  |  | 1 |
| A:137:_:LEU | HBOND:MC_MC | A:140:_:GLY | 3.167 | 36.332 | 17 | O | N | A:140:_:GLY |  |  |  | 1 |
| A:137:_:LEU | HBOND:MC_MC | A:141:_:VAL | 3.036 | 28.041 | 17 | O | N | A:141:_:VAL |  |  |  | 1 |
| A:138:_:LEU | HBOND:MC_MC | A:141:_:VAL | 3.087 | 41.797 | 17 | O | N | A:141:_:VAL |  |  |  | 1 |
| A:138:_:LEU | HBOND:MC_MC | A:142:_:VAL | 2.93 | 35.837 | 17 | O | N | A:142:_:VAL |  |  |  | 1 |
| A:139:_:PHE | HBOND:MC_MC | A:142:_:VAL | 3.327 | 27.228 | 17 | O | N | A:142:_:VAL |  |  |  | 1 |
| A:139:_:PHE | HBOND:MC_MC | A:143:_:GLY | 3.094 | 50.95 | 17 | O | N | A:143:_:GLY |  |  |  | 1 |
| A:139:_:PHE | HBOND:MC_MC | A:144:_:GLY | 2.769 | 13.057 | 17 | O | N | A:144:_:GLY |  |  |  | 1 |
| A:139:_:PHE | VDW:SC_MC | A:144:_:GLY | 3.449 | NaN | 6 | CD1 | C |  |  |  |  | 1 |
| A:139:_:PHE | VDW:SC_SC | A:148:_:VAL | 3.827 | NaN | 6 | CE1 | CG2 |  |  |  |  | 1 |
| A:139:_:PHE | VDW:SC_SC | A:165:_:LEU | 3.433 | NaN | 6 | CD2 | CG |  |  |  |  | 1 |
| A:139:_:PHE | VDW:SC_SC | A:165:_:LEU | 3.67 | NaN | 6 | CG | CD1 |  |  |  |  | 1 |
| A:142:_:VAL | VDW:MC_SC | A:147:_:ALA | 3.84 | NaN | 6 | C | CB |  |  |  |  | 1 |
| A:143:_:GLY | HBOND:MC_MC | A:147:_:ALA | 3.268 | 37.213 | 17 | O | N | A:147:_:ALA |  |  |  | 1 |
| A:143:_:GLY | VDW:MC_SC | A:147:_:ALA | 3.536 | NaN | 6 | C | CB |  |  |  |  | 1 |
| A:144:_:GLY | HBOND:MC_MC | A:147:_:ALA | 2.974 | 28.697 | 17 | O | N | A:147:_:ALA |  |  |  | 1 |
| A:144:_:GLY | HBOND:MC_MC | A:148:_:VAL | 3.185 | 28.017 | 17 | O | N | A:148:_:VAL |  |  |  | 1 |
| A:145:_:PRO | HBOND:MC_MC | A:148:_:VAL | 3.209 | 36.02 | 17 | O | N | A:148:_:VAL |  |  |  | 1 |
| A:145:_:PRO | HBOND:MC_MC | A:149:_:THR | 2.885 | 9.258 | 17 | O | N | A:149:_:THR |  |  |  | 1 |
| A:145:_:PRO | VDW:SC_MC | A:162:_:SER | 3.535 | NaN | 6 | CB | C |  |  |  |  | 1 |
| A:145:_:PRO | VDW:SC_MC | A:163:_:ASP | 3.495 | NaN | 6 | CB | C |  |  |  |  | 1 |
| A:145:_:PRO | VDW:SC_SC | A:165:_:LEU | 3.591 | NaN | 6 | CD | CD1 |  |  |  |  | 1 |
| A:146:_:PRO | HBOND:MC_MC | A:149:_:THR | 3.488 | 57.724 | 17 | O | N | A:149:_:THR |  |  |  | 1 |
| A:146:_:PRO | HBOND:MC_MC | A:150:_:ALA | 2.854 | 29.57 | 17 | O | N | A:150:_:ALA |  |  |  | 1 |
| A:147:_:ALA | HBOND:MC_MC | A:150:_:ALA | 3.361 | 42.644 | 17 | O | N | A:150:_:ALA |  |  |  | 1 |
| A:147:_:ALA | HBOND:MC_MC | A:151:_:PHE | 3.016 | 25.651 | 17 | O | N | A:151:_:PHE |  |  |  | 1 |
| A:148:_:VAL | HBOND:MC_MC | A:151:_:PHE | 3.266 | 43.365 | 17 | O | N | A:151:_:PHE |  |  |  | 1 |
| A:148:_:VAL | HBOND:MC_MC | A:152:_:LEU | 2.939 | 25.318 | 17 | O | N | A:152:_:LEU |  |  |  | 1 |
| A:148:_:VAL | VDW:SC_SC | A:162:_:SER | 3.845 | NaN | 6 | CG1 | CB |  |  |  |  | 1 |
| A:149:_:THR | HBOND:MC_MC | A:152:_:LEU | 3.156 | 44.28 | 17 | O | N | A:152:_:LEU |  |  |  | 1 |
| A:149:_:THR | HBOND:MC_MC | A:153:_:ARG | 2.827 | 22.185 | 17 | O | N | A:153:_:ARG |  |  |  | 1 |
| A:149:_:THR | HBOND:SC_MC | A:162:_:SER | 3.101 | 19.055 | 17 | OG1 | N | A:162:_:SER |  |  |  | 1 |
| A:150:_:ALA | HBOND:MC_MC | A:153:_:ARG | 3.359 | 43.852 | 17 | O | N | A:153:_:ARG |  |  |  | 1 |
| A:150:_:ALA | HBOND:MC_MC | A:154:_:ALA | 2.878 | 21.46 | 17 | O | N | A:154:_:ALA |  |  |  | 1 |
| A:151:_:PHE | HBOND:MC_MC | A:154:_:ALA | 3.331 | 44.817 | 17 | O | N | A:154:_:ALA |  |  |  | 1 |
| A:151:_:PHE | HBOND:MC_MC | A:155:_:SER | 2.983 | 38.665 | 17 | O | N | A:155:_:SER |  |  |  | 1 |
| A:151:_:PHE | VDW:SC_SC | A:198:_:VAL | 3.723 | NaN | 6 | CE1 | CG1 |  |  |  |  | 1 |
| A:152:_:LEU | HBOND:MC_MC | A:155:_:SER | 3.119 | 30.105 | 17 | O | N | A:155:_:SER |  |  |  | 1 |
| A:152:_:LEU | HBOND:MC_MC | A:156:_:GLY | 3.251 | 45.254 | 17 | O | N | A:156:_:GLY |  |  |  | 1 |
| A:152:_:LEU | HBOND:MC_MC | A:157:_:ASP | 2.972 | 14.96 | 17 | O | N | A:157:_:ASP |  |  |  | 1 |
| A:153:_:ARG | HBOND:MC_MC | A:156:_:GLY | 2.974 | 21.321 | 17 | O | N | A:156:_:GLY |  |  |  | 1 |
| A:155:_:SER | VDW:SC_SC | A:198:_:VAL | 3.899 | NaN | 6 | CB | CG2 |  |  |  |  | 1 |
| A:157:_:ASP | HBOND:SC_MC | A:160:_:SER | 2.815 | 4.766 | 17 | OD1 | N | A:160:_:SER |  |  |  | 1 |
| A:157:_:ASP | VDW:SC_SC | A:160:_:SER | 3.702 | NaN | 6 | CG | CB |  |  |  |  | 1 |
| A:159:_:VAL | VDW:SC_SC | A:182:_:THR | 3.535 | NaN | 6 | CG1 | CG2 |  |  |  |  | 1 |
| A:159:_:VAL | VDW:SC_SC | A:185:_:ALA | 3.579 | NaN | 6 | CB | CB |  |  |  |  | 1 |
| A:161:_:ARG | HBOND:MC_MC | A:179:_:ASP | 3.321 | 56.957 | 17 | O | N | A:179:_:ASP |  |  |  | 1 |
| A:161:_:ARG | HBOND:MC_MC | A:180:_:THR | 3.096 | 39.586 | 17 | N | O | A:161:_:ARG |  |  |  | 1 |
| A:163:_:ASP | VDW:SC_SC | A:178:_:ARG | 3.854 | NaN | 6 | CG | CG |  |  |  |  | 1 |
| A:163:_:ASP | VDW:SC_SC | A:178:_:ARG | 3.865 | NaN | 6 | CB | CB |  |  |  |  | 1 |
| A:163:_:ASP | HBOND:MC_SC | A:179:_:ASP | 2.767 | 14.779 | 17 | N | OD1 | A:163:_:ASP |  |  |  | 1 |
| A:163:_:ASP | HBOND:SC_MC | A:179:_:ASP | 2.93 | 15.325 | 17 | OD2 | N | A:179:_:ASP |  |  |  | 1 |
| A:164:_:ARG | VDW:MC_SC | A:169:_:LEU | 3.856 | NaN | 6 | C | CB |  |  |  |  | 1 |
| A:164:_:ARG | HBOND:MC_SC | A:179:_:ASP | 3.035 | 13.588 | 17 | N | OD1 | A:164:_:ARG |  |  |  | 1 |
| A:165:_:LEU | HBOND:MC_MC | A:168:_:GLU | 3.084 | 50.388 | 17 | O | N | A:168:_:GLU |  |  |  | 1 |
| A:165:_:LEU | HBOND:MC_MC | A:169:_:LEU | 2.95 | 28.336 | 17 | O | N | A:169:_:LEU |  |  |  | 1 |
| A:165:_:LEU | VDW:MC_SC | A:169:_:LEU | 3.551 | NaN | 6 | C | CD2 |  |  |  |  | 1 |
| A:166:_:GLU | VDW:SC_SC | A:169:_:LEU | 3.793 | NaN | 6 | CD | CD2 |  |  |  |  | 1 |
| A:167:_:PRO | HBOND:MC_MC | A:170:_:ASN | 3.149 | 12.484 | 17 | O | N | A:170:_:ASN |  |  |  | 1 |
| A:168:_:GLU | HBOND:MC_MC | A:171:_:SER | 3.03 | 9.969 | 17 | O | N | A:171:_:SER |  |  |  | 1 |
| A:169:_:LEU | VDW:SC_SC | A:179:_:ASP | 3.62 | NaN | 6 | CB | CG |  |  |  |  | 1 |
| A:170:_:ASN | VDW:SC_SC | A:238:_:ASN | 3.797 | NaN | 6 | CB | CB |  |  |  |  | 1 |
| A:172:_:PHE | VDW:SC_SC | A:180:_:THR | 3.85 | NaN | 6 | CE1 | CG2 |  |  |  |  | 1 |
| A:172:_:PHE | VDW:SC_SC | A:266:_:GLN | 3.483 | NaN | 6 | CE2 | OE1 |  |  |  |  | 1 |
| A:173:_:ALA | HBOND:MC_MC | A:176:_:ASP | 2.96 | 19.981 | 17 | O | N | A:176:_:ASP |  |  |  | 1 |
| A:177:_:PRO | HBOND:MC_MC | A:180:_:THR | 3.281 | 47.575 | 17 | O | N | A:180:_:THR |  |  |  | 1 |
| A:177:_:PRO | VDW:MC_SC | A:180:_:THR | 3.631 | NaN | 6 | C | CG2 |  |  |  |  | 1 |
| A:181:_:THR | VDW:SC_SC | A:264:_:TYR | 3.539 | NaN | 6 | CG2 | CZ |  |  |  |  | 1 |
| A:182:_:THR | HBOND:SC_MC | A:185:_:ALA | 3.012 | 24.231 | 17 | OG1 | N | A:185:_:ALA |  |  |  | 1 |
| A:182:_:THR | HBOND:MC_MC | A:185:_:ALA | 3.236 | 36.282 | 17 | O | N | A:185:_:ALA |  |  |  | 1 |
| A:182:_:THR | HBOND:MC_MC | A:186:_:MET | 3.021 | 19.946 | 17 | O | N | A:186:_:MET |  |  |  | 1 |
| A:183:_:PRO | HBOND:MC_MC | A:187:_:ALA | 2.982 | 36.985 | 17 | O | N | A:187:_:ALA |  |  |  | 1 |
| A:184:_:ALA | HBOND:MC_MC | A:187:_:ALA | 3.361 | 34.709 | 17 | O | N | A:187:_:ALA |  |  |  | 1 |
| A:184:_:ALA | HBOND:MC_MC | A:188:_:ALA | 3.038 | 21.962 | 17 | O | N | A:188:_:ALA |  |  |  | 1 |
| A:185:_:ALA | HBOND:MC_MC | A:188:_:ALA | 3.282 | 44.12 | 17 | O | N | A:188:_:ALA |  |  |  | 1 |
| A:185:_:ALA | HBOND:MC_MC | A:189:_:THR | 2.873 | 28.9 | 17 | O | N | A:189:_:THR |  |  |  | 1 |
| A:186:_:MET | HBOND:MC_MC | A:189:_:THR | 3.287 | 42.111 | 17 | O | N | A:189:_:THR |  |  |  | 1 |
| A:186:_:MET | HBOND:MC_MC | A:190:_:LEU | 2.845 | 28.228 | 17 | O | N | A:190:_:LEU |  |  |  | 1 |
| A:186:_:MET | VDW:SC_SC | A:247:_:ILE | 3.67 | NaN | 6 | CG | CD1 |  |  |  |  | 1 |
| A:187:_:ALA | HBOND:MC_MC | A:190:_:LEU | 3.324 | 37.305 | 17 | O | N | A:190:_:LEU |  |  |  | 1 |
| A:187:_:ALA | HBOND:MC_MC | A:191:_:GLN | 3.036 | 30.448 | 17 | O | N | A:191:_:GLN |  |  |  | 1 |
| A:187:_:ALA | VDW:SC_SC | A:262:_:THR | 3.863 | NaN | 6 | CB | CG2 |  |  |  |  | 1 |
| A:188:_:ALA | HBOND:MC_MC | A:191:_:GLN | 3.224 | 36.73 | 17 | O | N | A:191:_:GLN |  |  |  | 1 |
| A:188:_:ALA | HBOND:MC_MC | A:192:_:ARG | 2.921 | 25.583 | 17 | O | N | A:192:_:ARG |  |  |  | 1 |
| A:189:_:THR | HBOND:MC_MC | A:192:_:ARG | 3.241 | 43.485 | 17 | O | N | A:192:_:ARG |  |  |  | 1 |
| A:189:_:THR | HBOND:MC_MC | A:193:_:VAL | 2.834 | 29.549 | 17 | O | N | A:193:_:VAL |  |  |  | 1 |
| A:190:_:LEU | HBOND:MC_MC | A:193:_:VAL | 3.496 | 33.537 | 17 | O | N | A:193:_:VAL |  |  |  | 1 |
| A:190:_:LEU | HBOND:MC_MC | A:194:_:VAL | 2.804 | 14.523 | 17 | O | N | A:194:_:VAL |  |  |  | 1 |
| A:190:_:LEU | VDW:SC_SC | A:194:_:VAL | 3.867 | NaN | 6 | CD2 | CG2 |  |  |  |  | 1 |
| A:190:_:LEU | VDW:SC_SC | A:247:_:ILE | 3.814 | NaN | 6 | CD1 | CB |  |  |  |  | 1 |
| A:190:_:LEU | VDW:SC_SC | A:260:_:VAL | 3.845 | NaN | 6 | CB | CG1 |  |  |  |  | 1 |
| A:191:_:GLN | HBOND:MC_MC | A:195:_:LEU | 3.033 | 27.635 | 17 | O | N | A:195:_:LEU |  |  |  | 1 |
| A:191:_:GLN | HBOND:MC_MC | A:196:_:GLY | 2.847 | 43.265 | 17 | O | N | A:196:_:GLY |  |  |  | 1 |
| A:192:_:ARG | HBOND:MC_MC | A:196:_:GLY | 3.212 | 45.477 | 17 | O | N | A:196:_:GLY |  |  |  | 1 |
| A:192:_:ARG | HBOND:MC_MC | A:198:_:VAL | 3.451 | 18.867 | 17 | O | N | A:198:_:VAL |  |  |  | 1 |
| A:192:_:ARG | VDW:SC_SC | A:198:_:VAL | 3.731 | NaN | 6 | CB | CG2 |  |  |  |  | 1 |
| A:193:_:VAL | VDW:SC_SC | A:207:_:LEU | 3.794 | NaN | 6 | CG1 | CD2 |  |  |  |  | 1 |
| A:194:_:VAL | VDW:MC_SC | A:204:_:ARG | 3.691 | NaN | 6 | C | CD |  |  |  |  | 1 |
| A:194:_:VAL | VDW:SC_SC | A:211:_:LEU | 3.826 | NaN | 6 | CG2 | CD1 |  |  |  |  | 1 |
| A:195:_:LEU | VDW:MC_SC | A:204:_:ARG | 3.612 | NaN | 6 | C | CD |  |  |  |  | 1 |
| A:195:_:LEU | VDW:SC_SC | A:251:_:TRP | 3.775 | NaN | 6 | CD2 | CZ2 |  |  |  |  | 1 |
| A:195:_:LEU | VDW:SC_SC | A:258:_:PRO | 3.841 | NaN | 6 | CD1 | CB |  |  |  |  | 1 |
| A:199:_:LEU | VDW:SC_SC | A:203:_:SER | 3.744 | NaN | 6 | CB | CB |  |  |  |  | 1 |
| A:200:_:GLN | HBOND:MC_MC | A:203:_:SER | 2.952 | 34.121 | 17 | O | N | A:203:_:SER |  |  |  | 1 |
| A:200:_:GLN | HBOND:MC_MC | A:204:_:ARG | 2.979 | 12.802 | 17 | O | N | A:204:_:ARG |  |  |  | 1 |
| A:201:_:PRO | HBOND:MC_MC | A:204:_:ARG | 3.302 | 50.199 | 17 | O | N | A:204:_:ARG |  |  |  | 1 |
| A:201:_:PRO | HBOND:MC_MC | A:205:_:GLN | 2.856 | 28.506 | 17 | O | N | A:205:_:GLN |  |  |  | 1 |
| A:201:_:PRO | VDW:MC_SC | A:205:_:GLN | 3.695 | NaN | 6 | C | OE1 |  |  |  |  | 1 |
| A:202:_:ALA | HBOND:MC_MC | A:206:_:GLN | 3.072 | 32.62 | 17 | O | N | A:206:_:GLN |  |  |  | 1 |
| A:203:_:SER | HBOND:MC_MC | A:206:_:GLN | 3.169 | 35.797 | 17 | O | N | A:206:_:GLN |  |  |  | 1 |
| A:203:_:SER | HBOND:MC_MC | A:207:_:LEU | 3.03 | 22.263 | 17 | O | N | A:207:_:LEU |  |  |  | 1 |
| A:204:_:ARG | HBOND:MC_MC | A:207:_:LEU | 3.262 | 44.059 | 17 | O | N | A:207:_:LEU |  |  |  | 1 |
| A:204:_:ARG | HBOND:MC_MC | A:208:_:ALA | 2.855 | 22.292 | 17 | O | N | A:208:_:ALA |  |  |  | 1 |
| A:205:_:GLN | HBOND:MC_MC | A:208:_:ALA | 3.37 | 46.509 | 17 | O | N | A:208:_:ALA |  |  |  | 1 |
| A:205:_:GLN | HBOND:MC_MC | A:209:_:ASP | 2.894 | 32.409 | 17 | O | N | A:209:_:ASP |  |  |  | 1 |
| A:205:_:GLN | VDW:SC_SC | B:94:_:ARG | 3.158 | NaN | 6 | OE1 | CD |  |  |  |  | 1 |
| A:205:_:GLN | VDW:SC_SC | B:94:_:ARG | 3.51 | NaN | 6 | CB | CZ |  |  |  |  | 1 |
| A:206:_:GLN | HBOND:MC_MC | A:209:_:ASP | 3.263 | 37.384 | 17 | O | N | A:209:_:ASP |  |  |  | 1 |
| A:206:_:GLN | HBOND:MC_MC | A:210:_:TRP | 3.074 | 28.732 | 17 | O | N | A:210:_:TRP |  |  |  | 1 |
| A:206:_:GLN | VDW:SC_SC | A:210:_:TRP | 3.345 | NaN | 6 | CG | CZ2 |  |  |  |  | 1 |
| A:206:_:GLN | VDW:SC_SC | A:210:_:TRP | 3.354 | NaN | 6 | OE1 | CE2 |  |  |  |  | 1 |
| A:207:_:LEU | HBOND:MC_MC | A:210:_:TRP | 3.173 | 38.265 | 17 | O | N | A:210:_:TRP |  |  |  | 1 |
| A:207:_:LEU | HBOND:MC_MC | A:211:_:LEU | 3.034 | 23.552 | 17 | O | N | A:211:_:LEU |  |  |  | 1 |
| A:208:_:ALA | HBOND:MC_MC | A:211:_:LEU | 3.144 | 41.627 | 17 | O | N | A:211:_:LEU |  |  |  | 1 |
| A:208:_:ALA | HBOND:MC_MC | A:212:_:ILE | 2.817 | 26.829 | 17 | O | N | A:212:_:ILE |  |  |  | 1 |
| A:208:_:ALA | VDW:SC_SC | A:251:_:TRP | 3.875 | NaN | 6 | CB | CZ2 |  |  |  |  | 1 |
| A:209:_:ASP | HBOND:MC_MC | A:212:_:ILE | 3.189 | 42.272 | 17 | O | N | A:212:_:ILE |  |  |  | 1 |
| A:209:_:ASP | HBOND:MC_MC | A:213:_:ASP | 2.959 | 34.698 | 17 | O | N | A:213:_:ASP |  |  |  | 1 |
| A:209:_:ASP | IONIC:SC_SC | B:94:_:ARG | 3.962 | 151.093 | 20 | 89.231,112.837,22.504 | CZ |  | B:94:_:ARG |  |  | 1 |
| A:209:_:ASP | IONIC:SC_SC | B:115:_:LYS | 3.276 | 137.302 | 20 | 89.231,112.837,22.504 | NZ |  | B:115:_:LYS |  |  | 1 |
| A:210:_:TRP | HBOND:MC_MC | A:213:_:ASP | 3.125 | 34.056 | 17 | O | N | A:213:_:ASP |  |  |  | 1 |
| A:211:_:LEU | HBOND:MC_MC | A:214:_:ASN | 3.085 | 30.908 | 17 | O | N | A:214:_:ASN |  |  |  | 1 |
| A:211:_:LEU | VDW:SC_MC | A:232:_:GLY | 3.816 | NaN | 6 | CB | C |  |  |  |  | 1 |
| A:211:_:LEU | HBOND:MC_MC | A:233:_:ASP | 3.036 | 22.148 | 17 | O | N | A:233:_:ASP |  |  |  | 1 |
| A:211:_:LEU | VDW:SC_MC | A:233:_:ASP | 3.799 | NaN | 6 | CD2 | C |  |  |  |  | 1 |
| A:212:_:ILE | VDW:SC_SC | A:230:_:ARG | 3.76 | NaN | 6 | CD1 | CD |  |  |  |  | 1 |
| A:212:_:ILE | VDW:SC_SC | A:251:_:TRP | 3.739 | NaN | 6 | CG1 | CZ3 |  |  |  |  | 1 |
| A:212:_:ILE | VDW:SC_SC | B:112:_:HIS | 3.469 | NaN | 6 | CG2 | CE1 |  |  |  |  | 1 |
| A:212:_:ILE | VDW:SC_SC | B:115:_:LYS | 3.809 | NaN | 6 | CD1 | CE |  |  |  |  | 1 |
| A:214:_:ASN | VDW:SC_SC | A:233:_:ASP | 3.328 | NaN | 6 | ND2 | CG |  |  |  |  | 1 |
| A:217:_:GLY | HBOND:MC_MC | A:220:_:CYS | 3.015 | 13.167 | 17 | O | N | A:220:_:CYS |  |  |  | 1 |
| A:219:_:ALA | VDW:MC_SC | A:223:_:ALA | 3.855 | NaN | 6 | C | CB |  |  |  |  | 1 |
| A:220:_:CYS | HBOND:MC_MC | A:223:_:ALA | 2.985 | 19.988 | 17 | O | N | A:223:_:ALA |  |  |  | 1 |
| A:220:_:CYS | VDW:SC_SC | A:276:_:ALA | 3.997 | NaN | 6 | SG | CB |  |  |  |  | 1 |
| A:220:_:CYS | VDW:SC_SC | A:279:_:LEU | 3.893 | NaN | 6 | SG | CD1 |  |  |  |  | 1 |
| A:221:_:LEU | HBOND:MC_MC | A:224:_:GLY | 2.892 | 13.674 | 17 | O | N | A:224:_:GLY |  |  |  | 1 |
| A:221:_:LEU | HBOND:MC_MC | A:225:_:LEU | 3.368 | 54.222 | 17 | O | N | A:225:_:LEU |  |  |  | 1 |
| A:221:_:LEU | HBOND:MC_SC | A:246:_:ASP | 3.017 | 10.218 | 17 | N | OD2 | A:221:_:LEU |  |  |  | 1 |
| A:221:_:LEU | VDW:SC_SC | A:246:_:ASP | 3.714 | NaN | 6 | CD1 | CG |  |  |  |  | 1 |
| A:221:_:LEU | VDW:SC_SC | A:248:_:ALA | 3.775 | NaN | 6 | CD1 | CB |  |  |  |  | 1 |
| A:221:_:LEU | VDW:SC_SC | A:261:_:LEU | 3.819 | NaN | 6 | CD2 | CD2 |  |  |  |  | 1 |
| A:221:_:LEU | VDW:SC_SC | A:282:_:VAL | 3.797 | NaN | 6 | CD2 | CG1 |  |  |  |  | 1 |
| A:222:_:ARG | HBOND:MC_MC | A:225:_:LEU | 3.218 | 10.682 | 17 | O | N | A:225:_:LEU |  |  |  | 1 |
| A:223:_:ALA | VDW:SC_SC | A:280:_:ALA | 3.825 | NaN | 6 | CB | CB |  |  |  |  | 1 |
| A:224:_:GLY | VDW:MC_MC | A:283:_:GLY | 3.861 | NaN | 6 | C | C |  |  |  |  | 1 |
| A:226:_:GLY | HBOND:MC_MC | A:229:_:TRP | 3.156 | 22.733 | 17 | O | N | A:229:_:TRP |  |  |  | 1 |
| A:226:_:GLY | HBOND:MC_SC | A:287:_:ASP | 2.892 | 24.811 | 17 | N | OD2 | A:226:_:GLY |  |  |  | 1 |
| A:228:_:ARG | HBOND:MC_MC | A:253:_:VAL | 2.908 | 16.76 | 17 | O | N | A:253:_:VAL |  |  |  | 1 |
| A:228:_:ARG | HBOND:SC_MC | A:291:_:GLY | 2.898 | 33.417 | 17 | NH2 | N | A:291:_:GLY |  |  |  | 1 |
| A:229:_:TRP | VDW:SC_SC | A:250:_:LEU | 3.72 | NaN | 6 | CE3 | CB |  |  |  |  | 1 |
| A:229:_:TRP | VDW:SC_MC | A:251:_:TRP | 3.683 | NaN | 6 | CE3 | C |  |  |  |  | 1 |
| A:229:_:TRP | VDW:SC_SC | A:252:_:PRO | 3.85 | NaN | 6 | CZ3 | CB |  |  |  |  | 1 |
| A:229:_:TRP | VDW:SC_SC | A:290:_:ILE | 3.681 | NaN | 6 | CZ2 | CG2 |  |  |  |  | 1 |
| A:230:_:ARG | HBOND:MC_MC | A:251:_:TRP | 2.826 | 6.617 | 17 | N | O | A:230:_:ARG |  |  |  | 1 |
| A:230:_:ARG | HBOND:MC_MC | A:251:_:TRP | 2.838 | 1.921 | 17 | O | N | A:251:_:TRP |  |  |  | 1 |
| A:230:_:ARG | VDW:SC_SC | B:115:_:LYS | 3.565 | NaN | 6 | CD | CD |  |  |  |  | 1 |
| A:232:_:GLY | HBOND:MC_MC | A:249:_:VAL | 2.87 | 14.642 | 17 | O | N | A:249:_:VAL |  |  |  | 1 |
| A:232:_:GLY | HBOND:MC_MC | A:249:_:VAL | 2.942 | 5.795 | 17 | N | O | A:232:_:GLY |  |  |  | 1 |
| A:233:_:ASP | VDW:SC_SC | A:248:_:ALA | 3.846 | NaN | 6 | CB | CB |  |  |  |  | 1 |
| A:234:_:LYS | HBOND:MC_MC | A:247:_:ILE | 2.889 | 15.319 | 17 | N | O | A:234:_:LYS |  |  |  | 1 |
| A:234:_:LYS | HBOND:MC_MC | A:247:_:ILE | 3.006 | 10.336 | 17 | O | N | A:247:_:ILE |  |  |  | 1 |
| A:236:_:GLY | HBOND:MC_MC | A:245:_:ASN | 2.766 | 9.939 | 17 | O | N | A:245:_:ASN |  |  |  | 1 |
| A:236:_:GLY | HBOND:MC_MC | A:245:_:ASN | 2.936 | 27.852 | 17 | N | O | A:236:_:GLY |  |  |  | 1 |
| A:238:_:ASN | HBOND:SC_MC | A:242:_:ASP | 3.091 | 27.994 | 17 | OD1 | N | A:242:_:ASP |  |  |  | 1 |
| A:238:_:ASN | HBOND:SC_MC | A:243:_:ALA | 2.979 | 21.229 | 17 | OD1 | N | A:243:_:ALA |  |  |  | 1 |
| A:238:_:ASN | HBOND:MC_MC | A:243:_:ALA | 3.217 | 18.019 | 17 | N | O | A:238:_:ASN |  |  |  | 1 |
| A:238:_:ASN | VDW:SC_SC | A:243:_:ALA | 3.493 | NaN | 6 | OD1 | CB |  |  |  |  | 1 |
| A:242:_:ASP | VDW:SC_SC | A:266:_:GLN | 3.55 | NaN | 6 | CG | NE2 |  |  |  |  | 1 |
| A:242:_:ASP | HBOND:MC_MC | A:267:_:ALA | 2.925 | 28.073 | 17 | O | N | A:267:_:ALA |  |  |  | 1 |
| A:242:_:ASP | VDW:SC_MC | A:267:_:ALA | 3.861 | NaN | 6 | CB | C |  |  |  |  | 1 |
| A:244:_:ARG | HBOND:MC_MC | A:265:_:LEU | 2.925 | 14.803 | 17 | N | O | A:244:_:ARG |  |  |  | 1 |
| A:244:_:ARG | HBOND:MC_MC | A:265:_:LEU | 3.091 | 49.746 | 17 | O | N | A:265:_:LEU |  |  |  | 1 |
| A:244:_:ARG | VDW:SC_SC | A:272:_:TYR | 3.356 | NaN | 6 | CZ | CZ |  |  |  |  | 1 |
| A:244:_:ARG | VDW:SC_SC | A:272:_:TYR | 3.682 | NaN | 6 | CD | CE1 |  |  |  |  | 1 |
| A:244:_:ARG | VDW:SC_SC | A:275:_:ARG | 3.723 | NaN | 6 | CB | CB |  |  |  |  | 1 |
| A:244:_:ARG | VDW:SC_SC | A:276:_:ALA | 3.791 | NaN | 6 | CZ | CB |  |  |  |  | 1 |
| A:244:_:ARG | VDW:SC_SC | A:279:_:LEU | 3.803 | NaN | 6 | CG | CD1 |  |  |  |  | 1 |
| A:245:_:ASN | VDW:SC_SC | A:264:_:TYR | 3.079 | NaN | 6 | CG | CD2 |  |  |  |  | 1 |
| A:245:_:ASN | HBOND:SC_MC | A:265:_:LEU | 3.273 | 16.875 | 17 | OD1 | N | A:265:_:LEU |  |  |  | 1 |
| A:245:_:ASN | VDW:MC_SC | A:279:_:LEU | 3.809 | NaN | 6 | C | CD1 |  |  |  |  | 1 |
| A:246:_:ASP | HBOND:MC_MC | A:263:_:ALA | 2.829 | 15.855 | 17 | N | O | A:246:_:ASP |  |  |  | 1 |
| A:246:_:ASP | HBOND:MC_MC | A:263:_:ALA | 2.909 | 26.902 | 17 | O | N | A:263:_:ALA |  |  |  | 1 |
| A:247:_:ILE | VDW:SC_SC | A:262:_:THR | 3.851 | NaN | 6 | CG2 | CG2 |  |  |  |  | 1 |
| A:247:_:ILE | VDW:SC_SC | A:264:_:TYR | 3.633 | NaN | 6 | CG2 | CE1 |  |  |  |  | 1 |
| A:248:_:ALA | HBOND:MC_MC | A:261:_:LEU | 2.819 | 35.73 | 17 | N | O | A:248:_:ALA |  |  |  | 1 |
| A:248:_:ALA | HBOND:MC_MC | A:261:_:LEU | 2.875 | 10.999 | 17 | O | N | A:261:_:LEU |  |  |  | 1 |
| A:250:_:LEU | HBOND:MC_MC | A:259:_:TRP | 2.799 | 6.005 | 17 | N | O | A:250:_:LEU |  |  |  | 1 |
| A:250:_:LEU | HBOND:MC_MC | A:259:_:TRP | 2.912 | 13.333 | 17 | O | N | A:259:_:TRP |  |  |  | 1 |
| A:251:_:TRP | VDW:SC_SC | A:258:_:PRO | 3.708 | NaN | 6 | CD1 | CG |  |  |  |  | 1 |
| A:252:_:PRO | HBOND:MC_MC | A:255:_:GLY | 2.995 | 20.935 | 17 | O | N | A:255:_:GLY |  |  |  | 1 |
| A:252:_:PRO | VDW:SC_MC | A:255:_:GLY | 3.503 | NaN | 6 | CG | C |  |  |  |  | 1 |
| A:252:_:PRO | VDW:SC_MC | A:256:_:GLY | 3.535 | NaN | 6 | CG | C |  |  |  |  | 1 |
| A:252:_:PRO | VDW:SC_MC | A:257:_:ALA | 3.883 | NaN | 6 | CD | C |  |  |  |  | 1 |
| A:259:_:TRP | VDW:SC_SC | A:290:_:ILE | 3.582 | NaN | 6 | CE2 | CG2 |  |  |  |  | 1 |
| A:259:_:TRP | VDW:SC_SC | A:290:_:ILE | 3.779 | NaN | 6 | CD2 | CG1 |  |  |  |  | 1 |
| A:261:_:LEU | VDW:SC_SC | A:286:_:ALA | 3.657 | NaN | 6 | CD2 | CB |  |  |  |  | 1 |
| A:263:_:ALA | VDW:SC_SC | A:279:_:LEU | 3.742 | NaN | 6 | CB | CD2 |  |  |  |  | 1 |
| A:265:_:LEU | VDW:SC_SC | A:278:_:VAL | 3.722 | NaN | 6 | CD2 | CG1 |  |  |  |  | 1 |
| A:267:_:ALA | VDW:SC_SC | A:270:_:ILE | 3.763 | NaN | 6 | CB | CG1 |  |  |  |  | 1 |
| A:270:_:ILE | VDW:SC_SC | A:274:_:GLN | 3.291 | NaN | 6 | CB | OE1 |  |  |  |  | 1 |
| A:270:_:ILE | VDW:SC_MC | A:274:_:GLN | 3.698 | NaN | 6 | CD1 | C |  |  |  |  | 1 |
| A:270:_:ILE | VDW:SC_SC | A:275:_:ARG | 3.836 | NaN | 6 | CD1 | CG |  |  |  |  | 1 |
| A:270:_:ILE | VDW:SC_SC | A:278:_:VAL | 3.827 | NaN | 6 | CD1 | CG2 |  |  |  |  | 1 |
| A:271:_:SER | HBOND:MC_SC | A:274:_:GLN | 2.919 | 14.607 | 17 | N | OE1 | A:271:_:SER |  |  |  | 1 |
| A:271:_:SER | HBOND:SC_MC | A:274:_:GLN | 3.034 | 27.668 | 17 | OG | N | A:274:_:GLN |  |  |  | 1 |
| A:271:_:SER | HBOND:MC_MC | A:274:_:GLN | 3.057 | 37.679 | 17 | O | N | A:274:_:GLN |  |  |  | 1 |
| A:271:_:SER | HBOND:MC_MC | A:275:_:ARG | 2.891 | 40.069 | 17 | O | N | A:275:_:ARG |  |  |  | 1 |
| A:272:_:TYR | HBOND:MC_MC | A:275:_:ARG | 3.07 | 29.002 | 17 | O | N | A:275:_:ARG |  |  |  | 1 |
| A:272:_:TYR | HBOND:MC_MC | A:276:_:ALA | 3.257 | 30.314 | 17 | O | N | A:276:_:ALA |  |  |  | 1 |
| A:273:_:GLU | HBOND:MC_MC | A:276:_:ALA | 3.154 | 34.369 | 17 | O | N | A:276:_:ALA |  |  |  | 1 |
| A:273:_:GLU | HBOND:MC_MC | A:277:_:SER | 3.102 | 30.983 | 17 | O | N | A:277:_:SER |  |  |  | 1 |
| A:274:_:GLN | HBOND:MC_MC | A:277:_:SER | 3.052 | 41.359 | 17 | O | N | A:277:_:SER |  |  |  | 1 |
| A:274:_:GLN | HBOND:MC_MC | A:278:_:VAL | 2.917 | 34.008 | 17 | O | N | A:278:_:VAL |  |  |  | 1 |
| A:275:_:ARG | HBOND:MC_MC | A:278:_:VAL | 3.166 | 34.997 | 17 | O | N | A:278:_:VAL |  |  |  | 1 |
| A:275:_:ARG | HBOND:MC_MC | A:279:_:LEU | 3.09 | 30.015 | 17 | O | N | A:279:_:LEU |  |  |  | 1 |
| A:276:_:ALA | HBOND:MC_MC | A:279:_:LEU | 3.009 | 37.132 | 17 | O | N | A:279:_:LEU |  |  |  | 1 |
| A:276:_:ALA | HBOND:MC_MC | A:280:_:ALA | 2.841 | 24.432 | 17 | O | N | A:280:_:ALA |  |  |  | 1 |
| A:277:_:SER | HBOND:MC_MC | A:280:_:ALA | 3.365 | 42.984 | 17 | O | N | A:280:_:ALA |  |  |  | 1 |
| A:277:_:SER | HBOND:MC_MC | A:281:_:GLN | 2.983 | 33.461 | 17 | O | N | A:281:_:GLN |  |  |  | 1 |
| A:278:_:VAL | HBOND:MC_MC | A:281:_:GLN | 3.087 | 35.797 | 17 | O | N | A:281:_:GLN |  |  |  | 1 |
| A:278:_:VAL | HBOND:MC_MC | A:282:_:VAL | 2.935 | 32.283 | 17 | O | N | A:282:_:VAL |  |  |  | 1 |
| A:279:_:LEU | HBOND:MC_MC | A:282:_:VAL | 3.138 | 37.67 | 17 | O | N | A:282:_:VAL |  |  |  | 1 |
| A:279:_:LEU | HBOND:MC_MC | A:283:_:GLY | 3.114 | 17.687 | 17 | O | N | A:283:_:GLY |  |  |  | 1 |
| A:280:_:ALA | HBOND:MC_MC | A:283:_:GLY | 3.032 | 49.216 | 17 | O | N | A:283:_:GLY |  |  |  | 1 |
| A:280:_:ALA | HBOND:MC_MC | A:284:_:ARG | 2.929 | 34.67 | 17 | O | N | A:284:_:ARG |  |  |  | 1 |
| A:281:_:GLN | HBOND:MC_MC | A:284:_:ARG | 3.147 | 37.706 | 17 | O | N | A:284:_:ARG |  |  |  | 1 |
| A:281:_:GLN | VDW:SC_SC | A:284:_:ARG | 3.563 | NaN | 6 | OE1 | CD |  |  |  |  | 1 |
| A:281:_:GLN | HBOND:MC_MC | A:285:_:ILE | 2.93 | 10.558 | 17 | O | N | A:285:_:ILE |  |  |  | 1 |
| A:282:_:VAL | HBOND:MC_MC | A:285:_:ILE | 3.446 | 52.258 | 17 | O | N | A:285:_:ILE |  |  |  | 1 |
| A:282:_:VAL | HBOND:MC_MC | A:286:_:ALA | 2.869 | 24.334 | 17 | O | N | A:286:_:ALA |  |  |  | 1 |
| A:283:_:GLY | HBOND:MC_MC | A:286:_:ALA | 3.292 | 45.133 | 17 | O | N | A:286:_:ALA |  |  |  | 1 |
| A:283:_:GLY | HBOND:MC_MC | A:287:_:ASP | 2.9 | 31.112 | 17 | O | N | A:287:_:ASP |  |  |  | 1 |
| A:284:_:ARG | HBOND:MC_MC | A:287:_:ASP | 3.384 | 35.739 | 17 | O | N | A:287:_:ASP |  |  |  | 1 |
| A:284:_:ARG | HBOND:MC_MC | A:288:_:ARG | 3.259 | 31.024 | 17 | O | N | A:288:_:ARG |  |  |  | 1 |
| A:285:_:ILE | HBOND:MC_MC | A:288:_:ARG | 3.124 | 34.825 | 17 | O | N | A:288:_:ARG |  |  |  | 1 |
| A:285:_:ILE | HBOND:MC_MC | A:289:_:LEU | 3.036 | 28.465 | 17 | O | N | A:289:_:LEU |  |  |  | 1 |
| A:286:_:ALA | HBOND:MC_MC | A:289:_:LEU | 3.012 | 40.147 | 17 | O | N | A:289:_:LEU |  |  |  | 1 |
| A:286:_:ALA | HBOND:MC_MC | A:290:_:ILE | 3.125 | 49.703 | 17 | O | N | A:290:_:ILE |  |  |  | 1 |
| A:287:_:ASP | HBOND:MC_MC | A:290:_:ILE | 3.357 | 16.581 | 17 | O | N | A:290:_:ILE |  |  |  | 1 |
| B:19:_:ALA | HBOND:MC_MC | B:38:_:LYS | 3.111 | 29.61 | 17 | N | O | B:19:_:ALA |  |  |  | 1 |
| B:22:_:PRO | VDW:SC_MC | B:40:_:CYS | 3.82 | NaN | 6 | CG | C |  |  |  |  | 1 |
| B:22:_:PRO | VDW:SC_SC | B:270:_:ILE | 3.475 | NaN | 6 | CB | CG2 |  |  |  |  | 1 |
| B:23:_:THR | HBOND:SC_MC | B:26:_:ALA | 3.124 | 23.992 | 17 | OG1 | N | B:26:_:ALA |  |  |  | 1 |
| B:23:_:THR | HBOND:MC_MC | B:26:_:ALA | 3.237 | 37.301 | 17 | O | N | B:26:_:ALA |  |  |  | 1 |
| B:23:_:THR | HBOND:MC_MC | B:27:_:ILE | 3.141 | 27.009 | 17 | O | N | B:27:_:ILE |  |  |  | 1 |
| B:24:_:ASP | HBOND:MC_MC | B:27:_:ILE | 3.208 | 40.303 | 17 | O | N | B:27:_:ILE |  |  |  | 1 |
| B:24:_:ASP | HBOND:MC_MC | B:28:_:THR | 2.886 | 20.031 | 17 | O | N | B:28:_:THR |  |  |  | 1 |
| B:25:_:ALA | HBOND:MC_MC | B:28:_:THR | 3.362 | 46.814 | 17 | O | N | B:28:_:THR |  |  |  | 1 |
| B:25:_:ALA | HBOND:MC_MC | B:29:_:ALA | 2.987 | 34.496 | 17 | O | N | B:29:_:ALA |  |  |  | 1 |
| B:26:_:ALA | HBOND:MC_MC | B:29:_:ALA | 3.351 | 34.522 | 17 | O | N | B:29:_:ALA |  |  |  | 1 |
| B:26:_:ALA | HBOND:MC_MC | B:30:_:ALA | 3.304 | 47.876 | 17 | O | N | B:30:_:ALA |  |  |  | 1 |
| B:26:_:ALA | VDW:MC_SC | B:39:_:ALA | 3.826 | NaN | 6 | C | CB |  |  |  |  | 1 |
| B:27:_:ILE | HBOND:MC_MC | B:30:_:ALA | 3.26 | 14.807 | 17 | O | N | B:30:_:ALA |  |  |  | 1 |
| B:30:_:ALA | VDW:SC_MC | B:35:_:ALA | 3.845 | NaN | 6 | CB | C |  |  |  |  | 1 |
| B:30:_:ALA | VDW:SC_SC | B:36:_:LEU | 3.762 | NaN | 6 | CB | CB |  |  |  |  | 1 |
| B:31:_:SER | VDW:MC_SC | B:285:_:ILE | 3.836 | NaN | 6 | C | CD1 |  |  |  |  | 1 |
| B:32:_:ASP | HBOND:SC_MC | B:35:_:ALA | 2.898 | 28.015 | 17 | OD1 | N | B:35:_:ALA |  |  |  | 1 |
| B:32:_:ASP | HBOND:MC_MC | B:35:_:ALA | 3.253 | 37.952 | 17 | O | N | B:35:_:ALA |  |  |  | 1 |
| B:32:_:ASP | HBOND:MC_MC | B:36:_:LEU | 2.963 | 28.489 | 17 | O | N | B:36:_:LEU |  |  |  | 1 |
| B:32:_:ASP | VDW:MC_SC | B:285:_:ILE | 3.698 | NaN | 6 | C | CD1 |  |  |  |  | 1 |
| B:33:_:PHE | HBOND:MC_MC | B:36:_:LEU | 3.221 | 39.887 | 17 | O | N | B:36:_:LEU |  |  |  | 1 |
| B:33:_:PHE | HBOND:MC_MC | B:37:_:GLU | 2.981 | 14.15 | 17 | O | N | B:37:_:GLU |  |  |  | 1 |
| B:33:_:PHE | VDW:SC_SC | B:46:_:VAL | 3.686 | NaN | 6 | CZ | CG1 |  |  |  |  | 1 |
| B:33:_:PHE | VDW:SC_SC | B:60:_:HIS | 3.795 | NaN | 6 | CB | CB |  |  |  |  | 1 |
| B:33:_:PHE | VDW:SC_SC | B:285:_:ILE | 3.569 | NaN | 6 | CE1 | CG2 |  |  |  |  | 1 |
| B:33:_:PHE | VDW:SC_SC | B:289:_:LEU | 3.89 | NaN | 6 | CE2 | CD1 |  |  |  |  | 1 |
| B:34:_:ALA | HBOND:MC_MC | B:37:_:GLU | 3.378 | 51.346 | 17 | O | N | B:37:_:GLU |  |  |  | 1 |
| B:34:_:ALA | HBOND:MC_MC | B:38:_:LYS | 3.089 | 27.288 | 17 | O | N | B:38:_:LYS |  |  |  | 1 |
| B:35:_:ALA | HBOND:MC_MC | B:38:_:LYS | 3.3 | 40.759 | 17 | O | N | B:38:_:LYS |  |  |  | 1 |
| B:35:_:ALA | HBOND:MC_MC | B:39:_:ALA | 2.924 | 18.852 | 17 | O | N | B:39:_:ALA |  |  |  | 1 |
| B:36:_:LEU | HBOND:MC_MC | B:39:_:ALA | 3.321 | 51.083 | 17 | O | N | B:39:_:ALA |  |  |  | 1 |
| B:36:_:LEU | HBOND:MC_MC | B:40:_:CYS | 3.312 | 37.947 | 17 | O | N | B:40:_:CYS |  |  |  | 1 |
| B:36:_:LEU | VDW:SC_SC | B:281:_:GLN | 3.851 | NaN | 6 | CD2 | CB |  |  |  |  | 1 |
| B:36:_:LEU | VDW:SC_SC | B:282:_:VAL | 3.862 | NaN | 6 | CD2 | CG2 |  |  |  |  | 1 |
| B:37:_:GLU | HBOND:MC_MC | B:40:_:CYS | 3.364 | 23.074 | 17 | O | N | B:40:_:CYS |  |  |  | 1 |
| B:37:_:GLU | HBOND:MC_MC | B:41:_:ALA | 3.124 | 56.57 | 17 | O | N | B:41:_:ALA |  |  |  | 1 |
| B:37:_:GLU | HBOND:MC_MC | B:42:_:GLY | 3.12 | 4.06 | 17 | O | N | B:42:_:GLY |  |  |  | 1 |
| B:37:_:GLU | VDW:SC_MC | B:42:_:GLY | 3.825 | NaN | 6 | CG | C |  |  |  |  | 1 |
| B:37:_:GLU | HBOND:SC_MC | B:44:_:LEU | 2.776 | 4.022 | 17 | OE2 | N | B:44:_:LEU |  |  |  | 1 |
| B:37:_:GLU | IONIC:SC_SC | B:60:_:HIS | 3.457 | 106.666 | 20 | 91.285,103.599,66.112 | 88.261,105.102,66.849 |  | B:60:_:HIS |  |  | 1 |
| B:37:_:GLU | VDW:SC_SC | B:60:_:HIS | 3.223 | NaN | 6 | CD | CE1 |  |  |  |  | 1 |
| B:37:_:GLU | IONIC:SC_SC | B:61:_:ARG | 3.73 | 101.363 | 20 | 91.285,103.599,66.112 | CZ |  | B:61:_:ARG |  |  | 1 |
| B:38:_:LYS | HBOND:MC_MC | B:41:_:ALA | 3.052 | 18.424 | 17 | O | N | B:41:_:ALA |  |  |  | 1 |
| B:40:_:CYS | VDW:SC_SC | B:270:_:ILE | 3.648 | NaN | 6 | CB | CD1 |  |  |  |  | 1 |
| B:43:_:ARG | VDW:SC_SC | B:61:_:ARG | 3.715 | NaN | 6 | CG | CD |  |  |  |  | 1 |
| B:43:_:ARG | VDW:SC_SC | B:66:_:PHE | 3.477 | NaN | 6 | CZ | CE2 |  |  |  |  | 1 |
| B:43:_:ARG | HBOND:MC_MC | B:266:_:GLN | 2.879 | 13.497 | 17 | N | O | B:43:_:ARG |  |  |  | 1 |
| B:43:_:ARG | HBOND:MC_MC | B:266:_:GLN | 2.96 | 6.581 | 17 | O | N | B:266:_:GLN |  |  |  | 1 |
| B:43:_:ARG | VDW:SC_SC | B:266:_:GLN | 3.459 | NaN | 6 | CZ | NE2 |  |  |  |  | 1 |
| B:44:_:LEU | VDW:MC_SC | B:66:_:PHE | 3.804 | NaN | 6 | C | CZ |  |  |  |  | 1 |
| B:45:_:GLY | VDW:MC_SC | B:183:_:PRO | 3.43 | NaN | 6 | C | CG |  |  |  |  | 1 |
| B:45:_:GLY | HBOND:MC_MC | B:264:_:TYR | 2.758 | 8.578 | 17 | O | N | B:264:_:TYR |  |  |  | 1 |
| B:45:_:GLY | HBOND:MC_MC | B:264:_:TYR | 2.777 | 10.581 | 17 | N | O | B:45:_:GLY |  |  |  | 1 |
| B:46:_:VAL | HBOND:MC_MC | B:60:_:HIS | 2.901 | 12.396 | 17 | N | O | B:46:_:VAL |  |  |  | 1 |
| B:46:_:VAL | HBOND:MC_MC | B:60:_:HIS | 2.933 | 16.63 | 17 | O | N | B:60:_:HIS |  |  |  | 1 |
| B:46:_:VAL | VDW:MC_SC | B:183:_:PRO | 3.838 | NaN | 6 | C | CB |  |  |  |  | 1 |
| B:46:_:VAL | VDW:SC_SC | B:261:_:LEU | 3.833 | NaN | 6 | CG1 | CD1 |  |  |  |  | 1 |
| B:47:_:THR | VDW:SC_SC | B:56:_:ARG | 3.658 | NaN | 6 | CG2 | CD |  |  |  |  | 1 |
| B:47:_:THR | VDW:SC_SC | B:62:_:GLN | 3.445 | NaN | 6 | CG2 | OE1 |  |  |  |  | 1 |
| B:47:_:THR | VDW:SC_MC | B:183:_:PRO | 3.889 | NaN | 6 | CB | C |  |  |  |  | 1 |
| B:47:_:THR | VDW:SC_SC | B:187:_:ALA | 3.648 | NaN | 6 | CG2 | CB |  |  |  |  | 1 |
| B:47:_:THR | HBOND:MC_MC | B:262:_:THR | 2.887 | 10.475 | 17 | O | N | B:262:_:THR |  |  |  | 1 |
| B:47:_:THR | HBOND:MC_MC | B:262:_:THR | 3.074 | 15.764 | 17 | N | O | B:47:_:THR |  |  |  | 1 |
| B:48:_:LEU | HBOND:MC_MC | B:57:_:ILE | 2.793 | 10.417 | 17 | N | O | B:48:_:LEU |  |  |  | 1 |
| B:48:_:LEU | HBOND:MC_MC | B:57:_:ILE | 3.006 | 12.899 | 17 | O | N | B:57:_:ILE |  |  |  | 1 |
| B:48:_:LEU | VDW:SC_SC | B:259:_:TRP | 3.71 | NaN | 6 | CD1 | CE3 |  |  |  |  | 1 |
| B:49:_:LEU | VDW:SC_MC | B:54:_:GLY | 3.812 | NaN | 6 | CD1 | C |  |  |  |  | 1 |
| B:49:_:LEU | VDW:SC_SC | B:56:_:ARG | 3.712 | NaN | 6 | CD1 | CG |  |  |  |  | 1 |
| B:49:_:LEU | VDW:SC_SC | B:191:_:GLN | 3.421 | NaN | 6 | CD2 | OE1 |  |  |  |  | 1 |
| B:49:_:LEU | VDW:MC_SC | B:259:_:TRP | 3.759 | NaN | 6 | C | CE3 |  |  |  |  | 1 |
| B:49:_:LEU | HBOND:MC_MC | B:260:_:VAL | 2.77 | 0.705 | 17 | N | O | B:49:_:LEU |  |  |  | 1 |
| B:49:_:LEU | HBOND:MC_MC | B:260:_:VAL | 3.01 | 13.791 | 17 | O | N | B:260:_:VAL |  |  |  | 1 |
| B:50:_:ASP | HBOND:MC_MC | B:54:_:GLY | 2.799 | 31.433 | 17 | O | N | B:54:_:GLY |  |  |  | 1 |
| B:50:_:ASP | HBOND:MC_MC | B:55:_:ARG | 2.936 | 2.291 | 17 | N | O | B:50:_:ASP |  |  |  | 1 |
| B:50:_:ASP | VDW:SC_SC | B:55:_:ARG | 3.492 | NaN | 6 | CB | CG |  |  |  |  | 1 |
| B:50:_:ASP | VDW:SC_SC | B:259:_:TRP | 3.604 | NaN | 6 | CB | CZ3 |  |  |  |  | 1 |
| B:51:_:THR | VDW:SC_SC | B:191:_:GLN | 3.508 | NaN | 6 | CB | NE2 |  |  |  |  | 1 |
| B:51:_:THR | VDW:SC_SC | B:195:_:LEU | 3.735 | NaN | 6 | CG2 | CD1 |  |  |  |  | 1 |
| B:51:_:THR | VDW:SC_MC | B:258:_:PRO | 3.88 | NaN | 6 | CG2 | C |  |  |  |  | 1 |
| B:56:_:ARG | VDW:SC_SC | B:62:_:GLN | 3.594 | NaN | 6 | CB | OE1 |  |  |  |  | 1 |
| B:57:_:ILE | VDW:SC_SC | B:289:_:LEU | 3.84 | NaN | 6 | CG2 | CD1 |  |  |  |  | 1 |
| B:61:_:ARG | HBOND:MC_MC | B:64:_:GLU | 3.306 | 14.615 | 17 | O | N | B:64:_:GLU |  |  |  | 1 |
| B:61:_:ARG | VDW:SC_SC | B:64:_:GLU | 3.583 | NaN | 6 | CD | CD |  |  |  |  | 1 |
| B:62:_:GLN | VDW:MC_SC | B:183:_:PRO | 3.765 | NaN | 6 | C | CD |  |  |  |  | 1 |
| B:62:_:GLN | HBOND:MC_MC | B:184:_:ALA | 2.983 | 23.636 | 17 | O | N | B:184:_:ALA |  |  |  | 1 |
| B:63:_:ASP | VDW:MC_SC | B:182:_:THR | 3.646 | NaN | 6 | C | CG2 |  |  |  |  | 1 |
| B:64:_:GLU | VDW:MC_SC | B:182:_:THR | 3.649 | NaN | 6 | C | CG2 |  |  |  |  | 1 |
| B:66:_:PHE | HBOND:MC_MC | B:181:_:THR | 2.957 | 10.142 | 17 | N | O | B:66:_:PHE |  |  |  | 1 |
| B:66:_:PHE | HBOND:MC_MC | B:181:_:THR | 3.035 | 13.237 | 17 | O | N | B:181:_:THR |  |  |  | 1 |
| B:66:_:PHE | PIPISTACK:SC_SC | B:264:_:TYR | 6.074 | 143.549 | 9.4 | 92.576,109.446,59.906 | 89.742,110.594,54.658 |  |  |  | L n4.50,p4.29 | 1 |
| B:66:_:PHE | VDW:SC_SC | B:264:_:TYR | 3.481 | NaN | 6 | CG | CB |  |  |  |  | 1 |
| B:67:_:PRO | VDW:SC_SC | B:172:_:PHE | 3.595 | NaN | 6 | CD | CD1 |  |  |  |  | 1 |
| B:67:_:PRO | VDW:SC_SC | B:172:_:PHE | 3.702 | NaN | 6 | CB | CB |  |  |  |  | 1 |
| B:67:_:PRO | VDW:SC_SC | B:243:_:ALA | 3.807 | NaN | 6 | CG | CB |  |  |  |  | 1 |
| B:68:_:MET | HBOND:MC_MC | B:71:_:THR | 3.006 | 12.501 | 17 | O | N | B:71:_:THR |  |  |  | 1 |
| B:68:_:MET | VDW:SC_SC | B:72:_:PHE | 3.719 | NaN | 6 | SD | CB |  |  |  |  | 1 |
| B:68:_:MET | VDW:SC_MC | B:161:_:ARG | 3.804 | NaN | 6 | CE | C |  |  |  |  | 1 |
| B:68:_:MET | HBOND:MC_MC | B:179:_:ASP | 2.887 | 19.504 | 17 | N | O | B:68:_:MET |  |  |  | 1 |
| B:68:_:MET | VDW:SC_SC | B:181:_:THR | 3.601 | NaN | 6 | CG | CG2 |  |  |  |  | 1 |
| B:68:_:MET | VDW:SC_SC | B:186:_:MET | 3.608 | NaN | 6 | SD | CE |  |  |  |  | 1 |
| B:68:_:MET | VDW:MC_SC | B:245:_:ASN | 3.651 | NaN | 6 | C | ND2 |  |  |  |  | 1 |
| B:69:_:CYS | HBOND:MC_MC | B:72:_:PHE | 3.217 | 4.495 | 17 | O | N | B:72:_:PHE |  |  |  | 1 |
| B:69:_:CYS | VDW:SC_SC | B:170:_:ASN | 3.364 | NaN | 6 | CB | OD1 |  |  |  |  | 1 |
| B:69:_:CYS | VDW:SC_MC | B:236:_:GLY | 3.793 | NaN | 6 | CB | C |  |  |  |  | 1 |
| B:69:_:CYS | VDW:SC_MC | B:237:_:SER | 3.732 | NaN | 6 | SG | C |  |  |  |  | 1 |
| B:69:_:CYS | VDW:SC_SC | B:243:_:ALA | 3.945 | NaN | 6 | SG | CB |  |  |  |  | 1 |
| B:70:_:SER | HBOND:MC_MC | B:73:_:LYS | 2.892 | 11.525 | 17 | O | N | B:73:_:LYS |  |  |  | 1 |
| B:70:_:SER | VDW:SC_SC | B:73:_:LYS | 3.808 | NaN | 6 | CB | CD |  |  |  |  | 1 |
| B:71:_:THR | HBOND:MC_MC | B:74:_:SER | 3.215 | 8.808 | 17 | O | N | B:74:_:SER |  |  |  | 1 |
| B:71:_:THR | VDW:SC_MC | B:235:_:THR | 3.747 | NaN | 6 | CG2 | C |  |  |  |  | 1 |
| B:71:_:THR | VDW:SC_SC | B:245:_:ASN | 3.705 | NaN | 6 | CG2 | CB |  |  |  |  | 1 |
| B:71:_:THR | VDW:SC_SC | B:247:_:ILE | 3.823 | NaN | 6 | CB | CD1 |  |  |  |  | 1 |
| B:72:_:PHE | HBOND:MC_MC | B:75:_:MET | 2.846 | 24.174 | 17 | O | N | B:75:_:MET |  |  |  | 1 |
| B:72:_:PHE | HBOND:MC_MC | B:76:_:LEU | 3.016 | 28.548 | 17 | O | N | B:76:_:LEU |  |  |  | 1 |
| B:72:_:PHE | VDW:SC_SC | B:76:_:LEU | 3.773 | NaN | 6 | CE2 | CB |  |  |  |  | 1 |
| B:72:_:PHE | PIPISTACK:SC_SC | B:139:_:PHE | 5.394 | 11 | 9.4 | 94.003,115.880,44.811 | 96.801,119.316,41.735 |  |  |  | P n1.94,p5.00 | 1 |
| B:72:_:PHE | VDW:SC_SC | B:139:_:PHE | 3.3 | NaN | 6 | CE2 | CZ |  |  |  |  | 1 |
| B:72:_:PHE | VDW:SC_SC | B:169:_:LEU | 3.507 | NaN | 6 | CE1 | CD2 |  |  |  |  | 1 |
| B:72:_:PHE | VDW:SC_SC | B:169:_:LEU | 3.801 | NaN | 6 | CD1 | CD1 |  |  |  |  | 1 |
| B:73:_:LYS | HBOND:MC_MC | B:76:_:LEU | 3.273 | 41.867 | 17 | O | N | B:76:_:LEU |  |  |  | 1 |
| B:73:_:LYS | HBOND:MC_MC | B:77:_:ALA | 2.907 | 21.615 | 17 | O | N | B:77:_:ALA |  |  |  | 1 |
| B:73:_:LYS | VDW:MC_SC | B:127:_:ILE | 3.72 | NaN | 6 | C | CD1 |  |  |  |  | 1 |
| B:73:_:LYS | VDW:SC_SC | B:132:_:ASN | 3.415 | NaN | 6 | CE | OD1 |  |  |  |  | 1 |
| B:73:_:LYS | VDW:SC_SC | B:234:_:LYS | 3.847 | NaN | 6 | CD | CE |  |  |  |  | 1 |
| B:74:_:SER | HBOND:MC_MC | B:78:_:ALA | 3.104 | 38.085 | 17 | O | N | B:78:_:ALA |  |  |  | 1 |
| B:75:_:MET | HBOND:MC_MC | B:78:_:ALA | 3.415 | 42.414 | 17 | O | N | B:78:_:ALA |  |  |  | 1 |
| B:75:_:MET | HBOND:MC_MC | B:79:_:THR | 2.825 | 32.18 | 17 | O | N | B:79:_:THR |  |  |  | 1 |
| B:75:_:MET | VDW:SC_SC | B:152:_:LEU | 3.86 | NaN | 6 | CG | CD2 |  |  |  |  | 1 |
| B:75:_:MET | VDW:SC_SC | B:189:_:THR | 3.636 | NaN | 6 | SD | CG2 |  |  |  |  | 1 |
| B:76:_:LEU | HBOND:MC_MC | B:79:_:THR | 3.099 | 40.862 | 17 | O | N | B:79:_:THR |  |  |  | 1 |
| B:76:_:LEU | HBOND:MC_MC | B:80:_:VAL | 2.931 | 22.048 | 17 | O | N | B:80:_:VAL |  |  |  | 1 |
| B:76:_:LEU | VDW:SC_SC | B:135:_:ALA | 3.775 | NaN | 6 | CD2 | CB |  |  |  |  | 1 |
| B:76:_:LEU | VDW:SC_SC | B:148:_:VAL | 3.702 | NaN | 6 | CD1 | CG2 |  |  |  |  | 1 |
| B:77:_:ALA | HBOND:MC_MC | B:80:_:VAL | 3.205 | 44.646 | 17 | O | N | B:80:_:VAL |  |  |  | 1 |
| B:77:_:ALA | HBOND:MC_MC | B:81:_:LEU | 2.948 | 24.621 | 17 | O | N | B:81:_:LEU |  |  |  | 1 |
| B:77:_:ALA | VDW:SC_SC | B:127:_:ILE | 3.812 | NaN | 6 | CB | CD1 |  |  |  |  | 1 |
| B:78:_:ALA | HBOND:MC_MC | B:81:_:LEU | 3.248 | 43.462 | 17 | O | N | B:81:_:LEU |  |  |  | 1 |
| B:78:_:ALA | HBOND:MC_MC | B:82:_:SER | 2.859 | 24.268 | 17 | O | N | B:82:_:SER |  |  |  | 1 |
| B:78:_:ALA | VDW:SC_SC | B:151:_:PHE | 3.69 | NaN | 6 | CB | CE2 |  |  |  |  | 1 |
| B:78:_:ALA | VDW:SC_SC | B:193:_:VAL | 3.712 | NaN | 6 | CB | CG1 |  |  |  |  | 1 |
| B:78:_:ALA | VDW:SC_SC | B:207:_:LEU | 3.896 | NaN | 6 | CB | CD2 |  |  |  |  | 1 |
| B:79:_:THR | HBOND:MC_MC | B:82:_:SER | 3.127 | 44.291 | 17 | O | N | B:82:_:SER |  |  |  | 1 |
| B:79:_:THR | HBOND:MC_MC | B:83:_:GLN | 3.002 | 26.389 | 17 | O | N | B:83:_:GLN |  |  |  | 1 |
| B:80:_:VAL | HBOND:MC_MC | B:83:_:GLN | 3.22 | 37.377 | 17 | O | N | B:83:_:GLN |  |  |  | 1 |
| B:80:_:VAL | HBOND:MC_MC | B:84:_:ALA | 2.89 | 26.779 | 17 | O | N | B:84:_:ALA |  |  |  | 1 |
| B:80:_:VAL | VDW:SC_SC | B:119:_:VAL | 3.505 | NaN | 6 | CG1 | CG1 |  |  |  |  | 1 |
| B:80:_:VAL | VDW:SC_SC | B:123:_:CYS | 3.618 | NaN | 6 | CG1 | SG |  |  |  |  | 1 |
| B:80:_:VAL | VDW:SC_SC | B:142:_:VAL | 3.578 | NaN | 6 | CG2 | CG1 |  |  |  |  | 1 |
| B:81:_:LEU | HBOND:MC_MC | B:84:_:ALA | 3.216 | 41.587 | 17 | O | N | B:84:_:ALA |  |  |  | 1 |
| B:81:_:LEU | HBOND:MC_MC | B:85:_:GLU | 3.016 | 47.957 | 17 | O | N | B:85:_:GLU |  |  |  | 1 |
| B:81:_:LEU | VDW:SC_SC | B:207:_:LEU | 3.736 | NaN | 6 | CD1 | CB |  |  |  |  | 1 |
| B:81:_:LEU | VDW:SC_SC | B:210:_:TRP | 3.89 | NaN | 6 | CD1 | CZ3 |  |  |  |  | 1 |
| B:82:_:SER | HBOND:MC_MC | B:85:_:GLU | 3.424 | 21.323 | 17 | O | N | B:85:_:GLU |  |  |  | 1 |
| B:82:_:SER | VDW:SC_SC | B:151:_:PHE | 3.718 | NaN | 6 | CB | CE1 |  |  |  |  | 1 |
| B:82:_:SER | VDW:SC_SC | B:199:_:LEU | 3.852 | NaN | 6 | CB | CD2 |  |  |  |  | 1 |
| B:83:_:GLN | HBOND:MC_MC | B:86:_:ARG | 2.98 | 18.171 | 17 | O | N | B:86:_:ARG |  |  |  | 1 |
| B:83:_:GLN | HBOND:MC_MC | B:87:_:MET | 3.332 | 32.658 | 17 | O | N | B:87:_:MET |  |  |  | 1 |
| B:83:_:GLN | VDW:SC_SC | B:142:_:VAL | 3.395 | NaN | 6 | NE2 | CB |  |  |  |  | 1 |
| B:84:_:ALA | HBOND:MC_MC | B:87:_:MET | 3.077 | 37.604 | 17 | O | N | B:87:_:MET |  |  |  | 1 |
| B:84:_:ALA | VDW:MC_MC | B:87:_:MET | 3.848 | NaN | 6 | C | C |  |  |  |  | 1 |
| B:84:_:ALA | VDW:SC_SC | B:91:_:LEU | 3.659 | NaN | 6 | CB | CG |  |  |  |  | 1 |
| B:85:_:GLU | HBOND:SC_MC | B:200:_:GLN | 2.825 | 5.019 | 17 | OE2 | N | B:200:_:GLN |  |  |  | 1 |
| B:85:_:GLU | VDW:SC_SC | B:200:_:GLN | 3.544 | NaN | 6 | CG | OE1 |  |  |  |  | 1 |
| B:85:_:GLU | VDW:SC_SC | B:200:_:GLN | 3.565 | NaN | 6 | CD | CG |  |  |  |  | 1 |
| B:87:_:MET | HBOND:MC_MC | B:90:_:LEU | 2.623 | 24.156 | 17 | O | N | B:90:_:LEU |  |  |  | 1 |
| B:88:_:PRO | HBOND:MC_MC | B:91:_:LEU | 3.171 | 1.804 | 17 | O | N | B:91:_:LEU |  |  |  | 1 |
| B:89:_:ALA | HBOND:MC_MC | B:92:_:ASP | 2.961 | 30.439 | 17 | O | N | B:92:_:ASP |  |  |  | 1 |
| B:89:_:ALA | HBOND:MC_MC | B:93:_:ARG | 3.194 | 57.524 | 17 | O | N | B:93:_:ARG |  |  |  | 1 |
| B:90:_:LEU | HBOND:MC_MC | B:93:_:ARG | 3.108 | 12.408 | 17 | O | N | B:93:_:ARG |  |  |  | 1 |
| B:90:_:LEU | VDW:SC_SC | B:93:_:ARG | 3.592 | NaN | 6 | CD1 | CZ |  |  |  |  | 1 |
| B:90:_:LEU | VDW:SC_SC | B:141:_:VAL | 3.693 | NaN | 6 | CD1 | CG1 |  |  |  |  | 1 |
| B:91:_:LEU | HBOND:MC_MC | B:120:_:ARG | 2.901 | 26.995 | 17 | O | N | B:120:_:ARG |  |  |  | 1 |
| B:91:_:LEU | VDW:SC_SC | B:120:_:ARG | 3.766 | NaN | 6 | CB | CZ |  |  |  |  | 1 |
| B:93:_:ARG | HBOND:MC_MC | B:119:_:VAL | 2.928 | 24.069 | 17 | O | N | B:119:_:VAL |  |  |  | 1 |
| B:95:_:VAL | VDW:MC_SC | B:116:_:ASP | 3.709 | NaN | 6 | C | CB |  |  |  |  | 1 |
| B:95:_:VAL | HBOND:MC_MC | B:117:_:MET | 2.859 | 5.329 | 17 | N | O | B:95:_:VAL |  |  |  | 1 |
| B:95:_:VAL | HBOND:MC_MC | B:117:_:MET | 3.043 | 16.114 | 17 | O | N | B:117:_:MET |  |  |  | 1 |
| B:95:_:VAL | VDW:SC_SC | B:119:_:VAL | 3.813 | NaN | 6 | CG2 | CG2 |  |  |  |  | 1 |
| B:97:_:VAL | HBOND:MC_MC | B:114:_:GLY | 2.799 | 21.749 | 17 | O | N | B:114:_:GLY |  |  |  | 1 |
| B:97:_:VAL | HBOND:MC_MC | B:115:_:LYS | 2.852 | 2.365 | 17 | N | O | B:97:_:VAL |  |  |  | 1 |
| B:97:_:VAL | VDW:SC_SC | B:117:_:MET | 3.992 | NaN | 6 | CG2 | SD |  |  |  |  | 1 |
| B:97:_:VAL | VDW:SC_SC | B:137:_:LEU | 3.786 | NaN | 6 | CG2 | CD1 |  |  |  |  | 1 |
| B:98:_:GLY | HBOND:MC_SC | B:101:_:ASP | 2.798 | 26.426 | 17 | N | OD2 | B:98:_:GLY |  |  |  | 1 |
| B:98:_:GLY | HBOND:MC_MC | B:101:_:ASP | 2.886 | 14.152 | 17 | O | N | B:101:_:ASP |  |  |  | 1 |
| B:99:_:GLU | HBOND:MC_MC | B:102:_:LEU | 3.124 | 12.754 | 17 | O | N | B:102:_:LEU |  |  |  | 1 |
| B:99:_:GLU | VDW:SC_SC | B:102:_:LEU | 3.84 | NaN | 6 | CD | CD1 |  |  |  |  | 1 |
| B:101:_:ASP | VDW:MC_SC | B:133:_:THR | 3.845 | NaN | 6 | C | CG2 |  |  |  |  | 1 |
| B:101:_:ASP | VDW:SC_SC | B:137:_:LEU | 3.817 | NaN | 6 | CG | CD2 |  |  |  |  | 1 |
| B:102:_:LEU | VDW:SC_SC | B:113:_:ALA | 3.885 | NaN | 6 | CD1 | CB |  |  |  |  | 1 |
| B:103:_:LEU | VDW:SC_SC | B:132:_:ASN | 3.82 | NaN | 6 | CD1 | CB |  |  |  |  | 1 |
| B:103:_:LEU | HBOND:MC_SC | B:133:_:THR | 2.898 | 32.995 | 17 | N | OG1 | B:103:_:LEU |  |  |  | 1 |
| B:103:_:LEU | VDW:SC_SC | B:166:_:GLU | 3.769 | NaN | 6 | CD1 | CB |  |  |  |  | 1 |
| B:105:_:HIS | HBOND:MC_MC | B:132:_:ASN | 2.765 | 10.712 | 17 | O | N | B:132:_:ASN |  |  |  | 1 |
| B:106:_:ALA | HBOND:MC_MC | B:110:_:ARG | 2.969 | 29.652 | 17 | O | N | B:110:_:ARG |  |  |  | 1 |
| B:107:_:PRO | VDW:SC_SC | B:129:_:THR | 3.661 | NaN | 6 | CG | CB |  |  |  |  | 1 |
| B:108:_:VAL | HBOND:MC_MC | B:111:_:ARG | 2.986 | 11.601 | 17 | O | N | B:111:_:ARG |  |  |  | 1 |
| B:108:_:VAL | VDW:SC_SC | B:125:_:ALA | 3.772 | NaN | 6 | CB | CB |  |  |  |  | 1 |
| B:108:_:VAL | VDW:SC_SC | B:129:_:THR | 3.744 | NaN | 6 | CG2 | CG2 |  |  |  |  | 1 |
| B:108:_:VAL | HBOND:MC_SC | B:131:_:ASP | 3.006 | 20.222 | 17 | N | OD2 | B:108:_:VAL |  |  |  | 1 |
| B:109:_:THR | HBOND:MC_MC | B:112:_:HIS | 2.884 | 14.935 | 17 | O | N | B:112:_:HIS |  |  |  | 1 |
| B:109:_:THR | VDW:SC_SC | B:117:_:MET | 3.758 | NaN | 6 | CG2 | SD |  |  |  |  | 1 |
| B:109:_:THR | VDW:SC_SC | B:122:_:LEU | 3.867 | NaN | 6 | CG2 | CD2 |  |  |  |  | 1 |
| B:109:_:THR | HBOND:MC_SC | B:131:_:ASP | 2.796 | 14.161 | 17 | N | OD2 | B:109:_:THR |  |  |  | 1 |
| B:109:_:THR | VDW:SC_SC | B:131:_:ASP | 3.81 | NaN | 6 | CG2 | CG |  |  |  |  | 1 |
| B:109:_:THR | VDW:SC_SC | B:133:_:THR | 3.86 | NaN | 6 | CB | CG2 |  |  |  |  | 1 |
| B:112:_:HIS | HBOND:MC_MC | B:115:_:LYS | 2.993 | 18.926 | 17 | O | N | B:115:_:LYS |  |  |  | 1 |
| B:112:_:HIS | VDW:MC_SC | B:115:_:LYS | 3.626 | NaN | 6 | C | CG |  |  |  |  | 1 |
| B:112:_:HIS | VDW:SC_SC | B:117:_:MET | 3.649 | NaN | 6 | CG | CG |  |  |  |  | 1 |
| B:112:_:HIS | VDW:SC_SC | B:117:_:MET | 3.753 | NaN | 6 | CD2 | SD |  |  |  |  | 1 |
| B:117:_:MET | VDW:SC_SC | B:122:_:LEU | 3.678 | NaN | 6 | CE | CD2 |  |  |  |  | 1 |
| B:118:_:THR | HBOND:SC_MC | B:121:_:ASP | 2.929 | 31.919 | 17 | OG1 | N | B:121:_:ASP |  |  |  | 1 |
| B:118:_:THR | HBOND:MC_MC | B:121:_:ASP | 3.04 | 32.643 | 17 | O | N | B:121:_:ASP |  |  |  | 1 |
| B:118:_:THR | HBOND:MC_MC | B:122:_:LEU | 2.878 | 31.082 | 17 | O | N | B:122:_:LEU |  |  |  | 1 |
| B:119:_:VAL | HBOND:MC_MC | B:122:_:LEU | 3.155 | 36.804 | 17 | O | N | B:122:_:LEU |  |  |  | 1 |
| B:119:_:VAL | HBOND:MC_MC | B:123:_:CYS | 2.894 | 31.33 | 17 | O | N | B:123:_:CYS |  |  |  | 1 |
| B:119:_:VAL | VDW:SC_SC | B:138:_:LEU | 3.801 | NaN | 6 | CG1 | CD2 |  |  |  |  | 1 |
| B:120:_:ARG | HBOND:MC_MC | B:123:_:CYS | 3.218 | 37.298 | 17 | O | N | B:123:_:CYS |  |  |  | 1 |
| B:120:_:ARG | HBOND:MC_MC | B:124:_:ARG | 3.061 | 24.6 | 17 | O | N | B:124:_:ARG |  |  |  | 1 |
| B:121:_:ASP | HBOND:MC_MC | B:124:_:ARG | 3.472 | 43.863 | 17 | O | N | B:124:_:ARG |  |  |  | 1 |
| B:121:_:ASP | HBOND:MC_MC | B:125:_:ALA | 2.977 | 24.227 | 17 | O | N | B:125:_:ALA |  |  |  | 1 |
| B:122:_:LEU | HBOND:MC_MC | B:125:_:ALA | 3.315 | 45.625 | 17 | O | N | B:125:_:ALA |  |  |  | 1 |
| B:122:_:LEU | HBOND:MC_MC | B:126:_:THR | 2.982 | 36.205 | 17 | O | N | B:126:_:THR |  |  |  | 1 |
| B:122:_:LEU | VDW:SC_SC | B:134:_:ALA | 3.877 | NaN | 6 | CD2 | CB |  |  |  |  | 1 |
| B:122:_:LEU | VDW:SC_SC | B:138:_:LEU | 3.816 | NaN | 6 | CB | CD2 |  |  |  |  | 1 |
| B:122:_:LEU | VDW:MC_SC | B:138:_:LEU | 3.863 | NaN | 6 | C | CD1 |  |  |  |  | 1 |
| B:123:_:CYS | HBOND:MC_MC | B:126:_:THR | 3.233 | 28.546 | 17 | O | N | B:126:_:THR |  |  |  | 1 |
| B:123:_:CYS | HBOND:MC_MC | B:127:_:ILE | 3.088 | 36.994 | 17 | O | N | B:127:_:ILE |  |  |  | 1 |
| B:123:_:CYS | VDW:SC_SC | B:138:_:LEU | 3.999 | NaN | 6 | SG | CD1 |  |  |  |  | 1 |
| B:123:_:CYS | VDW:SC_SC | B:210:_:TRP | 3.497 | NaN | 6 | CB | CZ3 |  |  |  |  | 1 |
| B:124:_:ARG | HBOND:MC_MC | B:127:_:ILE | 3.14 | 27.185 | 17 | O | N | B:127:_:ILE |  |  |  | 1 |
| B:124:_:ARG | HBOND:MC_MC | B:128:_:ILE | 2.958 | 31.62 | 17 | O | N | B:128:_:ILE |  |  |  | 1 |
| B:124:_:ARG | VDW:SC_SC | B:210:_:TRP | 3.899 | NaN | 6 | CG | CD1 |  |  |  |  | 1 |
| B:125:_:ALA | HBOND:MC_MC | B:129:_:THR | 3.141 | 25.643 | 17 | O | N | B:129:_:THR |  |  |  | 1 |
| B:126:_:THR | VDW:SC_SC | B:134:_:ALA | 3.834 | NaN | 6 | CB | CB |  |  |  |  | 1 |
| B:127:_:ILE | VDW:SC_MC | B:210:_:TRP | 3.748 | NaN | 6 | CG2 | C |  |  |  |  | 1 |
| B:128:_:ILE | VDW:SC_MC | B:213:_:ASP | 3.741 | NaN | 6 | CG2 | C |  |  |  |  | 1 |
| B:128:_:ILE | VDW:MC_SC | B:214:_:ASN | 3.511 | NaN | 6 | C | OD1 |  |  |  |  | 1 |
| B:128:_:ILE | HBOND:MC_MC | B:215:_:GLU | 2.975 | 21.754 | 17 | O | N | B:215:_:GLU |  |  |  | 1 |
| B:129:_:THR | VDW:MC_SC | B:216:_:THR | 3.584 | NaN | 6 | C | CG2 |  |  |  |  | 1 |
| B:131:_:ASP | HBOND:SC_MC | B:134:_:ALA | 2.819 | 23.298 | 17 | OD1 | N | B:134:_:ALA |  |  |  | 1 |
| B:131:_:ASP | VDW:SC_SC | B:134:_:ALA | 3.822 | NaN | 6 | CG | CB |  |  |  |  | 1 |
| B:132:_:ASN | HBOND:MC_MC | B:135:_:ALA | 3.158 | 33.209 | 17 | O | N | B:135:_:ALA |  |  |  | 1 |
| B:132:_:ASN | HBOND:MC_MC | B:136:_:ASN | 3.112 | 31.135 | 17 | O | N | B:136:_:ASN |  |  |  | 1 |
| B:132:_:ASN | VDW:SC_SC | B:166:_:GLU | 3.28 | NaN | 6 | OD1 | CD |  |  |  |  | 1 |
| B:133:_:THR | HBOND:MC_MC | B:136:_:ASN | 3.116 | 34.557 | 17 | O | N | B:136:_:ASN |  |  |  | 1 |
| B:133:_:THR | HBOND:MC_MC | B:137:_:LEU | 2.986 | 29.995 | 17 | O | N | B:137:_:LEU |  |  |  | 1 |
| B:134:_:ALA | HBOND:MC_MC | B:137:_:LEU | 3.273 | 35.645 | 17 | O | N | B:137:_:LEU |  |  |  | 1 |
| B:134:_:ALA | HBOND:MC_MC | B:138:_:LEU | 2.949 | 29.763 | 17 | O | N | B:138:_:LEU |  |  |  | 1 |
| B:135:_:ALA | HBOND:MC_MC | B:138:_:LEU | 3.184 | 38.866 | 17 | O | N | B:138:_:LEU |  |  |  | 1 |
| B:135:_:ALA | HBOND:MC_MC | B:139:_:PHE | 2.84 | 28.622 | 17 | O | N | B:139:_:PHE |  |  |  | 1 |
| B:136:_:ASN | HBOND:MC_MC | B:139:_:PHE | 3.253 | 40.445 | 17 | O | N | B:139:_:PHE |  |  |  | 1 |
| B:136:_:ASN | VDW:SC_SC | B:139:_:PHE | 3.646 | NaN | 6 | OD1 | CD2 |  |  |  |  | 1 |
| B:136:_:ASN | HBOND:MC_MC | B:140:_:GLY | 3.155 | 33.735 | 17 | O | N | B:140:_:GLY |  |  |  | 1 |
| B:136:_:ASN | VDW:SC_SC | B:165:_:LEU | 3.575 | NaN | 6 | OD1 | CB |  |  |  |  | 1 |
| B:136:_:ASN | HBOND:SC_MC | B:166:_:GLU | 2.863 | 7.81 | 17 | OD1 | N | B:166:_:GLU |  |  |  | 1 |
| B:136:_:ASN | VDW:SC_SC | B:166:_:GLU | 3.403 | NaN | 6 | OD1 | CB |  |  |  |  | 1 |
| B:137:_:LEU | HBOND:MC_MC | B:140:_:GLY | 3.244 | 32.483 | 17 | O | N | B:140:_:GLY |  |  |  | 1 |
| B:137:_:LEU | HBOND:MC_MC | B:141:_:VAL | 3.281 | 35.451 | 17 | O | N | B:141:_:VAL |  |  |  | 1 |
| B:138:_:LEU | HBOND:MC_MC | B:141:_:VAL | 2.979 | 33.697 | 17 | O | N | B:141:_:VAL |  |  |  | 1 |
| B:138:_:LEU | HBOND:MC_MC | B:142:_:VAL | 3.041 | 35.628 | 17 | O | N | B:142:_:VAL |  |  |  | 1 |
| B:139:_:PHE | HBOND:MC_MC | B:142:_:VAL | 3.278 | 26.199 | 17 | O | N | B:142:_:VAL |  |  |  | 1 |
| B:139:_:PHE | HBOND:MC_MC | B:143:_:GLY | 3.114 | 45.113 | 17 | O | N | B:143:_:GLY |  |  |  | 1 |
| B:139:_:PHE | HBOND:MC_MC | B:144:_:GLY | 2.79 | 14.149 | 17 | O | N | B:144:_:GLY |  |  |  | 1 |
| B:139:_:PHE | VDW:SC_MC | B:144:_:GLY | 3.49 | NaN | 6 | CD1 | C |  |  |  |  | 1 |
| B:139:_:PHE | VDW:SC_SC | B:145:_:PRO | 3.728 | NaN | 6 | CD1 | CD |  |  |  |  | 1 |
| B:139:_:PHE | VDW:SC_SC | B:145:_:PRO | 3.801 | NaN | 6 | CE1 | CG |  |  |  |  | 1 |
| B:139:_:PHE | VDW:SC_SC | B:165:_:LEU | 3.525 | NaN | 6 | CD2 | CD2 |  |  |  |  | 1 |
| B:142:_:VAL | VDW:MC_SC | B:147:_:ALA | 3.771 | NaN | 6 | C | CB |  |  |  |  | 1 |
| B:144:_:GLY | HBOND:MC_MC | B:147:_:ALA | 2.881 | 17.093 | 17 | O | N | B:147:_:ALA |  |  |  | 1 |
| B:144:_:GLY | HBOND:MC_MC | B:148:_:VAL | 3.453 | 28.589 | 17 | O | N | B:148:_:VAL |  |  |  | 1 |
| B:145:_:PRO | HBOND:MC_MC | B:148:_:VAL | 3.144 | 33.716 | 17 | O | N | B:148:_:VAL |  |  |  | 1 |
| B:145:_:PRO | HBOND:MC_MC | B:149:_:THR | 3.025 | 15.303 | 17 | O | N | B:149:_:THR |  |  |  | 1 |
| B:145:_:PRO | VDW:SC_MC | B:162:_:SER | 3.64 | NaN | 6 | CB | C |  |  |  |  | 1 |
| B:145:_:PRO | VDW:SC_MC | B:163:_:ASP | 3.44 | NaN | 6 | CB | C |  |  |  |  | 1 |
| B:145:_:PRO | VDW:SC_MC | B:164:_:ARG | 3.693 | NaN | 6 | CG | C |  |  |  |  | 1 |
| B:145:_:PRO | VDW:SC_SC | B:165:_:LEU | 3.803 | NaN | 6 | CD | CD2 |  |  |  |  | 1 |
| B:146:_:PRO | HBOND:MC_MC | B:149:_:THR | 3.443 | 49.439 | 17 | O | N | B:149:_:THR |  |  |  | 1 |
| B:146:_:PRO | HBOND:MC_MC | B:150:_:ALA | 2.882 | 23.943 | 17 | O | N | B:150:_:ALA |  |  |  | 1 |
| B:147:_:ALA | HBOND:MC_MC | B:150:_:ALA | 3.489 | 48.896 | 17 | O | N | B:150:_:ALA |  |  |  | 1 |
| B:147:_:ALA | HBOND:MC_MC | B:151:_:PHE | 2.979 | 29.151 | 17 | O | N | B:151:_:PHE |  |  |  | 1 |
| B:148:_:VAL | HBOND:MC_MC | B:151:_:PHE | 3.239 | 39.872 | 17 | O | N | B:151:_:PHE |  |  |  | 1 |
| B:148:_:VAL | HBOND:MC_MC | B:152:_:LEU | 2.949 | 15.391 | 17 | O | N | B:152:_:LEU |  |  |  | 1 |
| B:148:_:VAL | VDW:SC_SC | B:162:_:SER | 3.817 | NaN | 6 | CB | CB |  |  |  |  | 1 |
| B:149:_:THR | HBOND:MC_MC | B:152:_:LEU | 3.109 | 53.343 | 17 | O | N | B:152:_:LEU |  |  |  | 1 |
| B:149:_:THR | HBOND:MC_MC | B:153:_:ARG | 2.858 | 27.291 | 17 | O | N | B:153:_:ARG |  |  |  | 1 |
| B:149:_:THR | VDW:SC_SC | B:153:_:ARG | 3.872 | NaN | 6 | CG2 | CZ |  |  |  |  | 1 |
| B:149:_:THR | HBOND:SC_MC | B:162:_:SER | 2.941 | 21.124 | 17 | OG1 | N | B:162:_:SER |  |  |  | 1 |
| B:150:_:ALA | HBOND:MC_MC | B:153:_:ARG | 3.344 | 40.051 | 17 | O | N | B:153:_:ARG |  |  |  | 1 |
| B:150:_:ALA | HBOND:MC_MC | B:154:_:ALA | 2.931 | 27.598 | 17 | O | N | B:154:_:ALA |  |  |  | 1 |
| B:151:_:PHE | HBOND:MC_MC | B:154:_:ALA | 3.216 | 40.188 | 17 | O | N | B:154:_:ALA |  |  |  | 1 |
| B:151:_:PHE | HBOND:MC_MC | B:155:_:SER | 2.903 | 24.369 | 17 | O | N | B:155:_:SER |  |  |  | 1 |
| B:151:_:PHE | VDW:SC_SC | B:198:_:VAL | 3.77 | NaN | 6 | CZ | CG1 |  |  |  |  | 1 |
| B:152:_:LEU | HBOND:MC_MC | B:155:_:SER | 3.155 | 44.569 | 17 | O | N | B:155:_:SER |  |  |  | 1 |
| B:152:_:LEU | HBOND:MC_MC | B:156:_:GLY | 3.179 | 48.452 | 17 | O | N | B:156:_:GLY |  |  |  | 1 |
| B:152:_:LEU | HBOND:MC_MC | B:157:_:ASP | 2.975 | 12.904 | 17 | O | N | B:157:_:ASP |  |  |  | 1 |
| B:152:_:LEU | VDW:SC_SC | B:160:_:SER | 3.87 | NaN | 6 | CD1 | CB |  |  |  |  | 1 |
| B:153:_:ARG | HBOND:MC_MC | B:156:_:GLY | 2.964 | 20.868 | 17 | O | N | B:156:_:GLY |  |  |  | 1 |
| B:157:_:ASP | HBOND:SC_MC | B:160:_:SER | 2.828 | 4.671 | 17 | OD1 | N | B:160:_:SER |  |  |  | 1 |
| B:157:_:ASP | VDW:SC_SC | B:160:_:SER | 3.655 | NaN | 6 | CG | CB |  |  |  |  | 1 |
| B:159:_:VAL | VDW:SC_SC | B:182:_:THR | 3.648 | NaN | 6 | CG1 | CG2 |  |  |  |  | 1 |
| B:159:_:VAL | VDW:SC_SC | B:185:_:ALA | 3.519 | NaN | 6 | CB | CB |  |  |  |  | 1 |
| B:161:_:ARG | HBOND:MC_MC | B:179:_:ASP | 3.378 | 59.07 | 17 | O | N | B:179:_:ASP |  |  |  | 1 |
| B:161:_:ARG | HBOND:MC_MC | B:180:_:THR | 3.094 | 33.562 | 17 | N | O | B:161:_:ARG |  |  |  | 1 |
| B:163:_:ASP | VDW:SC_SC | B:178:_:ARG | 3.845 | NaN | 6 | CB | CB |  |  |  |  | 1 |
| B:163:_:ASP | VDW:SC_SC | B:178:_:ARG | 3.888 | NaN | 6 | CG | CG |  |  |  |  | 1 |
| B:163:_:ASP | HBOND:MC_SC | B:179:_:ASP | 2.738 | 14.826 | 17 | N | OD1 | B:163:_:ASP |  |  |  | 1 |
| B:163:_:ASP | HBOND:SC_MC | B:179:_:ASP | 2.898 | 10.817 | 17 | OD2 | N | B:179:_:ASP |  |  |  | 1 |
| B:164:_:ARG | VDW:SC_SC | B:169:_:LEU | 3.857 | NaN | 6 | CB | CB |  |  |  |  | 1 |
| B:164:_:ARG | HBOND:MC_SC | B:179:_:ASP | 3.149 | 17.113 | 17 | N | OD1 | B:164:_:ARG |  |  |  | 1 |
| B:165:_:LEU | HBOND:MC_MC | B:168:_:GLU | 3.247 | 57.074 | 17 | O | N | B:168:_:GLU |  |  |  | 1 |
| B:165:_:LEU | HBOND:MC_MC | B:169:_:LEU | 2.939 | 39.52 | 17 | O | N | B:169:_:LEU |  |  |  | 1 |
| B:165:_:LEU | VDW:MC_SC | B:169:_:LEU | 3.642 | NaN | 6 | C | CD2 |  |  |  |  | 1 |
| B:166:_:GLU | VDW:SC_SC | B:169:_:LEU | 3.539 | NaN | 6 | CD | CD2 |  |  |  |  | 1 |
| B:167:_:PRO | HBOND:MC_MC | B:170:_:ASN | 3.123 | 7.717 | 17 | O | N | B:170:_:ASN |  |  |  | 1 |
| B:168:_:GLU | HBOND:MC_MC | B:171:_:SER | 3.032 | 12.158 | 17 | O | N | B:171:_:SER |  |  |  | 1 |
| B:169:_:LEU | VDW:SC_SC | B:179:_:ASP | 3.676 | NaN | 6 | CB | CG |  |  |  |  | 1 |
| B:172:_:PHE | VDW:SC_SC | B:180:_:THR | 3.868 | NaN | 6 | CE1 | CG2 |  |  |  |  | 1 |
| B:172:_:PHE | VDW:SC_SC | B:266:_:GLN | 3.333 | NaN | 6 | CE2 | NE2 |  |  |  |  | 1 |
| B:172:_:PHE | VDW:SC_SC | B:266:_:GLN | 3.537 | NaN | 6 | CD2 | OE1 |  |  |  |  | 1 |
| B:173:_:ALA | HBOND:MC_MC | B:176:_:ASP | 2.932 | 23.352 | 17 | O | N | B:176:_:ASP |  |  |  | 1 |
| B:177:_:PRO | HBOND:MC_MC | B:180:_:THR | 3.315 | 46.454 | 17 | O | N | B:180:_:THR |  |  |  | 1 |
| B:177:_:PRO | VDW:MC_SC | B:180:_:THR | 3.693 | NaN | 6 | C | CG2 |  |  |  |  | 1 |
| B:181:_:THR | VDW:SC_SC | B:264:_:TYR | 3.538 | NaN | 6 | CG2 | CZ |  |  |  |  | 1 |
| B:182:_:THR | HBOND:SC_MC | B:185:_:ALA | 2.957 | 24.31 | 17 | OG1 | N | B:185:_:ALA |  |  |  | 1 |
| B:182:_:THR | HBOND:MC_MC | B:185:_:ALA | 3.368 | 37.385 | 17 | O | N | B:185:_:ALA |  |  |  | 1 |
| B:182:_:THR | HBOND:MC_MC | B:186:_:MET | 3.009 | 26.34 | 17 | O | N | B:186:_:MET |  |  |  | 1 |
| B:183:_:PRO | HBOND:MC_MC | B:187:_:ALA | 3.021 | 33.309 | 17 | O | N | B:187:_:ALA |  |  |  | 1 |
| B:184:_:ALA | HBOND:MC_MC | B:187:_:ALA | 3.309 | 37.218 | 17 | O | N | B:187:_:ALA |  |  |  | 1 |
| B:184:_:ALA | HBOND:MC_MC | B:188:_:ALA | 3.039 | 22.361 | 17 | O | N | B:188:_:ALA |  |  |  | 1 |
| B:185:_:ALA | HBOND:MC_MC | B:188:_:ALA | 3.283 | 41.437 | 17 | O | N | B:188:_:ALA |  |  |  | 1 |
| B:185:_:ALA | HBOND:MC_MC | B:189:_:THR | 2.938 | 21.573 | 17 | O | N | B:189:_:THR |  |  |  | 1 |
| B:186:_:MET | HBOND:MC_MC | B:189:_:THR | 3.326 | 47.923 | 17 | O | N | B:189:_:THR |  |  |  | 1 |
| B:186:_:MET | HBOND:MC_MC | B:190:_:LEU | 2.817 | 30.54 | 17 | O | N | B:190:_:LEU |  |  |  | 1 |
| B:186:_:MET | VDW:SC_SC | B:247:_:ILE | 3.674 | NaN | 6 | CG | CD1 |  |  |  |  | 1 |
| B:187:_:ALA | HBOND:MC_MC | B:190:_:LEU | 3.33 | 35.665 | 17 | O | N | B:190:_:LEU |  |  |  | 1 |
| B:187:_:ALA | HBOND:MC_MC | B:191:_:GLN | 3.034 | 31.163 | 17 | O | N | B:191:_:GLN |  |  |  | 1 |
| B:187:_:ALA | VDW:SC_SC | B:262:_:THR | 3.833 | NaN | 6 | CB | CG2 |  |  |  |  | 1 |
| B:188:_:ALA | HBOND:MC_MC | B:191:_:GLN | 3.215 | 36.517 | 17 | O | N | B:191:_:GLN |  |  |  | 1 |
| B:188:_:ALA | HBOND:MC_MC | B:192:_:ARG | 2.919 | 22.436 | 17 | O | N | B:192:_:ARG |  |  |  | 1 |
| B:189:_:THR | HBOND:MC_MC | B:192:_:ARG | 3.249 | 46.63 | 17 | O | N | B:192:_:ARG |  |  |  | 1 |
| B:189:_:THR | HBOND:MC_MC | B:193:_:VAL | 2.87 | 16.526 | 17 | O | N | B:193:_:VAL |  |  |  | 1 |
| B:190:_:LEU | HBOND:MC_MC | B:194:_:VAL | 2.82 | 19.293 | 17 | O | N | B:194:_:VAL |  |  |  | 1 |
| B:190:_:LEU | VDW:SC_SC | B:194:_:VAL | 3.871 | NaN | 6 | CD2 | CG2 |  |  |  |  | 1 |
| B:190:_:LEU | VDW:SC_SC | B:247:_:ILE | 3.823 | NaN | 6 | CD1 | CB |  |  |  |  | 1 |
| B:191:_:GLN | HBOND:MC_MC | B:195:_:LEU | 2.981 | 30.034 | 17 | O | N | B:195:_:LEU |  |  |  | 1 |
| B:191:_:GLN | HBOND:MC_MC | B:196:_:GLY | 2.804 | 36.949 | 17 | O | N | B:196:_:GLY |  |  |  | 1 |
| B:192:_:ARG | HBOND:MC_MC | B:196:_:GLY | 3.281 | 47.285 | 17 | O | N | B:196:_:GLY |  |  |  | 1 |
| B:192:_:ARG | HBOND:MC_MC | B:198:_:VAL | 3.487 | 14.514 | 17 | O | N | B:198:_:VAL |  |  |  | 1 |
| B:192:_:ARG | VDW:MC_SC | B:198:_:VAL | 3.796 | NaN | 6 | C | CG2 |  |  |  |  | 1 |
| B:193:_:VAL | VDW:SC_SC | B:199:_:LEU | 3.874 | NaN | 6 | CG1 | CD1 |  |  |  |  | 1 |
| B:193:_:VAL | VDW:SC_SC | B:207:_:LEU | 3.778 | NaN | 6 | CG1 | CD2 |  |  |  |  | 1 |
| B:194:_:VAL | VDW:MC_SC | B:204:_:ARG | 3.644 | NaN | 6 | C | CD |  |  |  |  | 1 |
| B:194:_:VAL | VDW:SC_SC | B:211:_:LEU | 3.796 | NaN | 6 | CG2 | CD1 |  |  |  |  | 1 |
| B:195:_:LEU | VDW:MC_SC | B:204:_:ARG | 3.685 | NaN | 6 | C | CD |  |  |  |  | 1 |
| B:195:_:LEU | VDW:SC_SC | B:258:_:PRO | 3.861 | NaN | 6 | CD1 | CB |  |  |  |  | 1 |
| B:199:_:LEU | VDW:SC_SC | B:203:_:SER | 3.782 | NaN | 6 | CB | CB |  |  |  |  | 1 |
| B:200:_:GLN | HBOND:MC_MC | B:203:_:SER | 2.98 | 32.318 | 17 | O | N | B:203:_:SER |  |  |  | 1 |
| B:200:_:GLN | HBOND:MC_MC | B:204:_:ARG | 2.962 | 4.641 | 17 | O | N | B:204:_:ARG |  |  |  | 1 |
| B:201:_:PRO | HBOND:MC_MC | B:204:_:ARG | 3.238 | 59.342 | 17 | O | N | B:204:_:ARG |  |  |  | 1 |
| B:201:_:PRO | HBOND:MC_MC | B:205:_:GLN | 2.881 | 25.053 | 17 | O | N | B:205:_:GLN |  |  |  | 1 |
| B:202:_:ALA | HBOND:MC_MC | B:206:_:GLN | 3.042 | 36.575 | 17 | O | N | B:206:_:GLN |  |  |  | 1 |
| B:203:_:SER | HBOND:MC_MC | B:206:_:GLN | 3.15 | 34.46 | 17 | O | N | B:206:_:GLN |  |  |  | 1 |
| B:203:_:SER | HBOND:MC_MC | B:207:_:LEU | 2.973 | 20.929 | 17 | O | N | B:207:_:LEU |  |  |  | 1 |
| B:204:_:ARG | HBOND:MC_MC | B:207:_:LEU | 3.269 | 44.362 | 17 | O | N | B:207:_:LEU |  |  |  | 1 |
| B:204:_:ARG | HBOND:MC_MC | B:208:_:ALA | 2.877 | 22.722 | 17 | O | N | B:208:_:ALA |  |  |  | 1 |
| B:205:_:GLN | HBOND:MC_MC | B:208:_:ALA | 3.39 | 46.2 | 17 | O | N | B:208:_:ALA |  |  |  | 1 |
| B:205:_:GLN | HBOND:MC_MC | B:209:_:ASP | 2.936 | 33.407 | 17 | O | N | B:209:_:ASP |  |  |  | 1 |
| B:206:_:GLN | HBOND:MC_MC | B:209:_:ASP | 3.241 | 35.329 | 17 | O | N | B:209:_:ASP |  |  |  | 1 |
| B:206:_:GLN | HBOND:MC_MC | B:210:_:TRP | 3.071 | 29.897 | 17 | O | N | B:210:_:TRP |  |  |  | 1 |
| B:206:_:GLN | VDW:SC_SC | B:210:_:TRP | 3.421 | NaN | 6 | CG | CZ2 |  |  |  |  | 1 |
| B:206:_:GLN | VDW:SC_SC | B:210:_:TRP | 3.574 | NaN | 6 | OE1 | CE2 |  |  |  |  | 1 |
| B:207:_:LEU | HBOND:MC_MC | B:210:_:TRP | 3.167 | 37.596 | 17 | O | N | B:210:_:TRP |  |  |  | 1 |
| B:207:_:LEU | HBOND:MC_MC | B:211:_:LEU | 3.006 | 25.513 | 17 | O | N | B:211:_:LEU |  |  |  | 1 |
| B:208:_:ALA | HBOND:MC_MC | B:211:_:LEU | 3.168 | 40.431 | 17 | O | N | B:211:_:LEU |  |  |  | 1 |
| B:208:_:ALA | HBOND:MC_MC | B:212:_:ILE | 2.848 | 25.159 | 17 | O | N | B:212:_:ILE |  |  |  | 1 |
| B:209:_:ASP | HBOND:MC_MC | B:212:_:ILE | 3.227 | 44.282 | 17 | O | N | B:212:_:ILE |  |  |  | 1 |
| B:209:_:ASP | HBOND:MC_MC | B:213:_:ASP | 2.958 | 27.757 | 17 | O | N | B:213:_:ASP |  |  |  | 1 |
| B:210:_:TRP | HBOND:MC_MC | B:213:_:ASP | 3.1 | 40.403 | 17 | O | N | B:213:_:ASP |  |  |  | 1 |
| B:211:_:LEU | HBOND:MC_MC | B:214:_:ASN | 3.194 | 24.019 | 17 | O | N | B:214:_:ASN |  |  |  | 1 |
| B:211:_:LEU | VDW:SC_MC | B:232:_:GLY | 3.851 | NaN | 6 | CB | C |  |  |  |  | 1 |
| B:211:_:LEU | HBOND:MC_MC | B:233:_:ASP | 3.023 | 18.597 | 17 | O | N | B:233:_:ASP |  |  |  | 1 |
| B:211:_:LEU | VDW:SC_MC | B:233:_:ASP | 3.747 | NaN | 6 | CD2 | C |  |  |  |  | 1 |
| B:212:_:ILE | VDW:SC_SC | B:230:_:ARG | 3.727 | NaN | 6 | CD1 | CD |  |  |  |  | 1 |
| B:212:_:ILE | VDW:SC_SC | B:251:_:TRP | 3.824 | NaN | 6 | CG1 | CZ3 |  |  |  |  | 1 |
| B:214:_:ASN | VDW:SC_SC | B:233:_:ASP | 3.32 | NaN | 6 | ND2 | CG |  |  |  |  | 1 |
| B:217:_:GLY | HBOND:MC_MC | B:220:_:CYS | 3.042 | 11.08 | 17 | O | N | B:220:_:CYS |  |  |  | 1 |
| B:219:_:ALA | VDW:MC_SC | B:223:_:ALA | 3.869 | NaN | 6 | C | CB |  |  |  |  | 1 |
| B:220:_:CYS | HBOND:MC_MC | B:223:_:ALA | 2.938 | 15.855 | 17 | O | N | B:223:_:ALA |  |  |  | 1 |
| B:220:_:CYS | VDW:SC_SC | B:279:_:LEU | 3.929 | NaN | 6 | SG | CD1 |  |  |  |  | 1 |
| B:221:_:LEU | HBOND:MC_MC | B:224:_:GLY | 2.899 | 15.512 | 17 | O | N | B:224:_:GLY |  |  |  | 1 |
| B:221:_:LEU | HBOND:MC_MC | B:225:_:LEU | 3.215 | 55.769 | 17 | O | N | B:225:_:LEU |  |  |  | 1 |
| B:221:_:LEU | HBOND:MC_SC | B:246:_:ASP | 3.059 | 11.48 | 17 | N | OD2 | B:221:_:LEU |  |  |  | 1 |
| B:221:_:LEU | VDW:SC_SC | B:246:_:ASP | 3.76 | NaN | 6 | CD1 | CG |  |  |  |  | 1 |
| B:221:_:LEU | VDW:SC_SC | B:248:_:ALA | 3.836 | NaN | 6 | CD1 | CB |  |  |  |  | 1 |
| B:221:_:LEU | VDW:SC_SC | B:261:_:LEU | 3.856 | NaN | 6 | CD2 | CD2 |  |  |  |  | 1 |
| B:221:_:LEU | VDW:SC_SC | B:282:_:VAL | 3.738 | NaN | 6 | CD2 | CG1 |  |  |  |  | 1 |
| B:222:_:ARG | HBOND:MC_MC | B:225:_:LEU | 3.256 | 10.966 | 17 | O | N | B:225:_:LEU |  |  |  | 1 |
| B:223:_:ALA | VDW:SC_SC | B:280:_:ALA | 3.752 | NaN | 6 | CB | CB |  |  |  |  | 1 |
| B:224:_:GLY | VDW:MC_MC | B:283:_:GLY | 3.828 | NaN | 6 | C | C |  |  |  |  | 1 |
| B:226:_:GLY | HBOND:MC_MC | B:229:_:TRP | 3.14 | 24.05 | 17 | O | N | B:229:_:TRP |  |  |  | 1 |
| B:226:_:GLY | HBOND:MC_SC | B:287:_:ASP | 2.88 | 24.422 | 17 | N | OD2 | B:226:_:GLY |  |  |  | 1 |
| B:228:_:ARG | HBOND:MC_MC | B:253:_:VAL | 2.984 | 11.071 | 17 | O | N | B:253:_:VAL |  |  |  | 1 |
| B:228:_:ARG | VDW:MC_SC | B:253:_:VAL | 3.749 | NaN | 6 | C | CG2 |  |  |  |  | 1 |
| B:228:_:ARG | VDW:SC_MC | B:291:_:GLY | 3.75 | NaN | 6 | CZ | C |  |  |  |  | 1 |
| B:229:_:TRP | VDW:SC_SC | B:250:_:LEU | 3.732 | NaN | 6 | CE3 | CB |  |  |  |  | 1 |
| B:229:_:TRP | VDW:SC_MC | B:251:_:TRP | 3.815 | NaN | 6 | CE3 | C |  |  |  |  | 1 |
| B:229:_:TRP | VDW:SC_SC | B:252:_:PRO | 3.896 | NaN | 6 | CZ3 | CB |  |  |  |  | 1 |
| B:229:_:TRP | VDW:SC_SC | B:290:_:ILE | 3.639 | NaN | 6 | CZ2 | CD1 |  |  |  |  | 1 |
| B:230:_:ARG | HBOND:MC_MC | B:251:_:TRP | 2.727 | 5.247 | 17 | N | O | B:230:_:ARG |  |  |  | 1 |
| B:230:_:ARG | HBOND:MC_MC | B:251:_:TRP | 2.923 | 5.521 | 17 | O | N | B:251:_:TRP |  |  |  | 1 |
| B:232:_:GLY | HBOND:MC_MC | B:249:_:VAL | 2.807 | 13.266 | 17 | O | N | B:249:_:VAL |  |  |  | 1 |
| B:232:_:GLY | HBOND:MC_MC | B:249:_:VAL | 2.975 | 9.881 | 17 | N | O | B:232:_:GLY |  |  |  | 1 |
| B:233:_:ASP | VDW:SC_SC | B:248:_:ALA | 3.817 | NaN | 6 | CB | CB |  |  |  |  | 1 |
| B:234:_:LYS | HBOND:MC_MC | B:247:_:ILE | 2.925 | 15.034 | 17 | N | O | B:234:_:LYS |  |  |  | 1 |
| B:234:_:LYS | HBOND:MC_MC | B:247:_:ILE | 3.036 | 18.401 | 17 | O | N | B:247:_:ILE |  |  |  | 1 |
| B:236:_:GLY | HBOND:MC_MC | B:245:_:ASN | 2.711 | 11.301 | 17 | O | N | B:245:_:ASN |  |  |  | 1 |
| B:236:_:GLY | HBOND:MC_MC | B:245:_:ASN | 2.988 | 24.081 | 17 | N | O | B:236:_:GLY |  |  |  | 1 |
| B:237:_:SER | VDW:SC_SC | B:272:_:TYR | 3.784 | NaN | 6 | CB | CE1 |  |  |  |  | 1 |
| B:238:_:ASN | HBOND:SC_MC | B:242:_:ASP | 3.158 | 41.182 | 17 | OD1 | N | B:242:_:ASP |  |  |  | 1 |
| B:238:_:ASN | HBOND:SC_MC | B:243:_:ALA | 2.959 | 15.355 | 17 | OD1 | N | B:243:_:ALA |  |  |  | 1 |
| B:238:_:ASN | HBOND:MC_MC | B:243:_:ALA | 3.254 | 18.825 | 17 | N | O | B:238:_:ASN |  |  |  | 1 |
| B:238:_:ASN | VDW:SC_SC | B:243:_:ALA | 3.485 | NaN | 6 | ND2 | CB |  |  |  |  | 1 |
| B:242:_:ASP | VDW:SC_SC | B:266:_:GLN | 3.674 | NaN | 6 | CG | OE1 |  |  |  |  | 1 |
| B:242:_:ASP | HBOND:MC_MC | B:267:_:ALA | 2.951 | 24.604 | 17 | O | N | B:267:_:ALA |  |  |  | 1 |
| B:242:_:ASP | VDW:SC_MC | B:267:_:ALA | 3.745 | NaN | 6 | CB | C |  |  |  |  | 1 |
| B:244:_:ARG | HBOND:MC_MC | B:265:_:LEU | 2.872 | 15.452 | 17 | N | O | B:244:_:ARG |  |  |  | 1 |
| B:244:_:ARG | HBOND:MC_MC | B:265:_:LEU | 3.051 | 50.413 | 17 | O | N | B:265:_:LEU |  |  |  | 1 |
| B:244:_:ARG | VDW:SC_SC | B:272:_:TYR | 3.376 | NaN | 6 | CZ | CZ |  |  |  |  | 1 |
| B:244:_:ARG | VDW:SC_SC | B:272:_:TYR | 3.685 | NaN | 6 | CD | CE1 |  |  |  |  | 1 |
| B:244:_:ARG | VDW:SC_SC | B:275:_:ARG | 3.638 | NaN | 6 | CB | CB |  |  |  |  | 1 |
| B:244:_:ARG | VDW:SC_SC | B:276:_:ALA | 3.618 | NaN | 6 | CZ | CB |  |  |  |  | 1 |
| B:244:_:ARG | VDW:SC_SC | B:279:_:LEU | 3.722 | NaN | 6 | CG | CD1 |  |  |  |  | 1 |
| B:245:_:ASN | VDW:SC_SC | B:264:_:TYR | 3.211 | NaN | 6 | CG | CD2 |  |  |  |  | 1 |
| B:245:_:ASN | HBOND:SC_MC | B:265:_:LEU | 3.256 | 16.84 | 17 | OD1 | N | B:265:_:LEU |  |  |  | 1 |
| B:245:_:ASN | VDW:MC_SC | B:279:_:LEU | 3.785 | NaN | 6 | C | CD1 |  |  |  |  | 1 |
| B:246:_:ASP | HBOND:MC_MC | B:263:_:ALA | 2.822 | 18.66 | 17 | N | O | B:246:_:ASP |  |  |  | 1 |
| B:246:_:ASP | HBOND:MC_MC | B:263:_:ALA | 2.883 | 41.438 | 17 | O | N | B:263:_:ALA |  |  |  | 1 |
| B:247:_:ILE | VDW:SC_SC | B:262:_:THR | 3.844 | NaN | 6 | CG2 | CG2 |  |  |  |  | 1 |
| B:247:_:ILE | VDW:SC_SC | B:264:_:TYR | 3.613 | NaN | 6 | CG2 | CE1 |  |  |  |  | 1 |
| B:248:_:ALA | HBOND:MC_MC | B:261:_:LEU | 2.805 | 37.1 | 17 | N | O | B:248:_:ALA |  |  |  | 1 |
| B:248:_:ALA | HBOND:MC_MC | B:261:_:LEU | 2.883 | 5.862 | 17 | O | N | B:261:_:LEU |  |  |  | 1 |
| B:250:_:LEU | HBOND:MC_MC | B:259:_:TRP | 2.78 | 6.332 | 17 | N | O | B:250:_:LEU |  |  |  | 1 |
| B:250:_:LEU | HBOND:MC_MC | B:259:_:TRP | 2.925 | 12.835 | 17 | O | N | B:259:_:TRP |  |  |  | 1 |
| B:252:_:PRO | HBOND:MC_MC | B:255:_:GLY | 2.912 | 16.621 | 17 | O | N | B:255:_:GLY |  |  |  | 1 |
| B:252:_:PRO | VDW:SC_MC | B:255:_:GLY | 3.52 | NaN | 6 | CG | C |  |  |  |  | 1 |
| B:252:_:PRO | VDW:SC_MC | B:256:_:GLY | 3.578 | NaN | 6 | CG | C |  |  |  |  | 1 |
| B:259:_:TRP | VDW:SC_SC | B:290:_:ILE | 3.688 | NaN | 6 | CE2 | CG2 |  |  |  |  | 1 |
| B:259:_:TRP | VDW:SC_SC | B:290:_:ILE | 3.843 | NaN | 6 | CD2 | CD1 |  |  |  |  | 1 |
| B:261:_:LEU | VDW:SC_SC | B:286:_:ALA | 3.673 | NaN | 6 | CD2 | CB |  |  |  |  | 1 |
| B:263:_:ALA | VDW:SC_SC | B:279:_:LEU | 3.875 | NaN | 6 | CB | CD2 |  |  |  |  | 1 |
| B:263:_:ALA | VDW:SC_SC | B:282:_:VAL | 3.856 | NaN | 6 | CB | CG1 |  |  |  |  | 1 |
| B:265:_:LEU | VDW:SC_SC | B:278:_:VAL | 3.487 | NaN | 6 | CD2 | CG1 |  |  |  |  | 1 |
| B:267:_:ALA | VDW:SC_SC | B:270:_:ILE | 3.653 | NaN | 6 | CB | CG1 |  |  |  |  | 1 |
| B:270:_:ILE | VDW:SC_SC | B:274:_:GLN | 3.235 | NaN | 6 | CB | OE1 |  |  |  |  | 1 |
| B:270:_:ILE | VDW:SC_MC | B:274:_:GLN | 3.871 | NaN | 6 | CD1 | C |  |  |  |  | 1 |
| B:270:_:ILE | VDW:SC_SC | B:278:_:VAL | 3.705 | NaN | 6 | CD1 | CG2 |  |  |  |  | 1 |
| B:271:_:SER | HBOND:MC_SC | B:274:_:GLN | 2.877 | 14.901 | 17 | N | OE1 | B:271:_:SER |  |  |  | 1 |
| B:271:_:SER | HBOND:MC_MC | B:274:_:GLN | 2.986 | 39.166 | 17 | O | N | B:274:_:GLN |  |  |  | 1 |
| B:271:_:SER | HBOND:SC_MC | B:274:_:GLN | 3.142 | 26.629 | 17 | OG | N | B:274:_:GLN |  |  |  | 1 |
| B:271:_:SER | HBOND:MC_MC | B:275:_:ARG | 2.914 | 39.957 | 17 | O | N | B:275:_:ARG |  |  |  | 1 |
| B:272:_:TYR | HBOND:MC_MC | B:275:_:ARG | 3.079 | 29.152 | 17 | O | N | B:275:_:ARG |  |  |  | 1 |
| B:272:_:TYR | HBOND:MC_MC | B:276:_:ALA | 3.268 | 32.602 | 17 | O | N | B:276:_:ALA |  |  |  | 1 |
| B:273:_:GLU | HBOND:MC_MC | B:276:_:ALA | 3.123 | 32.411 | 17 | O | N | B:276:_:ALA |  |  |  | 1 |
| B:273:_:GLU | HBOND:MC_MC | B:277:_:SER | 3.128 | 35.211 | 17 | O | N | B:277:_:SER |  |  |  | 1 |
| B:274:_:GLN | HBOND:MC_MC | B:277:_:SER | 3.012 | 36.213 | 17 | O | N | B:277:_:SER |  |  |  | 1 |
| B:274:_:GLN | HBOND:MC_MC | B:278:_:VAL | 2.95 | 24.671 | 17 | O | N | B:278:_:VAL |  |  |  | 1 |
| B:275:_:ARG | HBOND:MC_MC | B:278:_:VAL | 3.158 | 44.187 | 17 | O | N | B:278:_:VAL |  |  |  | 1 |
| B:275:_:ARG | HBOND:MC_MC | B:279:_:LEU | 3.117 | 29.698 | 17 | O | N | B:279:_:LEU |  |  |  | 1 |
| B:276:_:ALA | HBOND:MC_MC | B:279:_:LEU | 3.042 | 37.644 | 17 | O | N | B:279:_:LEU |  |  |  | 1 |
| B:276:_:ALA | HBOND:MC_MC | B:280:_:ALA | 2.864 | 28.07 | 17 | O | N | B:280:_:ALA |  |  |  | 1 |
| B:277:_:SER | HBOND:MC_MC | B:280:_:ALA | 3.414 | 38.936 | 17 | O | N | B:280:_:ALA |  |  |  | 1 |
| B:277:_:SER | HBOND:MC_MC | B:281:_:GLN | 3.006 | 30.21 | 17 | O | N | B:281:_:GLN |  |  |  | 1 |
| B:278:_:VAL | HBOND:MC_MC | B:281:_:GLN | 3.191 | 40.918 | 17 | O | N | B:281:_:GLN |  |  |  | 1 |
| B:278:_:VAL | HBOND:MC_MC | B:282:_:VAL | 2.886 | 31.407 | 17 | O | N | B:282:_:VAL |  |  |  | 1 |
| B:279:_:LEU | HBOND:MC_MC | B:282:_:VAL | 3.054 | 44.741 | 17 | O | N | B:282:_:VAL |  |  |  | 1 |
| B:279:_:LEU | VDW:MC_SC | B:282:_:VAL | 3.886 | NaN | 6 | C | CB |  |  |  |  | 1 |
| B:279:_:LEU | HBOND:MC_MC | B:283:_:GLY | 2.988 | 23.505 | 17 | O | N | B:283:_:GLY |  |  |  | 1 |
| B:280:_:ALA | HBOND:MC_MC | B:283:_:GLY | 3.031 | 44.325 | 17 | O | N | B:283:_:GLY |  |  |  | 1 |
| B:280:_:ALA | HBOND:MC_MC | B:284:_:ARG | 2.979 | 33.652 | 17 | O | N | B:284:_:ARG |  |  |  | 1 |
| B:281:_:GLN | HBOND:MC_MC | B:284:_:ARG | 3.209 | 36.629 | 17 | O | N | B:284:_:ARG |  |  |  | 1 |
| B:281:_:GLN | HBOND:MC_MC | B:285:_:ILE | 2.992 | 17.737 | 17 | O | N | B:285:_:ILE |  |  |  | 1 |
| B:282:_:VAL | HBOND:MC_MC | B:285:_:ILE | 3.42 | 43.641 | 17 | O | N | B:285:_:ILE |  |  |  | 1 |
| B:282:_:VAL | HBOND:MC_MC | B:286:_:ALA | 2.971 | 30.639 | 17 | O | N | B:286:_:ALA |  |  |  | 1 |
| B:283:_:GLY | HBOND:MC_MC | B:286:_:ALA | 3.403 | 40.453 | 17 | O | N | B:286:_:ALA |  |  |  | 1 |
| B:283:_:GLY | HBOND:MC_MC | B:287:_:ASP | 2.833 | 29.423 | 17 | O | N | B:287:_:ASP |  |  |  | 1 |
| B:284:_:ARG | HBOND:MC_MC | B:287:_:ASP | 3.326 | 38.863 | 17 | O | N | B:287:_:ASP |  |  |  | 1 |
| B:284:_:ARG | HBOND:MC_MC | B:288:_:ARG | 3.165 | 29.214 | 17 | O | N | B:288:_:ARG |  |  |  | 1 |
| B:285:_:ILE | HBOND:MC_MC | B:288:_:ARG | 3.177 | 36.362 | 17 | O | N | B:288:_:ARG |  |  |  | 1 |
| B:285:_:ILE | HBOND:MC_MC | B:289:_:LEU | 2.96 | 29.398 | 17 | O | N | B:289:_:LEU |  |  |  | 1 |
| B:286:_:ALA | HBOND:MC_MC | B:289:_:LEU | 3.058 | 39.461 | 17 | O | N | B:289:_:LEU |  |  |  | 1 |
| B:286:_:ALA | HBOND:MC_MC | B:290:_:ILE | 3.037 | 30.239 | 17 | O | N | B:290:_:ILE |  |  |  | 1 |
| B:287:_:ASP | HBOND:MC_MC | B:291:_:GLY | 3.395 | 23.326 | 17 | O | N | B:291:_:GLY |  |  |  | 1 |
| B:288:_:ARG | HBOND:MC_MC | B:291:_:GLY | 3.313 | 42.865 | 17 | O | N | B:291:_:GLY |  |  |  | 1 |
|  |  |  |  |  |  |  |  |  |  |  |  |  |
| HBOND - Hydrogen Bond, (MC) - Main Chain | | | | | | | | | | | | |
| VDW - Van der Waals interactions (C-C) - Carbon-Carbon, (C-S) Carbon-Sulfur | | | | | | | | | | | | |
| PIPISTACK - π-π stacking | | | | | | | | | | | | |
| PICATION - π-cation | | | | | | | | | | | | |


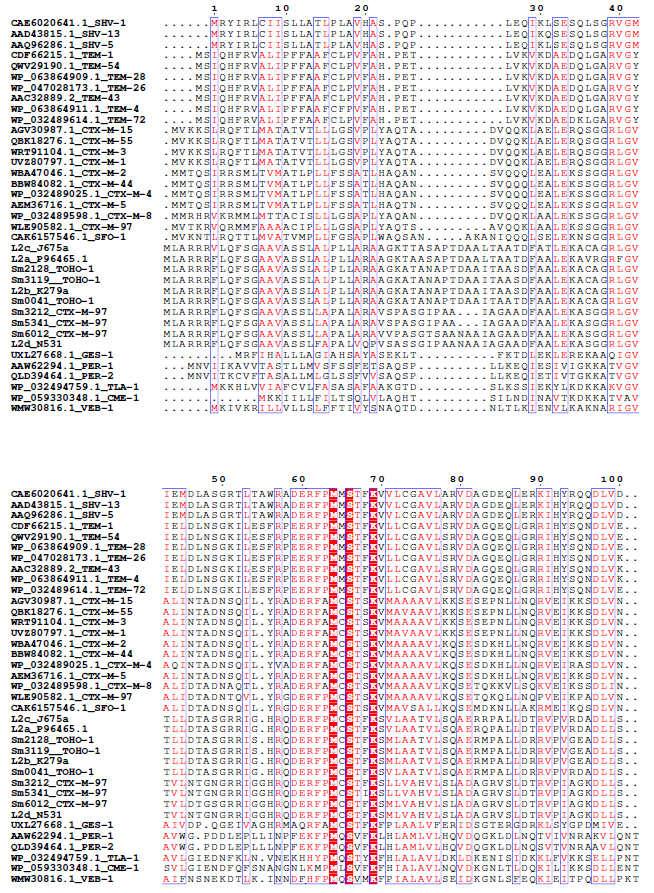


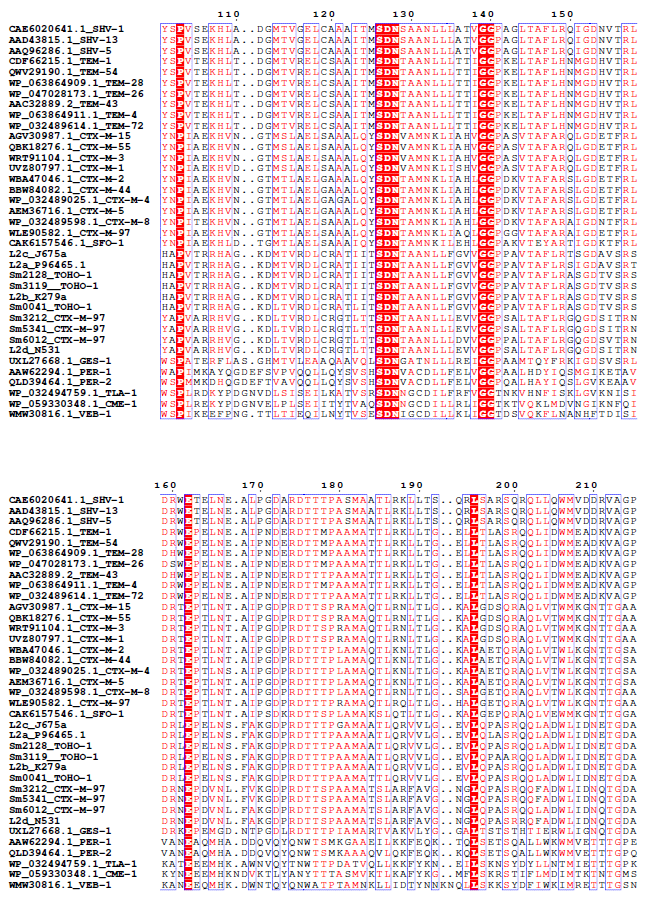


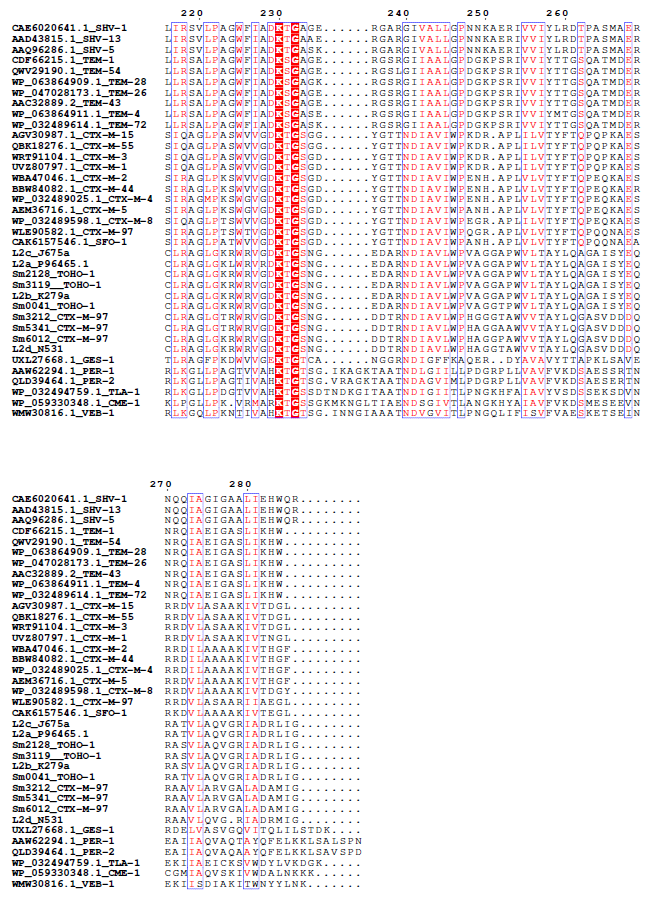


**Figure S3:** A multiple sequence alignment of Class A beta-lactamases with L2 reference proteins (10, 14) and six L2 beta-lactamases assessed in this study by cloning. Highly conserved residues common to all Class A beta-lactamases are highlighted in red.
